# Isoprene Polymerization: Catalytic Performance of Iminopyridine Vanadium(III) Chloride *versus* Vanadium(III) Chloride

**DOI:** 10.3390/polym11071122

**Published:** 2019-07-02

**Authors:** Mengmeng Zhao, Qaiser Mahmood, Chuyang Jing, Liang Wang, Guangqian Zhu, Xianhui Zhang, Qinggang Wang

**Affiliations:** 1 Key Laboratory of Biobased Materials, Qingdao Institute of Bioenergy and Bioprocess Technology, Chinese Academy of Sciences, Qingdao 266000, China; 2 Center of Materials Science and Optoelectronics Engineering, University of Chinese Academy of Sciences, Beijing 100049, China

**Keywords:** polyisoprene, iminopyridine ligands, vanadium complexes, VCl_3_/MAO, Ziegler-Natta polymerization

## Abstract

A series of vanadium complexes bearing iminopyridine bidentate ligands with various electronic and steric properties: **V1** [CH_2_Ph], **V2** [CMe_2_CH_2_CMe_3_], **V3** [Ph] and **V4** [2,6-*^i^*Pr_2_Ph] were prepared and characterized by IR spectroscopy and microanalytical analysis. The catalytic capacity of all the complexes has been investigated for isoprene polymerization and was controlled by tuning the ligand structure with different *N*-alkyl and *N*-aryl groups. Activated by methylaluminoxane (MAO), the aryl-substituted complex **V3** [Ph] exhibited high *cis*-1,4 selectivity (75%), and the resultant polymers had high molecular weights (*M_n_* = 6.6 × 10^4^) and narrow molecular weight distributions (PDI = 2.3). This catalyst showed high activity up to 734.4 kg polymer (mol V)^−1^ h^−1^ with excellent thermostability even stable at 70 °C. Compared to the traditional VCl_3_/MAO catalytic system, iminopyridine-supported V(III) catalysts displayed higher catalytic activities and changed the selectivity of monomer enchainment from *trans*-1,4 to *cis*-1,4.

## 1. Introduction

Polydienes, a class of multipurpose elastomers which play a vital role in modern society, have attracted considerable attention of both industrial and academic communities. Advantages typical of both rubber and plastic materials make them superior over vulcanized rubber, therefore, these polymeric materials are widely employed as commodity material in tires, medical materials, sporting goods etc. [1,2,3,4,5]. Generally, polydienes are manufactured by the polymerization of conjugated dienes. Among various polydiene products, polyisoprenes are identified as important synthetic elastomer, due to their diversified microstructures [1,2, 3,4, *cis*-1,4 and *trans*-1,4 stereoregularities in polymer] and thereby, a strong influence on the properties of the resulting polymer. For example, natural rubber mainly consists of *cis*-1,4 polyisoprene, and exhibits similar properties to the synthetic *cis*-1,4 regular polyisoprene, while *trans*-1,4 polyisoprene is nearly identical to the gutta-percha rubber or balata rubber [6,7].

Given the massive global demand of polyisoprene and limited supply of natural rubbers trigger many researchers to develop more reactive and controllable catalytic system either by changing the metal center of the catalysts or controlling the reaction conditions [8,9,10]. Regarding metal-based catalysts, the mainstream of catalysts relied on early transition metals ranging from group III to IV. For example, rare-earth metal and titanium catalysts afforded *cis*-1,4 or *trans*-1,4 polyisoprene with up to 98% selectivity [11,12,13,14,15]. In addition, some late transition-metal catalysts were also studied for isoprene polymerization, such as well-defined Fe and Co complexes [16,17,18,19,20,21,22,23]. However, the polymerization of isoprene with mid-transition metal-based catalysts remains less explored. Vanadium-based catalysts, discovered in the 1950s, are used as the first homogeneous catalyst system for olefin polymerization [24]. After that, numerous vanadium-based catalysts for stereospecific polymerization of olefin have been intensively explored [25,26,27,28].

Specifically for isoprene polymerization, various vanadium metal-based catalytic systems have been studied. For instance, the combination of VCl_3_, VCl_4_, or VOCl_3_ with alkylaluminium, either in heterogeneous or homogeneous nature were extensively studied and yielded polyisoprene with high *trans*-1,4 structures [29,30,31,32,33,34,35]. However, the main disadvantage of these catalysts is that their active species can easily undergo deactivation, and, thus, affording low activities [36,37]. To address this issue, researchers focused to developing well-defined vanadium catalysts by introducing the ligand into the structure. To this end, numerous well-defined vanadium complexes have been investigated as catalysts for ethylene polymerization, however, few reports are available in which conjugated diene polymerization is explored [38,39,40,41,42]. In 2016s, the homogeneous catalytic system: Vanadium-[ONNO] complexes combined with *n*-butyl ethyl magnesium or triisobutyl aluminium, had been reported for isoprene polymerization. In this study, obtained polyisoprene was composed of 70% 3,4- and 30% 1,4-units [41]. Later in 2017s, Ricci et al. reported iminopyridine-vanadium complexes for isoprene polymerization showed good activities and produced *cis*-1,4 enriched polyisoprene [42].

It is noticed that iminopyridine ligands are widely studied for many catalytic reactions, due to their facile synthesis and easy to control the catalytic performance by means of different substituents [19,20,21,23]. To extend this study, herein, we reported the synthesis of a series of iminopyridine vanadium(III) complexes and employed as catalysts for isoprene polymerization. By means of different cocatalysts and reaction conditions, namely catalyst loadings, polymerization temperature and reaction time, the performance of title catalysts in term of activity and stereoselectivity of monomer enchainment were systematically investigated. Meanwhile, an in-depth isoprene polymerization was conducted using traditional Ziegler-Natta catalyst composed of VCl_3_ and methylaluminoxane (MAO), and compared the results with iminopyridine-vanadium catalyzed isoprene polymerization. The ligand modificationwith different substituents and reaction conditionat differet reaction temperatures, exhibited remarkable effects on the stereoselectivity of monomer enchainment.

## 2. Materials and Methods

### 2.1. Materials

All the manipulations of air/moisture sensitive compounds were carried out under argon atmosphere using standard Schlenk technique or in glove box. Toluene was refluxed over sodium for 6 h, then distilled and stored over molecular sieves under nitrogen. Hexane and dichloromethane were refluxed over calcium hydride for 6 h and distilled and stored over molecular sieves under argon conditions. Isoprene was purchased from Aladdin Co. (Shanghai, China) and was purified by distillation over calcium hydride before use. VCl_3_ and VCl_3_(THF)_3_ were purchased from Sigma-Aldrich Co. (Saint Louis, MO, USA). Methylaluminoxane (MAO, 1.5 M solution in toluene) was purchased from Aike Reagent Co (Chengdu, China). Triethylaluminum (AlEt_3_, 1.0 M solution in toluene) and triisobutylaluminum (Al(*i*-Bu)_3_, 1.0 M solution in toluene) were purchased from Aladdin Co. (Shanghai, China). Other reagents were purchased from commercial sources and used without purification.

### 2.2. Synthesis of ligand ***L1***–***L4***

General procedure: A solution of the corresponding amine (1.0 equiv.) in dichloromethane was added to a solution of pyridine-2-carbaldehyde (1.0 equiv.) in dichloromethane containing 4Å molecular sieve (300 mg). The mixture was stirred at room temperature. After overnight stirring, the reaction mixture was filtered, and the resulting solution was concentrated under reduced pressure to obtain the corresponding ligand [19].

1-Phenyl-*N*-(pyridin-2-ylmethylene)methanamine (**L1**): Yellow oil. Yield: 2.5 g (68%). ^1^H NMR (400 MHz, CDCl_3_, 298 K) δ 8.64–8.63 (m, 1H), 8.49 (s, 1H), 8.06 (d, *J* = 7.6 Hz, 1H), 7.74–7.69 (m, 1H), 7.74–7.69 (m, 6H), 4.87 (s, 2H). ^13^C NMR (100 MHz, CDCl_3_, 298 K) δ 162.9, 154.6, 149.4, 138.7, 136.6, 128.6, 128.2, 127.2, 124.8, 121.4, 65.0.

2,4,4-Trimethyl-*N*-(pyridin-2-ylmethylene)pentan-2-amine (**L2**): Light yellow oil. Yield: 1.6 g (80%). ^1^H NMR (400 MHz, CDCl_3_, 298 K) δ 8.64–8.59 (m, 1H), 8.34 (s, 1H), 8.04–8.02 (m, 1H), 7.74–7.70 (m, 1H), 7.30–7.27 (m, 1H), 1.71 (s, 2H), 1.33 (s, 6H), 0.95 (s, 9H). ^13^C NMR (100 MHz, CDCl_3_, 298 K) δ 155.9, 155.8, 149.2, 136.6, 124.3, 120.7, 61.6, 56.5, 32.1, 31.8, 29.6.

*N*-(pyridin-2-ylmethylene)aniline (**L3**): Yellow oil. Yield: 1.3 g (78%). ^1^H NMR (400 MHz, CDCl_3_, 298 K) δ: 8.75–8.69 (m, 1H), 8.61 (s, 1H), 8.21 (d, *J* = 8.0 Hz, 1H), 7.84–7.80 (m, 1H), 7.46–7.35 (m, 3H), 7.31–7.27 (m, 3H). ^13^C NMR (100 MHz, CDCl_3_, 298 K) δ 160.6, 154.5, 150.9, 149.6, 136.7, 129.2, 126.7, 125.1, 121.9, 121.1.

2,6-Diisopropyl-*N*-(pyridin-2-ylmethylene)aniline (**L4**): Light yellow oil. Yield: 1.2 g (32%). ^1^H NMR (400 MHz, CDCl_3_, 298 K) δ 8.75–8.73 (m, 1H), 8.32 (s, 1H), 8.29–8.26 (m, 1H), 7.88–7.82 (m, 1H), 7.43–7.40 (m, 1H), 7.20–7.10 (m, 3H), 3.03–2.92 (m, 2H), 1.18 (d, *J* = 7.2 Hz, 12H). ^13^C NMR (100 MHz, CDCl_3_, 298 K) δ 163.1, 154.5, 149.8, 148.5, 137.4, 136.9, 125.5, 124.1, 123.2, 121.5, 28.1, 23.6.

### 2.3. Synthesis of Vanadium Complexes ***V1–V4***

All complexes were prepared under nitrogen atmosphere. The equimolar solution of VCl_3_(THF)_3_ and corresponding ligands in anhydrous dichloromethane (10 mL) was stirred for 24 h at room temperature. Dark solids were filtered off from the solution. The filtrate was concentrated to 2 mL, layered with 5 mL hexane and was then cooled to 0 °C for 4 h. The precipitates were filtered and washed with hexane, dried under vacuum to give the vanadium complexes [39].

(Benzyl Iminopyridine)VCl_3_ (**V1**): A mixture of **L1** (105.1 mg, 0.54 mmol) and anhydrous VCl_3_(THF)_3_ (0.2 g, 0.54 mmol) was stirred to give **V1** as yellow solid (152.0 mg, 67%). ATR-IR (cm^−1^): 3059, 1599 ν(C=N), 1454, 1305, 1053, 1026, 986, 758, 703. Anal. Calcd. For C_13_H_12_Cl_3_N_2_V·1/2(CH_2_Cl_2_): C, 40.94; H, 3.31; N, 7.07; found: C, 40.59; H, 4.62; N, 7.13.

(Octyl Iminopyridine)VCl_3_ (**V2**): A mixture of **L2** (116.8 mg, 0.54 mmol) and anhydrous VCl_3_(THF)_3_ (0.2 g, 0.54 mmol) was stirred to give **V2** as yellow solid (70.0 mg, 29%). ATR-IR (cm^−1^): 3093, 1609 ν(C=N), 1483, 1032, 985, 773. Anal. Calcd. For C_14_H_22_Cl_3_N_2_V: C, 44.76; H, 5.90; N, 7.46; found: C, 45.38; H, 5.99; N, 7.32.

(Phenyl Iminopyridine)VCl_3_ (**V3**): A mixture of **L3** (97.5 mg, 0.54 mmol) and anhydrous VCl_3_(THF)_3_ (0.2 g, 0.54 mmol) was stirred to give **V3** as pink solid (130.0 mg, 59%). ATR-IR (cm^−1^): 2959, 1592 ν(C=N), 1458, 1308, 1024, 774, 765. Anal. Calcd. For C_12_H_10_Cl_3_N_2_V·C_4_H_8_O: C, 46.69; H, 4.41; N, 6.81; found: C, 46.61; H, 3.91; N, 7.51.

(2,6-Diisopropylphenyl Iminopyridine)VCl_3_ (**V4**): A mixture of **L4** (142.6 mg, 0.54 mmol) and anhydrous VCl_3_(THF)_3_ (0.2 g, 0.54 mmol) was stirred to give **V4** as pink solid (115.0 mg, 63%). ATR-IR (cm^−1^): 3330, 1596 ν(C=N), 1487, 1025, 777, 765. Anal. Calcd. For C_18_H_22_Cl_3_N_2_V: C, 51.03; H, 5.23; N, 6.61; found: C, 49.84; H, 5.16; N, 6.43.

### 2.4. Polymerization Procedure

The polymerization of isoprene in toluene was carried out in a 25 mL Schlenk reactor. In a typical process, the reactor was heated, dried in vacuum, and recharged with argon for more than three times. The vanadium complex was weighed in a glove box and then introduced into a Schlenk reactor. The reactor was taken out of the glove box. The required amount of solvent, isoprene and aluminum cocatalyst was added in sequence into the reactor under nitrogen via a septum. After the required reaction time, the flask was opened to air, and the polymerization was quenched with a diluted HCl solution of methanol (methanol/HCl = 50/1). The resulting solution was poured into a large volume of methanol containing 2,6-bis(1,1-dimethylethyl)-4-methylphenol (BHT) as the stabilizing agent. The polymer was collected by filtration and washed with ethanol several times and dried at room temperature under vacuum. The polymer yields were determined by gravimetry.

### 2.5. Polymer Characterization

The number-average and weight-average molecular weights (*M_n_* and *M*_w_) and molecular weight distributions (*M*_w_/*M_n_*) of polymers were measured by gel permeation chromatography (GPC) using a PL-GPC 220 chromatography. Plgel MIXED-B LS 300 mm × 7.5 mm column was employed and maintained at 150 °C. Trichlorobenzene stabilized with 0.0125% BHT used as eluent at a flow rate of 1.0 mL/min. The molecular weights of polyisoprene were determined by using a polystyrene calibration. The injection volume of the sample solution was 200 μL. The microstructure of polyisoprene was determined by NMR spectra on a Bruker Advance 400 spectrometer at 298 K. ^1^H NMR, and ^13^C NMR spectra of ligands and polyisoprene were recorded in CDCl_3_. The polyisoprene microstructure of the 1,4 and 3,4 ratio was determined from ^1^H NMR of the 1,4 =CH signals at 5.15 ppm and the 3,4 =CH_2_ signal at 4.7 ppm. The *trans*/*cis*-1,4 stereoisomer ratio was determined from ^13^C NMR of the –CH_3_ signals of *cis*-1,4 at 23.8 ppm and *trans*-1,4 at 16.3 ppm.

## 3. Results

### 3.1. Synthesis and Characterization of Iminopyridine Vanadium(III) Complexes

**Scheme 1:**
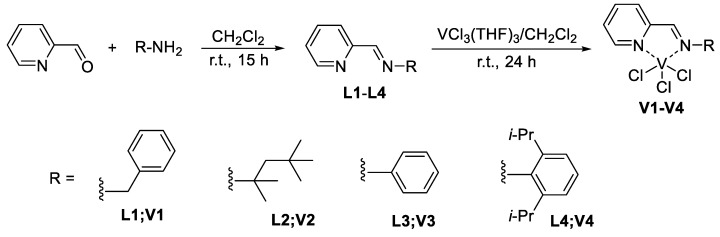
Synthesis of iminopyridine ligands and their V(III) complexes.

The general synthetic method of new vanadium complexes used in this study is shown in Scheme 1. The iminopyridine ligands (**L1**–**L4**) were synthesized by following the reported procedure, and their structures were identified by ^1^H and ^13^C NMR. The reaction of VCl_3_(THF)_3_, with 1.0 equivalent of corresponding iminopyridine ligand (**L1**–**L4**) in CH_2_Cl_2_ solution, afforded a set of four vanadium complexes in moderate yields (**V1**, 67%; **V2**, 29%; **V3**, 59%; **V4**, 63%). All these complexes were characterized by ATR-IR and elemental analysis. However, our multiple attempts to obtain the suitable single crystals for X-ray diffraction and high resolution mass spectrum analyses of all these complexes were failed, due to their relatively high instability in air and moisture. Indeed, crystals were also not obtained in a glove box. Because of the paramagnetic nature of these vanadium complexes, the NMR also did not give clear information.

### 3.2. Isoprene Polymerization Studies Catalyzed by VCl_3_/MAO

Initially, traditional Ziegler-Natta catalyst, i.e., VCl_3_/alkyl aluminum, was employed to optimize the reaction conditions for the polymerization of isoprene. For this, initially, the effects of different cocatalysts and reaction solvents were examined, and the obtained results are presented in Table S1 (Supplementary Materials). Specifically, three cocatalysts, namely methylaluminoxane (MAO), triehtylaluminium (AlEt_3_), and triisobutylaluminium Al(*i*-Bu)_3_, were evaluated. At 50 °C, VCl_3_ in combination with MAO displayed the highest activity for a run time of 15 h and the resultant polymer possessed high molecular weight (*M_n_* = 1.2 × 10^5^ g/mol), however, relatively broad molecular weight distributions (*M*_w_/*M_n_* = 2.3) was identified as compared to the polymer obtained in the presence of AlEt_3_ (*M*_w_/*M_n_* = 1.8) cocatalyst (Table S1, entries 1 and 2). Regarding the microstructural properties, compared to the polyisoprene obtained with AlEt_3_ (98% of *trans*-1,4 units) and Al(*i*-Bu)_3_ (>99% of *trans*-1,4 units) cocatalysts, slightly poor selectivities were identified for the VCl_3_/MAO induced polyisoprene: Eighty-three percent *trans*-1,4 units. Taking the activity, *M_n_* and *M*_w_/*M_n_* into consideration, MAO was chosen as the optimal cocatalyst for further isoprene polymerization tests. Polymerization tests were performed in polar and non-polar solvents, such as hexane, toluene and dichloromethane, to study the effect of the reaction medium. Polymerization in toluene gave relatively higher yields with comparatively high stereoselectivities of polymer than the polymerization performed in hexane. (Table S1, entry 5, Supplementary Materials). However, some white powder with good solubility in CDCl_3_ was collected when polymerization performed in dichloromethane. The ^1^H and ^13^C NMR spectra of this white powder showed no characteristic signal of polyisoprene which indicated no polymer was formed at all in dichloromethane (Table S1, entry 4, Supplementary Materials). Therefore, toluene was found as the best reaction medium and used for further examinations.

**Figure 1:**
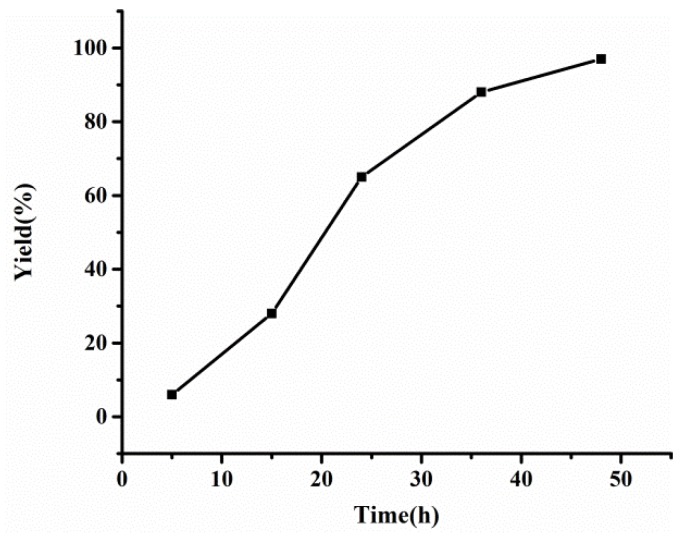
Plot of reaction time vs. yield of the polyisoprene obtained in Table 1, entries 3−7.

To further explore the catalytic scope of VCl_3_/MAO system, three more reaction parameters, namely reaction temperature, time, and the amount of cocatalyst, were tested—the results are given in Table 1. Initially, the thermo-stability of active species was examined at different reaction temperatures (e.g., 25, 50 and 70 °C) with [V]/[IP]/[Al] ratio fixed at 1/200/30 (Table 1, entries 1–3). The highest yield of 88% was found at 25 °C, which slightly improved to 90% at 50 °C; however, this yield dramatically fell to 65% at 70 °C for the run time of 36 h. This lower yield at elevated temperature would be due to the partial deactivation of active species [43]. Meanwhile, regioselectivity of monomer enchainment was decreased on increasing the reaction temperature: Polymer obtained at 25 °C possessed the highest amount of *trans*-1,4 regularities (>99%). By considering the high regioselectivity at a lower temperature, a reaction temperature of 25 °C was used to examine the lifetime of active species. For this, polymerization tests were performed for different run times, namely 5, 15, 24, 36, and 48 h—the results are given in Figure 1. The results revealed that isoprene yields were consistently increased on prolonging the reaction time and the highest yield of 97% was found for a run time of 48 h (Table 1, entry 7). This continuous growth in the yield proved that active species had long life-times and showed high stability even after 48 h. Furthermore, variation in reaction time showed no effect on the regioselectivities of the resultant polymer and in all polymer samples possessed more than 99% *trans*-1,4 units. However, the results of molecular weights and molecular weights distribution showed no clear trend. The polymerization tests were performed at different [Al]/[V] ratio (Table 1, entries 8–10) using fixed a temperature of 25 °C. On decreasing [Al]/[V] ratio from 30 to 10 led to a quantitative yield (>99%). However, a further decrease in cocatalysts loading gave lower yields (Table 1, entries 9 and 10). Interestingly, we found that even 1:1 equivalent of VCl_3_ and MAO could initiate the polymerization of isoprene (Table 1, entry 10). The relationship of the regioselectivity of isoprene polymerization with the change in [Al]/[V] ratio is trivial, and all reaction gave *trans*-1,4 polyisoprene with >99% selectivity. Whereas, lower *M_n_* and slightly broad *M*_w_/*M_n_* were observed when decreasing the [Al]/[V] ratio from 30 to 10. However, the opposite trend of *M_n_* and *M*_w_/*M_n_* were obtained on changing the Al/V ratio from 10 to 1 (Table 1, entries 9 and 10).

### 3.3. Isoprene Polymerization Catalyzed by ***V1–V4*** Complexes

**Figure 2:**
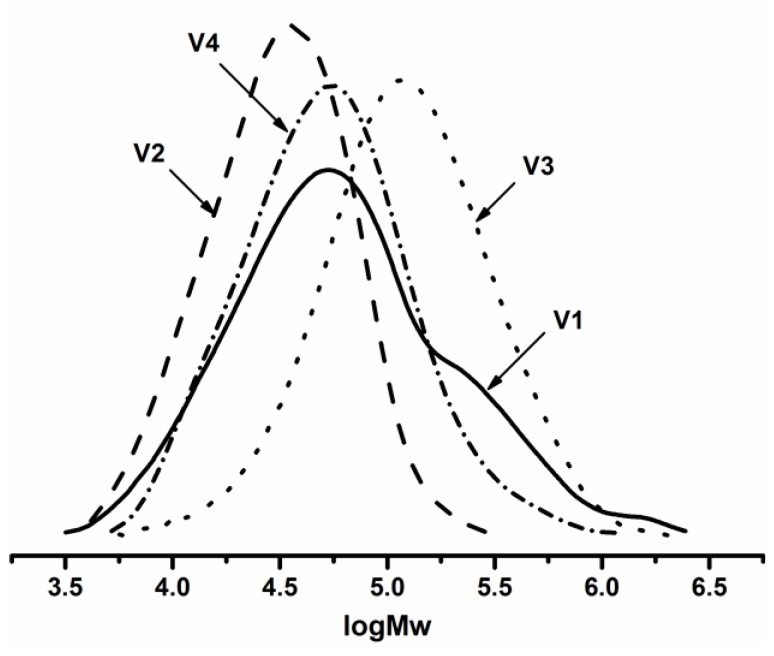
GPC curves of polyisoprene obtained using **V1**–**V4/**MAO catalyst system (Table 2, entry 1−4).

In order to investigate the catalytic capacity of iminopyridine vanadium complexes **V1**–**V4** for isoprene polymerization, MAO was employed as cocatalyst. Typical polymerization tests were performed in toluene at 50 °C and results are summarized in Table 2. All the four vanadium complexes showed higher catalytic activities than the activities observed for the VCl_3_/MAO catalytic system for a run time of 5 h. These results are highlighting the key role of ligand in enhancing the stability of active species, which thereby improve the catalytic activities. The catalytic performances of these catalysts were significantly influenced by the different *N*-alkyl/aryl-substituents of the iminopyridine ligands. For instance, catalysts **V1** [CH_2_Ph] and **V2** [Cme_2_CH_2_Cme_3_] bearing *N*-alkyl substituents displayed relatively lower activities than catalysts **V3** [Ph] and **V4** [2,6-*^i^*Pr_2_Ph] containing *N*-aryl-substituents (Table 2, entries 1, 2 vs. entries 3, 4). **V1** [CH_2_Ph] and **V2** [Cme_2_CH_2_Cme_3_] resulted 29% and 26% yields of polyisoprene respectively (Table 2, entries 1 and 2). These yields of polymer are significantly lower than the 94% and 90% obtained for the **V3** [Ph], and **V4** [2,6-*^i^*Pr_2_Ph] catalyzed isoprene polymerization respectively (Table 2, entries 1 and 2 verses 3 and 4). This difference in catalytic performance would be due to the different electronic nature of the ligand support. The *N*-aryl groups of complexes **V3** [Ph] and **V4** [2,6-*^i^*Pr_2_Ph] relatively increases the Lewis character of the active species and thereby enhances the monomer insertion. Thus, high conversions were observed for catalysts **V3** [Ph] and **V4** [2,6-*^i^*Pr_2_Ph]. Meanwhile, complex **V4** [2,6-*^i^*Pr_2_Ph] was less active catalyst than the *N*-phenyl substituted **V3**, it would be due to the more steric hindrance induced by two isopropyl substituents around the active species of **V4**. Regarding the properties of the polymer, the complex **V3** [Ph] bearing the *N*-phenyl group afforded high molecular weights polyisoprene (*M_n_* = 8.2 × 10^4^) with narrow polydispersity (Table 2, entry 3). The GPC results of the obtained polymers displayed unimodal curves, which indicated single-site active species (Figure 2).

**Figure 3:**
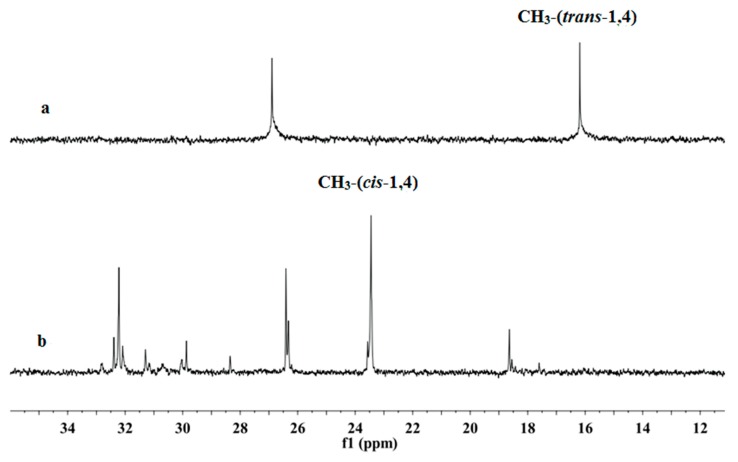
^13^C NMR spectra of polyisoprene obtained by VCl_3_/MAO (**a**: Table 1, entry 3) and **V3**/MAO (**b**: Table 2, entry 3).

The different *N*-alkyl or *N*-aryl also had significant effects on the regioselectivity of isoprene polymerization. Complexes **V1**, **V2** and **V4** all showed low selectivities as resultant polymer possessed a mixture of *trans*-1,4, *cis*-1,4 and 3,4 units. However, complex **V3** gave a high selectivity for *cis*-1,4 units and generated a polymer which was composed of *cis*-1,4/3,4 regularities with a ratio of 3:1 without *trans*-1,4 units. Meanwhile, it was found that other cocatalysts, such as AlEt_3_ and Al(*i*-Bu)_3_ combined with **V3**, were not able to catalyze the polymerization of isoprene under the same reaction conditions (Table 2, entries 5 and 6). The ^13^C NMR spectra of the polyisoprene obtained with VCl_3_/MAO (Table 1, entry 3) and **V3**/MAO (Table 2, entry 3) are displayed in Figure 3. It is observed that VCl_3_/MAO gave a polymer having 100% *trans*-1,4 units and **V3**/MAO produced polyisoprene showed high *cis*-1,4 (75%) content without the appearance of *trans*-1,4 units.

**Figure 4:**
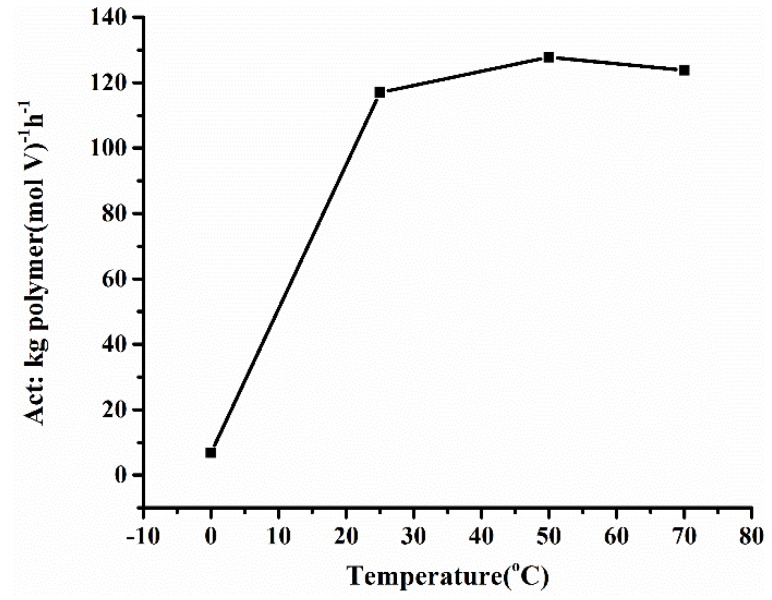
Catalytic activities of complex **V3** with MAO at different temperature.

The aim of this study was to investigate the effect of Al/V ratio and temperature on the isoprene polymerization. Therefore, the complex V3 was used as model catalysts, and the obtained results are displayed in Table 3. With temperature fixed at 50 °C, Al/V ratios varied systematically from 100 to 10 (Table 2, entry 3, Table 3, entry 1, 2). At Al/V ratio of 50 or 10, lower yields of polymer were observed. The molecular weight of the polyethylene ranging from 8.2 to 5.4 × 10^4^ and exhibited moderate polydispersity (*M*_w_/*M*_n_ = 2.2~1.9), which indicated a single-site active species. Likewise, as the ratio of Al/V decreased, the *cis*-1,4 content of polyisoprene consistently decreased, and *trans*-1,4 units were started to appear in the resultant polymer which would be due to the different active center (Table 3, entries 1 and 2). Meanwhile, the influence of temperature on the **V3** catalyzed polymerization of isoprene was also studied (Figure 4). It was found that catalytic activities slightly decreased on elevating the reaction temperature. When polymerization was performed at 70 °C, activity slightly reduced to 91%, which indicated the high thermal stability of active species induced by **V3**/MAO catalytic system (Table 3, entry 3). Further lowering the reaction temperature from 50 to 25 °C afforded slightly lower polymer yield of 86% (Table 3, entry 4). By contrast, number average molecular weights were improved on decreasing the reaction temperature to 25 °C. It can be ascribed to the lower chain transfer reaction as compared to the chain propagation on lowering the reaction temperature. The reaction performed at 0 °C resulted in a substantial decrease in polymer yield (Table 3, entry 5). It may be due to the generation of less number of active species at a temperature lower than 0 °C. When the polymerization was conducted at 0 °C, the *trans*-1,4 selectivity of the generated polymer dramatically increased from 0% to 85% even with very low polymer yield (Table 3, entry 5). In the 10 min reaction time with **V3** catalyzed isoprene polymerization, 90% yield of polyisoprene with high activity up to 734.4 kg polymer (mol V)^−1^ h^−1^ was achieved (Table 3, entry 6). Prolongation of the reaction time to 2 h resulted in full conversion, and obtained polymer showed relatively high *M_n_* value. The polymer obtained in 10 min or 2 h, showed similar stereoselectivity as 75% of *cis*-1,4 and 25% of 3,4 units.

In comparison with previous works, the title iminopyridine-vanadium complexes displayed better catalytic performance in terms of activities and selectivities. For instance, Zink and coworkers studied isoprene polymerization using vanadium chelates of dianionic tetradentate amine-bisphenolate [ONNO]. These catalysts showed lower activities as required 8 h for complete conversions. The resulting polyisoprenes were composed of 70% of 3,4 units and 30% of the mixture of *cis*-1,4 and *trans*-1,4 stereoisomers [41]. Similar results of low activities were found with vanadium catalysts bearing iminopyridine as ligand reported by Ricci in the patent, which only gave 80.7% conversion after 2 h [42]. Because vanadium catalysts reported herein could catalyze isoprene polymerization over 90% yield in 10 min, and the resulting polyisoprene was mainly composed of *cis*-1,4 (75%) units and 3,4 units(25%).

## 4. Conclusions

In summary, a library of four vanadium complexes bearing iminopyridine ligands were synthesized, and their catalytic capacity for the polymerization of isoprene was investigated in detailed. The introduction of ligand to the vanadium center significantly improved the reactivity and selectivity of the catalyst as compared to the traditional VCl_3_/MAO system. Interestingly, the *N*-phenyl substituted complex **V3** was found to be the most effective catalyst (75% of *cis*-1,4 and 25% of 3,4) which allowed for high reactivity up to 734.4 kg polymer (mol V)^−1^ h^−1^ and excellent thermostability even at 70 °C. In conclusion, this study demonstrated a family of highly effective V(III) catalysts for isoprene polymerization. Further studies of iminopyridine vanadium complexes catalyzed the polymerization of other olefins are underway.

## Figures and Tables

**Table 1:** Effects of temperature and time of VCl_3_/ methylaluminoxane (MAO) catalyst ^a^.

Entry	[V]/[IP]/[MAO]	Time (h)	Temp (°C)	Yield (%) ^b^	M_n_^c^ (×10^−4^)	M_w_/M_n_^c^	Microstructure(%) ^d^		
							trans-1,4	cis-1,4	3,4
1	1/200/30	36	50	90	5.1	2.3	83	14	3
2	1/200/30	36	70	65	0.5	2.9	68	25	7
3	1/200/30	36	25	88	7.3	1.8	>99	0	0
4	1/200/30	5	25	6	8.0	1.9	>99	0	0
5	1/200/30	15	25	28	19.3	2.0	>99	0	0
6	1/200/30	24	25	65	3.1	3.5	>99	0	0
7	1/200/30	48	25	97	3.5	2.4	>99	0	0
8	1/200/10	48	25	>99	1.2	3.5	>99	0	0
9	1/200/5	48	25	18	2.4	2.0	>99	0	0
10	1/200/1	48	25	13	5.7	1.9	>99	0	0

^a^ General condition: [Ip]_0_ = 5.0 M, V_tol._ = 2 mL. ^b^ Determined by isolated yield. ^c^ Determined by gel permeation chromatography (GPC). ^d^ Determined by ^1^H NMR and ^13^C NMR.

**Table 2:** Effects of catalyst and cocatalyst by iminopyridine vanadium complexes ^a^.

Entry	Cat.	Cocat.	Time (h)	Yield (%) ^b^	M_n_^c^ (×10^−4^)	M_w_/M_n_^c^	Microstructure(%) ^d^		
							trans-1,4	cis-1,4	3,4
1	**V1**	MAO	5	29	3.4	3.7	22	54	24
2	**V2**	MAO	5	26	2.3	1.7	36	47	17
3	**V3**	MAO	1	94	8.2	2.2	0	75	25
4	**V4**	MAO	5	92	3.6	2.2	9	67	24
5	**V3**	AlEt_3_	16	0	--	--	--	--	--
6	**V3**	Al(i-Bu)_3_	16	0	--	--	--	--	--

^a^ Polymerization in toluene (5 mL) at 50 °C, [Ip] = 4.0 mol/L, [V]/[Ip]/[cocat.] = 1/2000/100. ^b^ Determined by isolated yield. ^c^ Determined by gel permeation chromatography (GPC). ^d^ Determined by ^1^H NMR and ^13^C NMR.

**Table 3:** Effects of temperature and amount of cocatalyst by complex **V3** ^a^.

Entry	Temp.	Time	Yield (%) ^b^	M_n_^c^ (×10^−4^)	M_w_/M_n_^c^	Activity ^d^	Microstructure(%) ^e^		
							trans-1,4	cis-1,4	3,4
1 ^f^	50 °C	1 h	76	5.4	2.1	103.4	2	73	25
2 ^g^	50 °C	1 h	10	7.8	1.9	13.6	14	65	21
3	70 °C	1 h	91	6.6	2.0	123.8	0	75	25
4	25 °C	1 h	86	11.3	2.0	117.0	0	75	25
5	0 °C	1 h	5	0.2	3.6	6.8	85	7	8
6	50 °C	10 min	90	6.6	2.3	734.4	0	75	25
7	50 °C	2 h	>99	23.9	2.9	68.0	0	75	25

^a^ Polymerization in toluene (5 mL), [Ip] = 4.0 mol/L, [**V3**]/[Ip]/[MAO] = 1/2000/100. ^b^ Determined by isolated yield. ^c^ Determined by gel permeation chromatography (GPC). ^d^ kg polymer (mol V)^−1^ h^−1^. ^e^ Determined by ^1^H NMR and ^13^C NMR. ^f^ [**V3**]/[Ip]/[MAO] = 1/2000/50. ^g^ [**V3**]/[Ip]/[MAO] = 1/2000/10.

## Supplementary Materials

The following are available online at https://www.mdpi.com/2073-4360/11/7/1122/s1, Table S1: Effects of cocatalyst and solvent on the isoprene polymerization with VCl_3_ catalyst, NMR spectra of the representative polyisoprene and GPC curves of polyisoprene samples.
