# Supplementary material for: Isoprene Polymerization: Catalytic Performance of Iminopyridine Vanadium(III) Chloride versus Vanadium(III) Chloride
Source: Polymers (Basel). 2019 Jul 2;11(7):1122. doi: 10.3390/polym11071122 (PMC6681085; doi:10.3390/polym11071122)
Supplement: Supplementary file 1 [file polymers-11-01122-s001.pdf]

Supporting Information

Isoprene Polymerization: Catalytic Performance of Iminopyridine Vanadium(III) Chloride *versus* Vanadium(III) Chloride

Mengmeng Zhao,[1‡] Qaiser Mahmood,[1‡] Chuyang Jing,[1,2] Liang Wang,[1] Guangqian Zhu,[1,2] Xianhui Zhang,[1,2] and Qinggang Wang[1,2]*

[1] *Key Laboratory of Biobased Materials, Qingdao Institute of Biomass Energy and Bioprocess Technology, Chinese Academy of Sciences, Qingdao 266000, China*

[2] *Center of Materials Science and Optoelectronics Engineering，University of Chinese Academy of Sciences, Beijing 100049, China.*

‡ *These authors contributed equally to this work.*

* *Correspondence: wangqg@qibebt.ac.cn.*

Table S1……………………..…………………………………………..…………..….2

ATR-IR spectra of vanadium ligands and complexes……………………………………3–6

NMR spectra of resulting polyisoprenes for all entries……..………….……….……….7–32

GPC data of resulting polyisoprenes for all entries……………...……………………..…33–45

Table S1. Effects of cocatalyst and solvent on the isoprene polymerization with VCl_3 catalyst[a]

Entry	Cocat.	Sol.	Yield (%)[b]	*trans*-1,4 (%)[c]	*cis*-1,4 (%)[c]	3,4 (%)[c]	M_n[d] (×10^{-4})	M_w/M_n[d]
1	MAO	Toluene	65	83	14	3	11.5	2.3
2	$AlEt_3$	Toluene	9	98	0	2	9.7	1.8
3	Al(*i*-Bu)$_3$	Toluene	38	>99	0	0	8.4	2.8
4	MAO	DCM	68	--	--	--	--	--
5	MAO	Hexane	37	70	23	7	12.0	1.8

[a] General condition: $[Ip]_0$ = 5.0 M; [Ip]/[Al]/[V] = 200/30/1; 50 ºC; 15 h; addition sequence: VCl_3, toluene, isoprene, methylaluminoxane (MAO).

[b] Determined by isolated yield.

[c] Determined by ^1H NMR and ^{13}C NMR.

[d] Determined by gel permeation chromatography (GPC)

ATR-IR spectra of vanadium complexes

ATR-IR spectra of Ligand 1

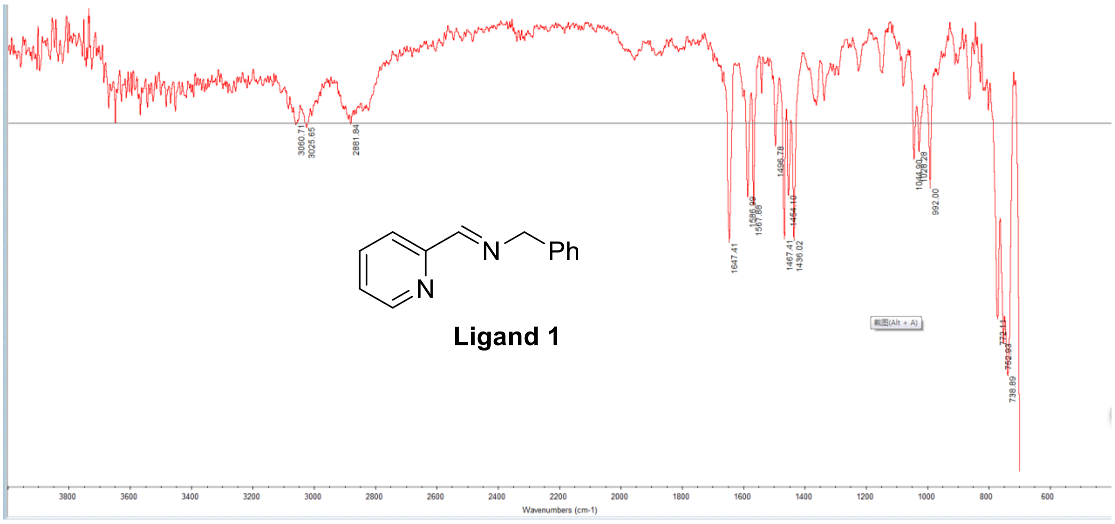

ATR-IR spectra of V1

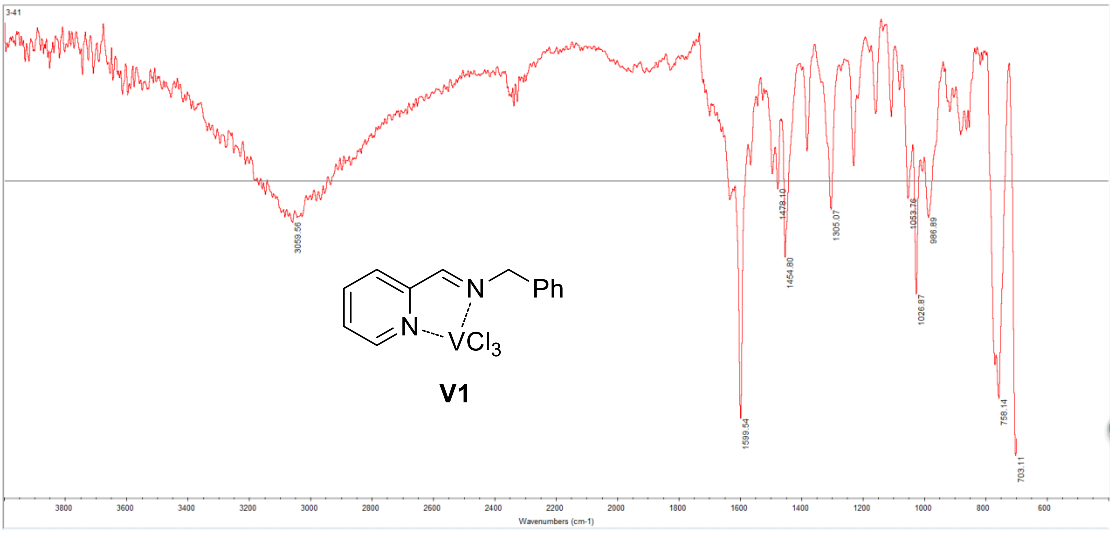

ATR-IR spectra of Ligand 2

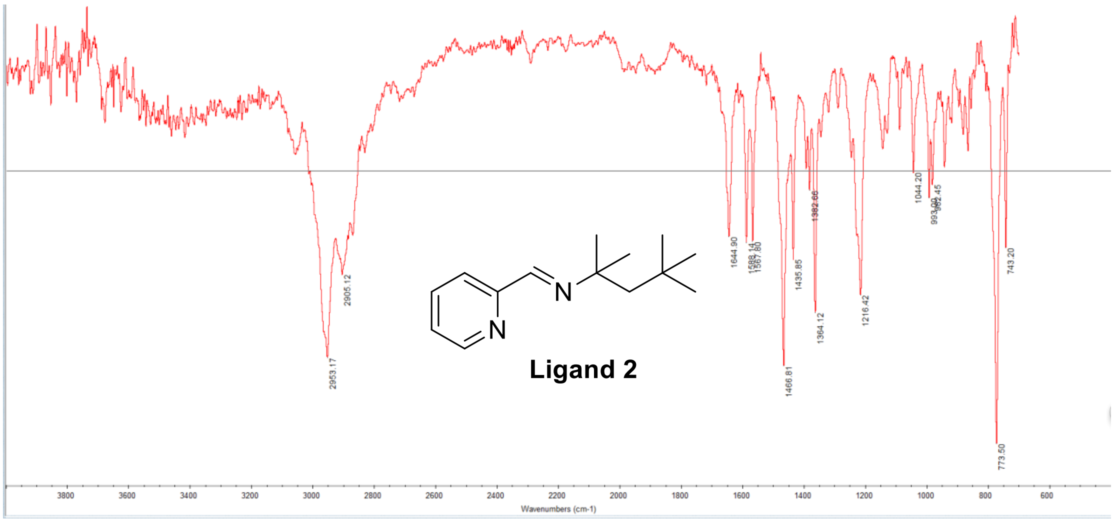

ATR-IR spectra of V2

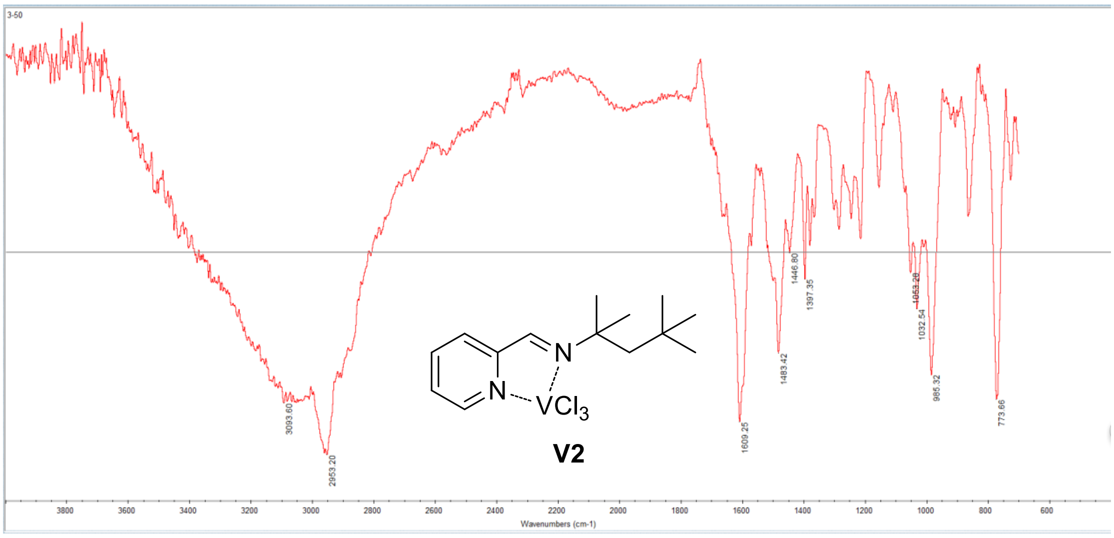

ATR-IR spectra of Ligand 3

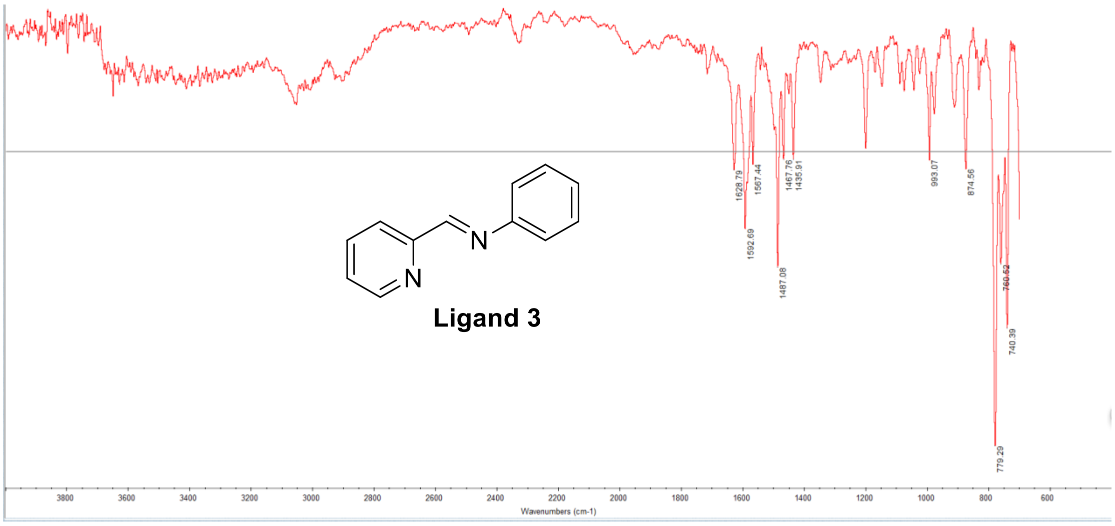

ATR-IR spectra of V3

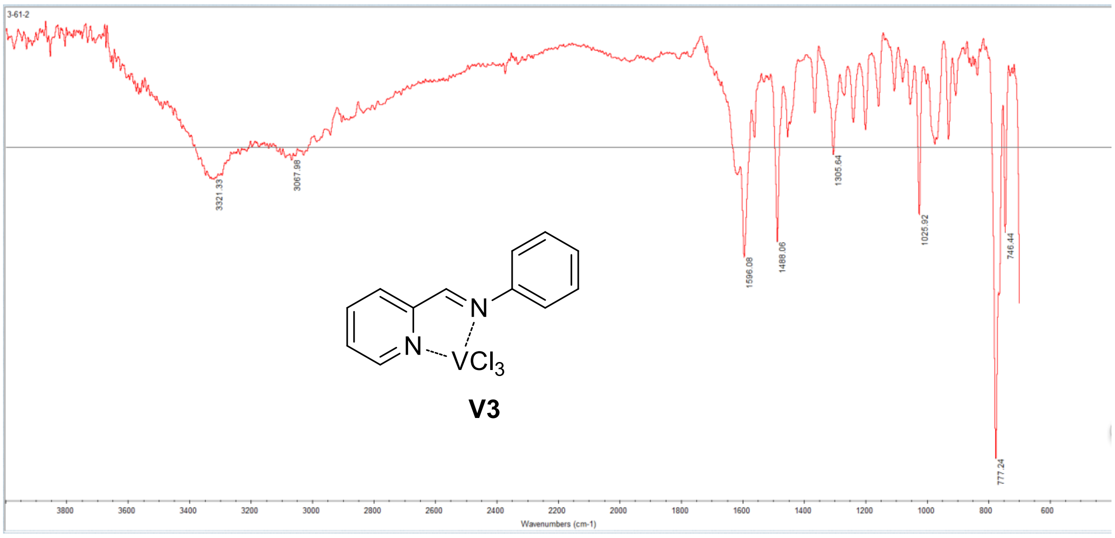

ATR-IR spectra of Ligand 4

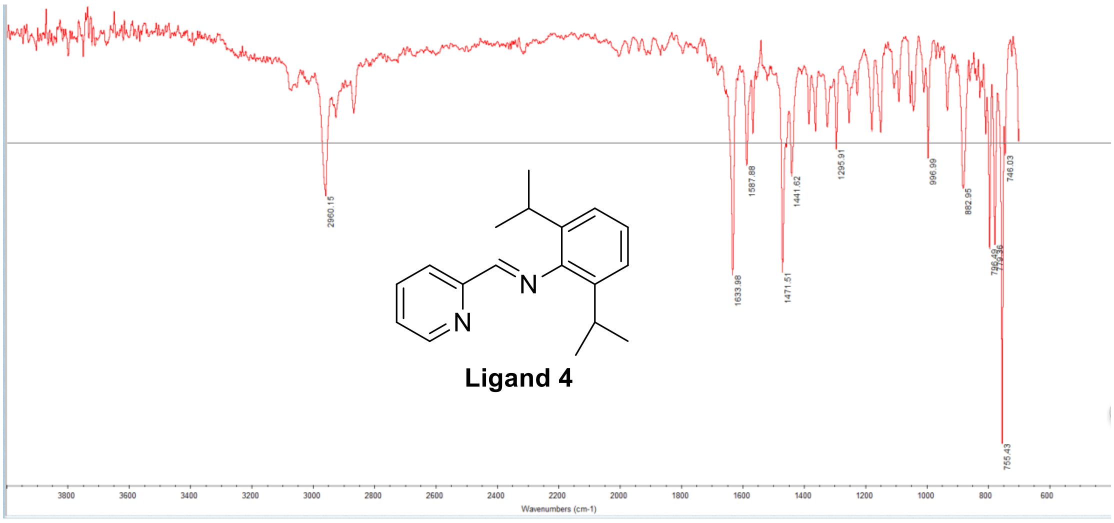

ATR-IR spectra of V4

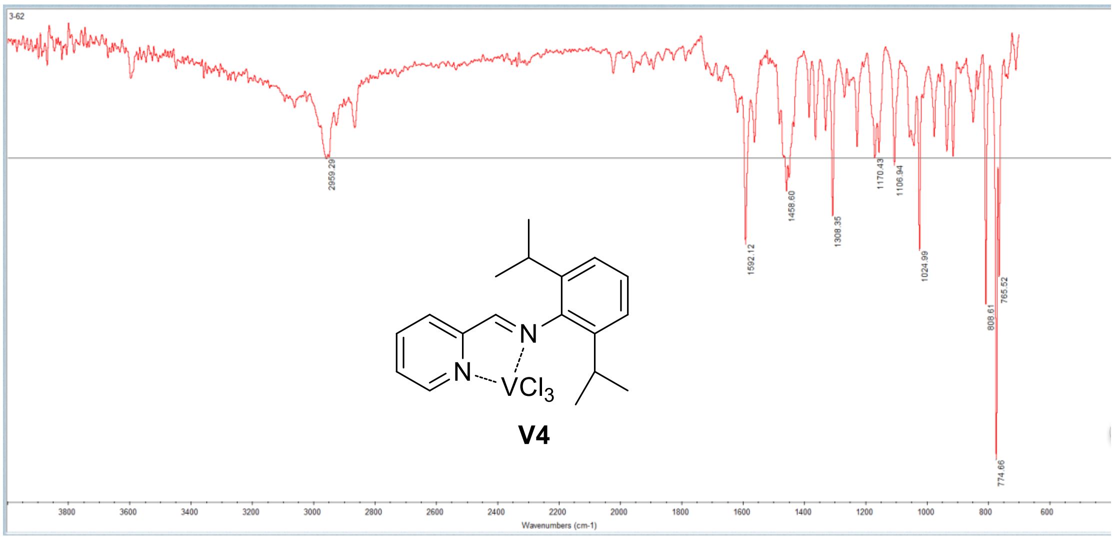

Table S1, entry 1 (83% *trans*-1,4; 14% *cis*-1,4; 3% 3,4)

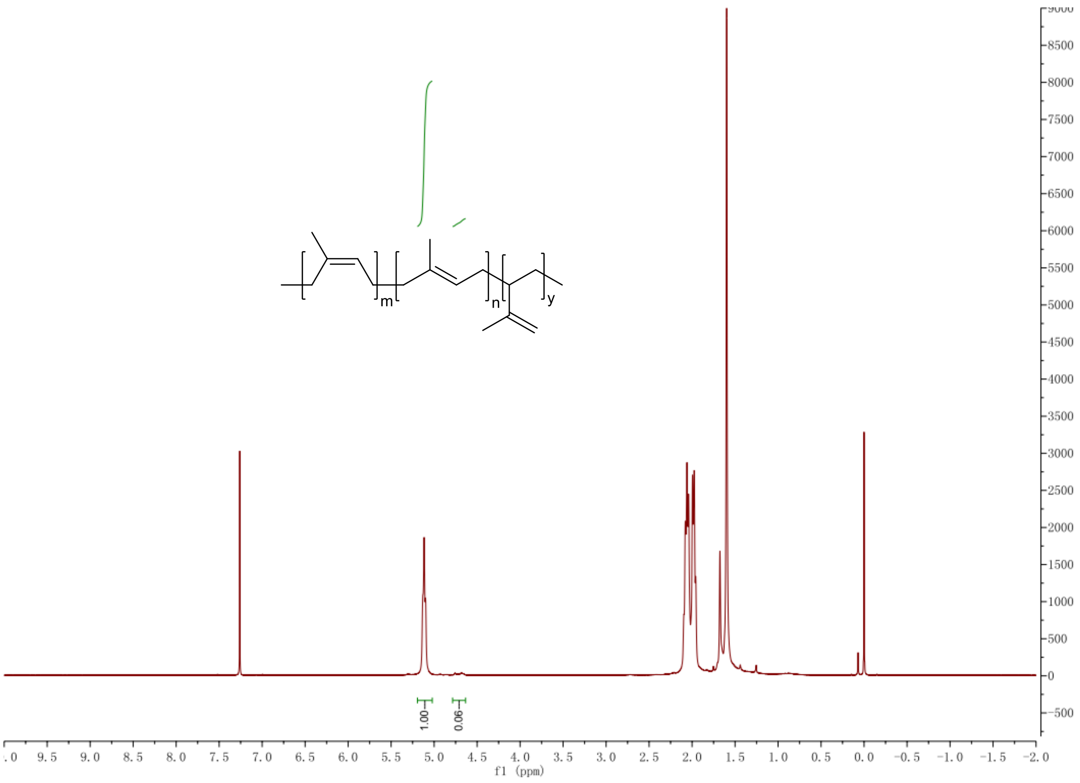

^{1}H NMR spectrum (400 MHz, $CDCl_3$, 25 ^{o}C)

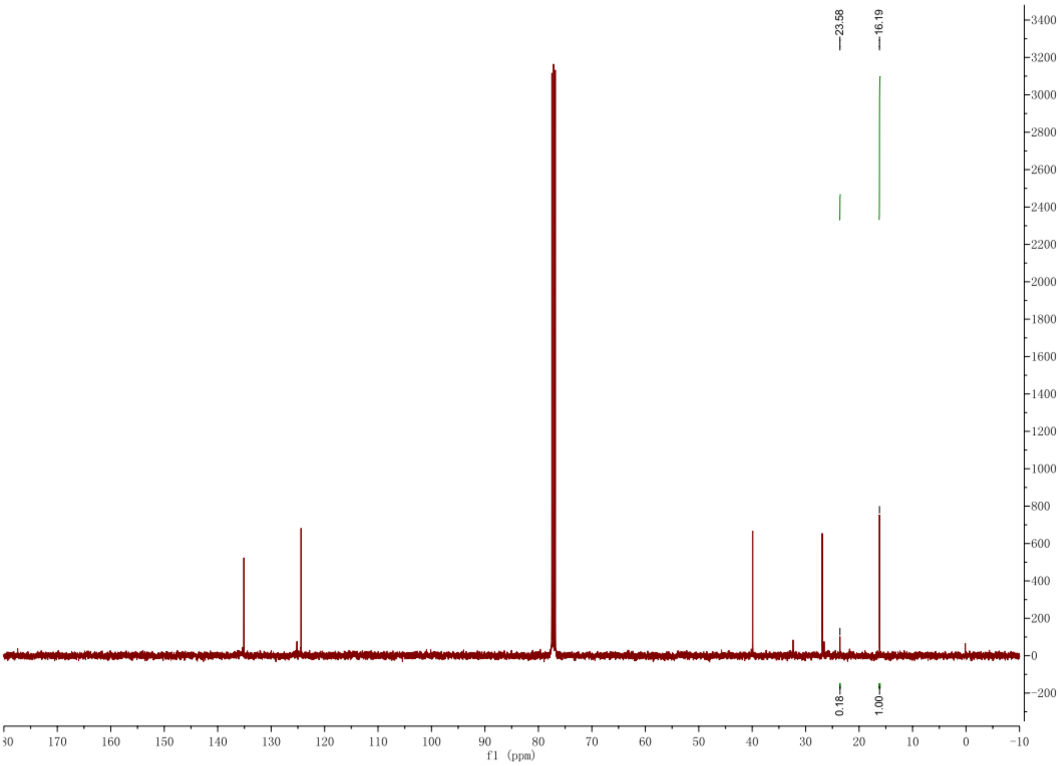

^{13}C NMR spectrum (100 MHz, $CDCl_3$, 25 ^{o}C)

Table S1, entry 2 (98% *trans*-1,4; 2% 3,4)

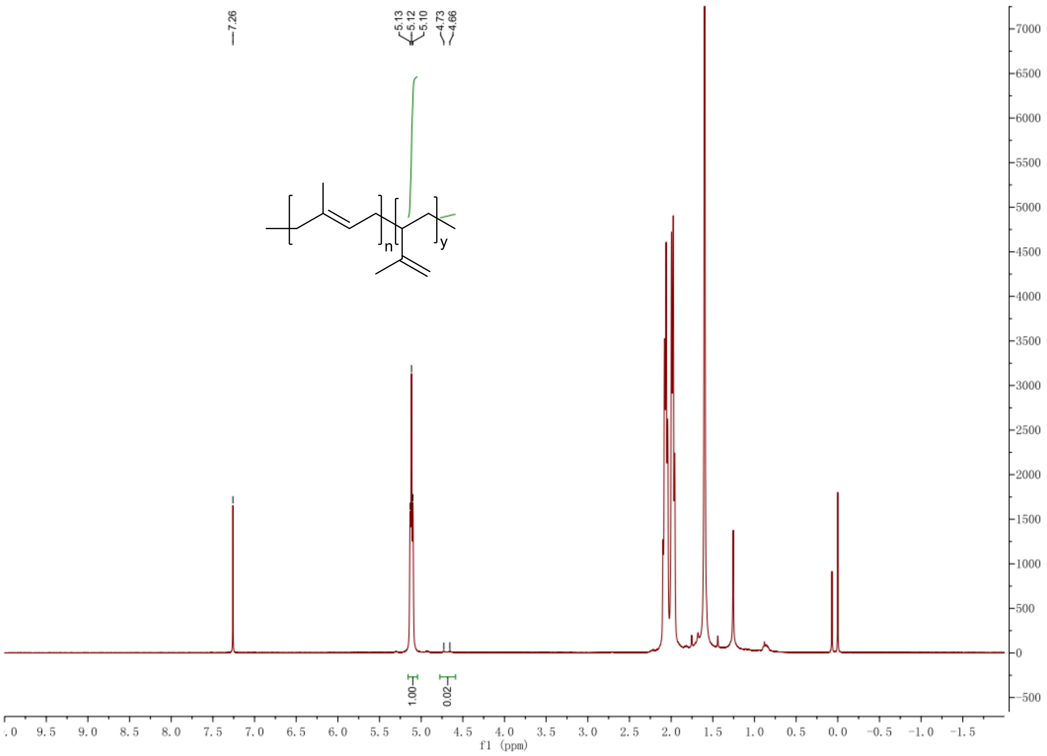

^1H NMR spectrum (400 MHz, $CDCl_3$, 25 °C)

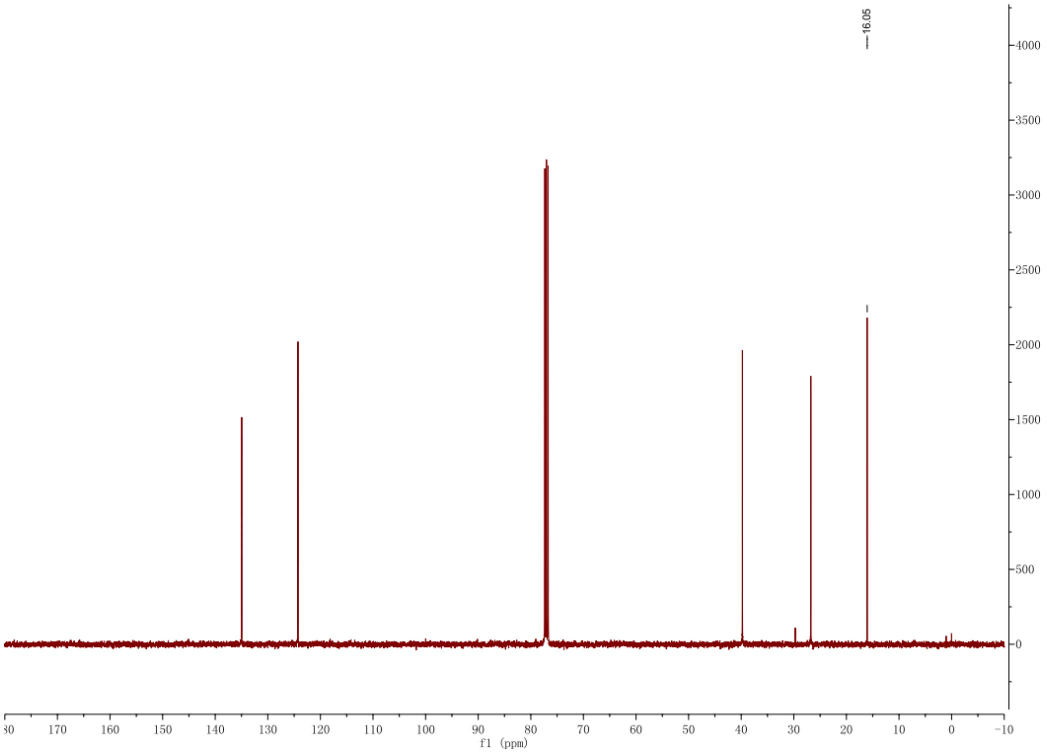

^{13}C NMR spectrum (100 MHz, $CDCl_3$, 25 °C)

Table S1, entry 3 (>98% *trans*-1,4)

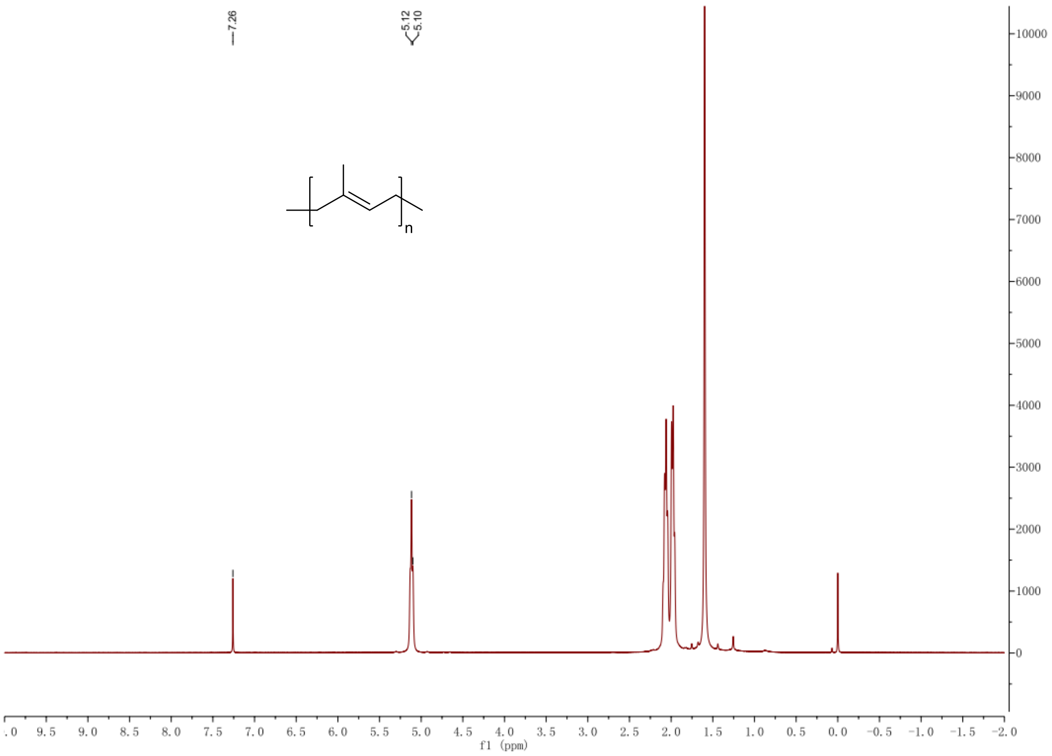

^1H NMR spectrum (400 MHz, $CDCl_3$, 25 °C)

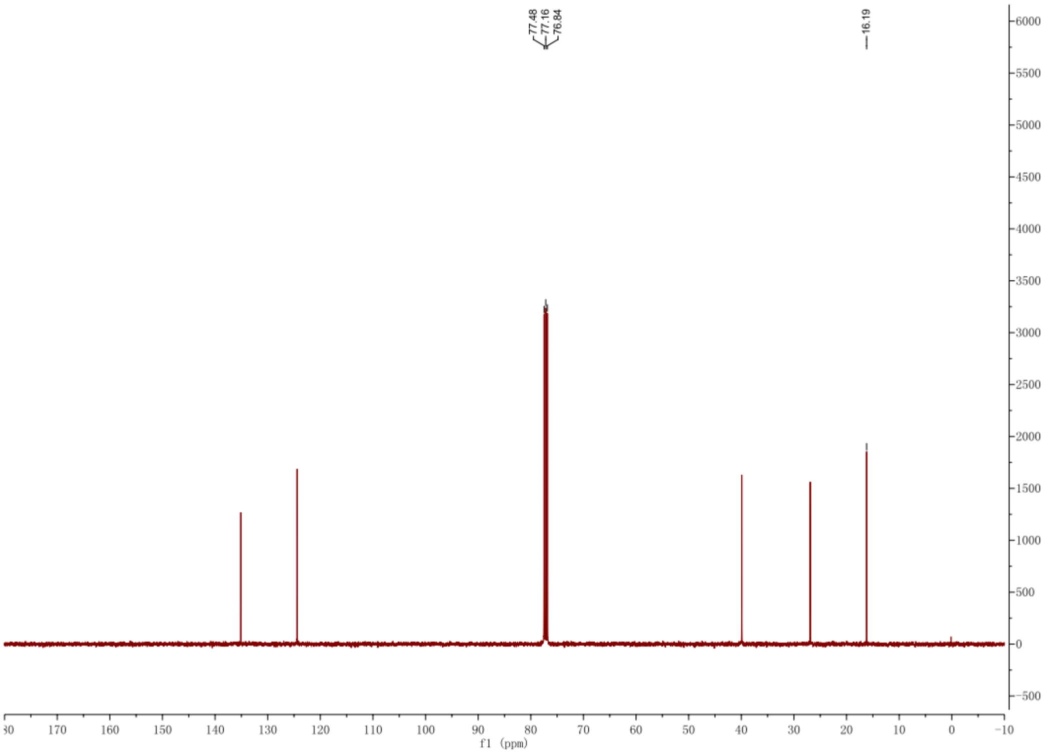

^{13}C NMR spectrum (100 MHz, $CDCl_3$, 25 °C)

Table S1, entry 4

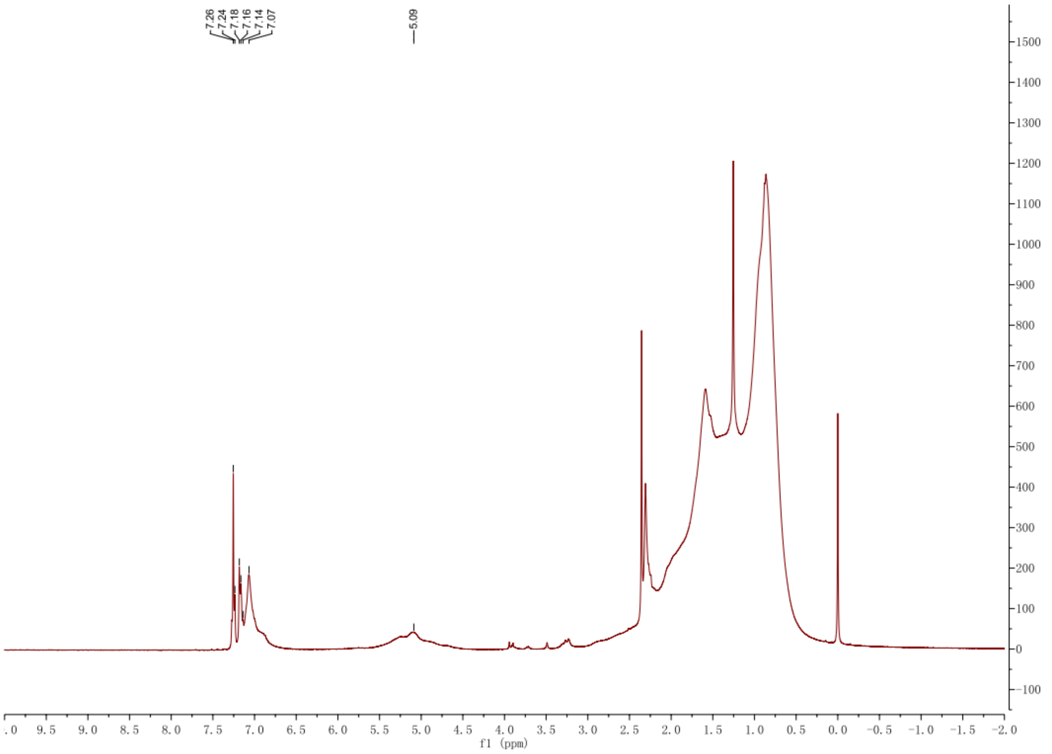

^{1}H NMR spectrum (400 MHz, $CDCl_3$, 25 °C)

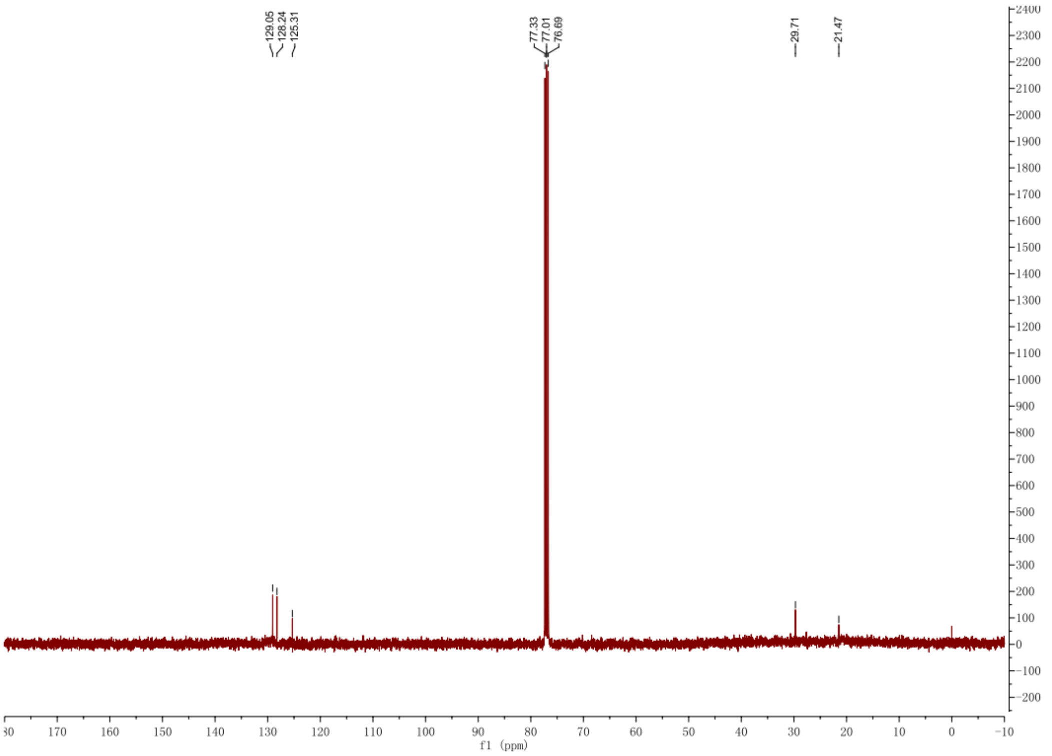

^{13}C NMR spectrum (100 MHz, $CDCl_3$, 25 °C)

Table S1, entry 5 (70% *trans*-1,4; 23% *cis*-1,4; 7% 3,4)

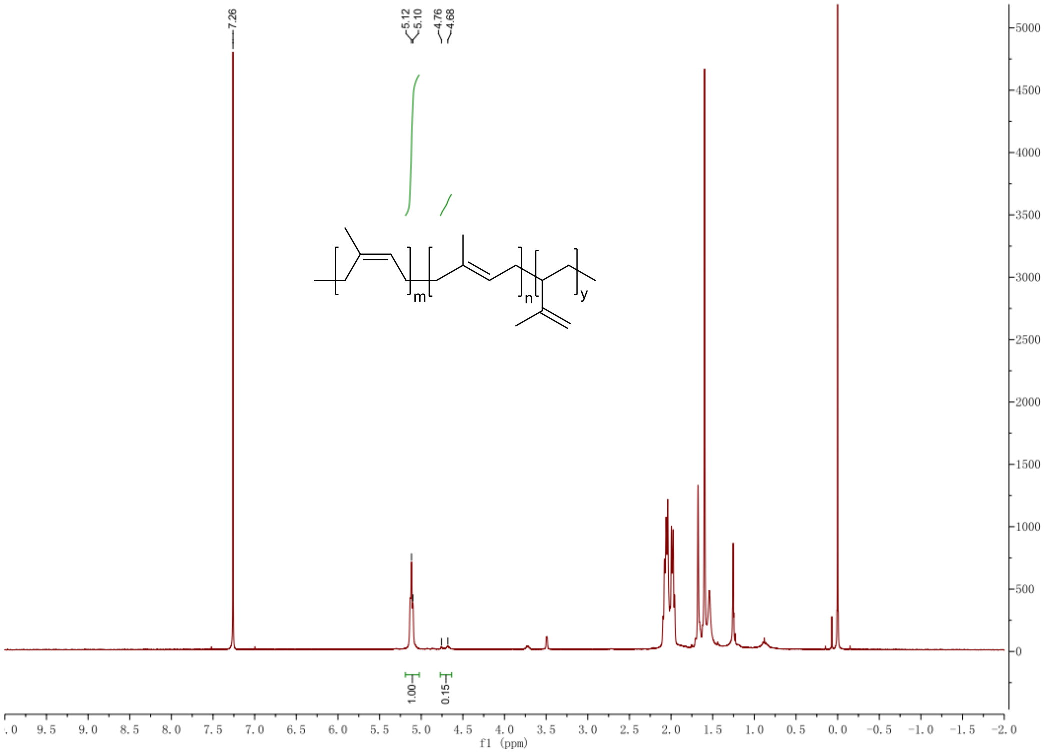

^{1}H NMR spectrum (400 MHz, $CDCl_3$, 25 ^{o}C)

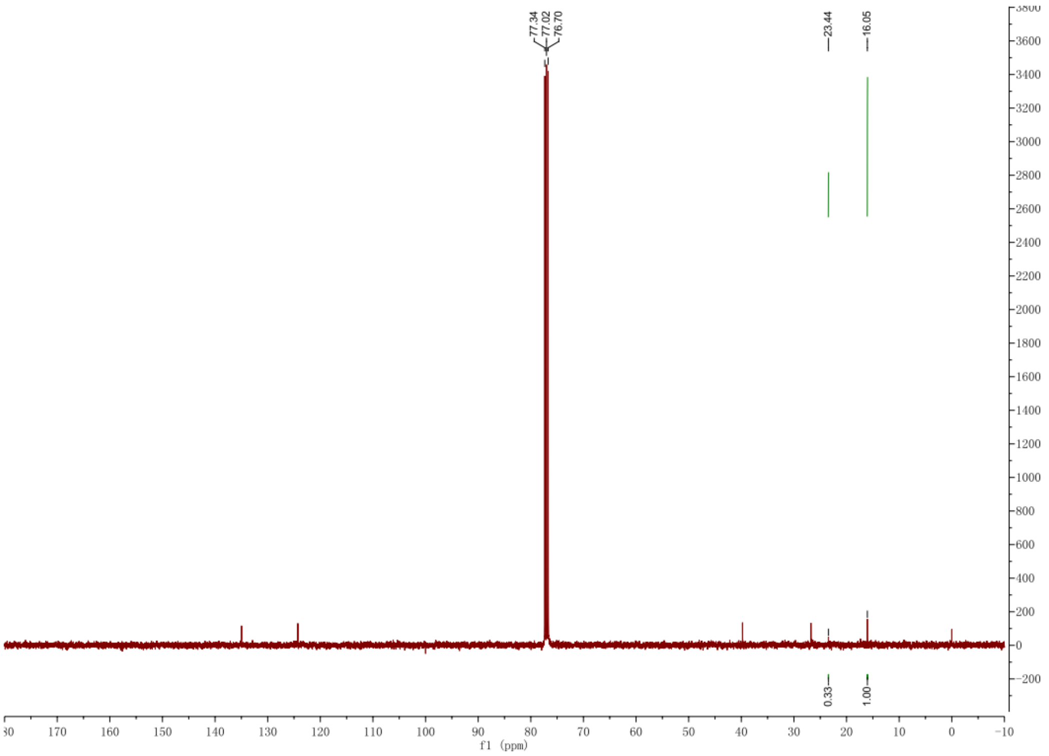

^{13}C NMR spectrum (100 MHz, $CDCl_3$, 25 ^{o}C)

Table 1, entry 1 (83% *trans*-1,4; 14% *cis*-1,4; 3% 3,4)

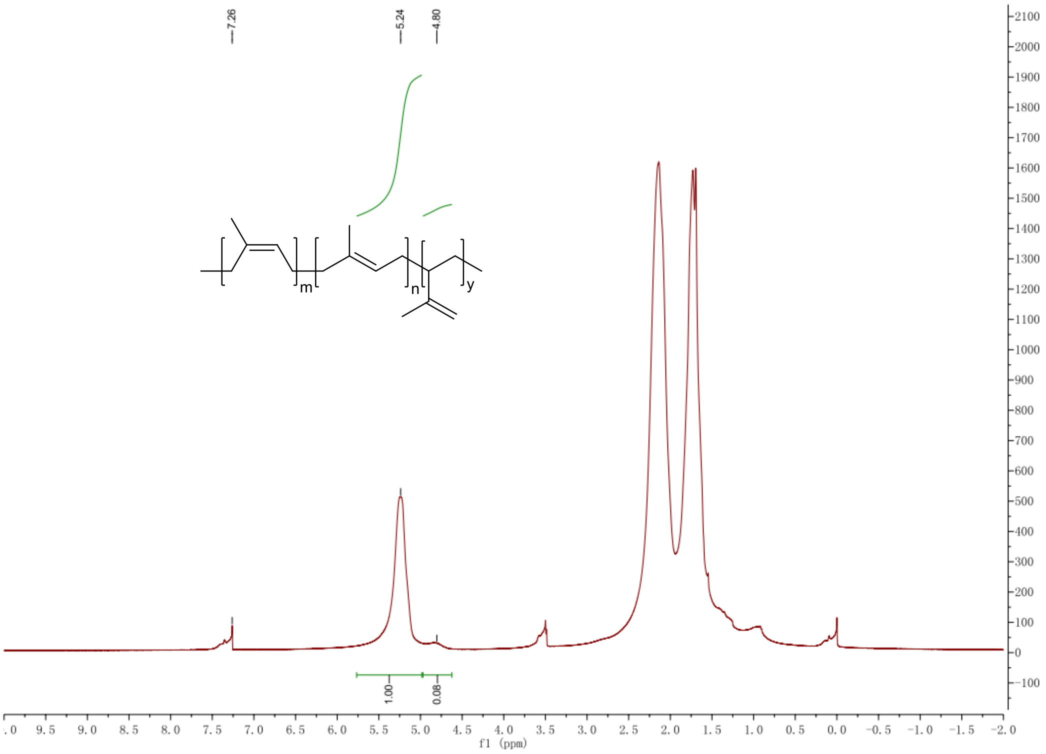

^{1}H NMR spectrum (400 MHz, $CDCl_3$, 25 °C)

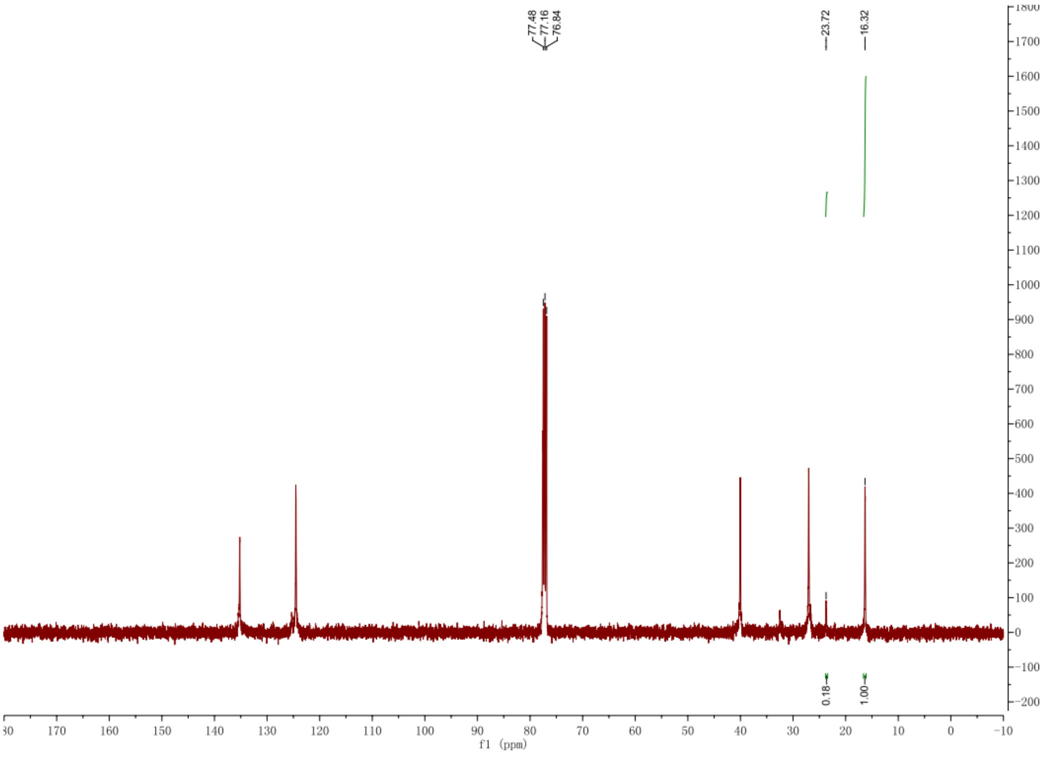

^{13}C NMR spectrum (100 MHz, $CDCl_3$, 25 °C)

Table 1, entry 2 (68% *trans*-1,4; 25% *cis*-1,4; 7% 3,4)

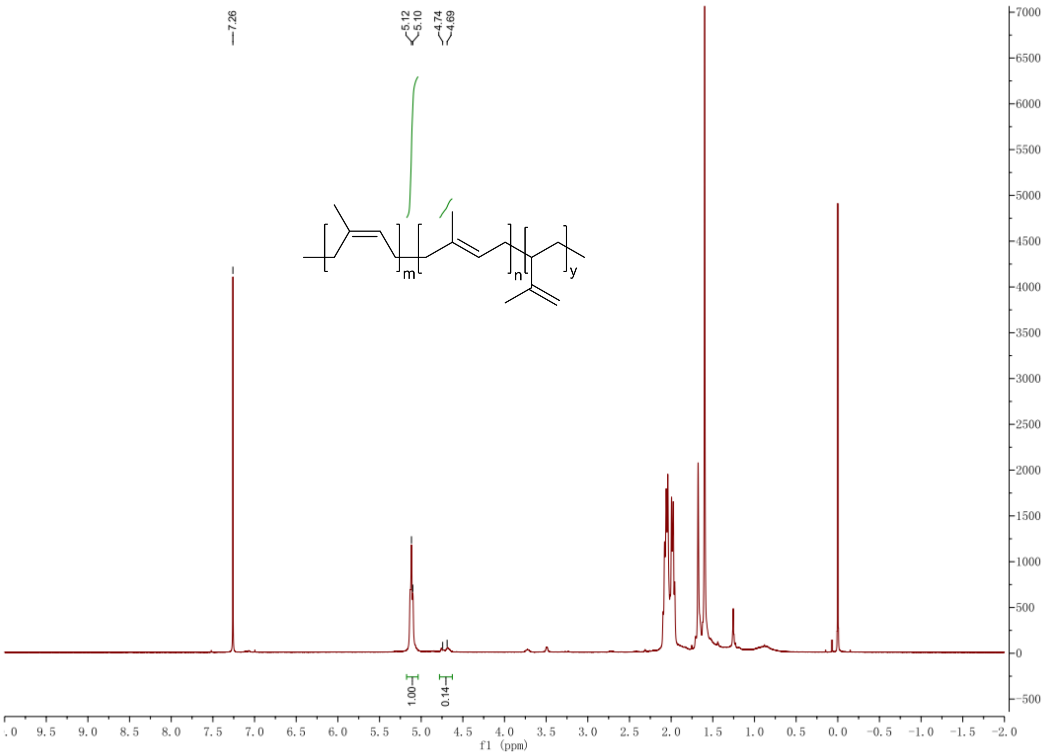

^{1}H NMR spectrum (400 MHz, $CDCl_3$, 25 ^{o}C)

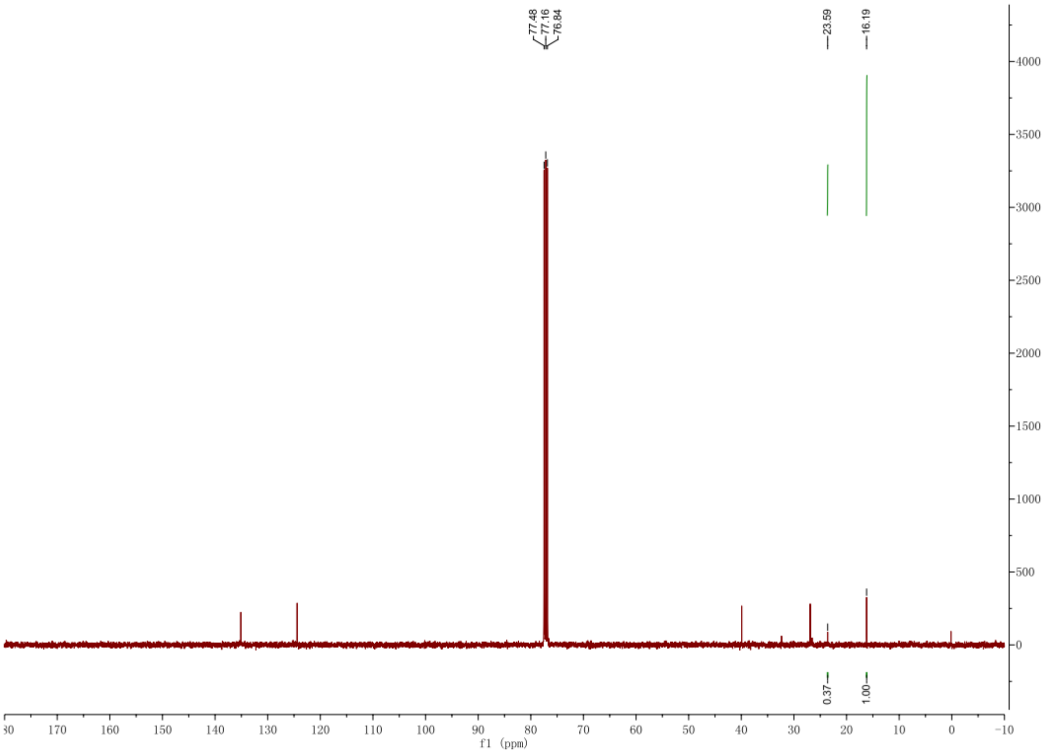

^{13}C NMR spectrum (100 MHz, $CDCl_3$, 25 ^{o}C)

Table 1, entry 3 (>99% *trans*-1,4)

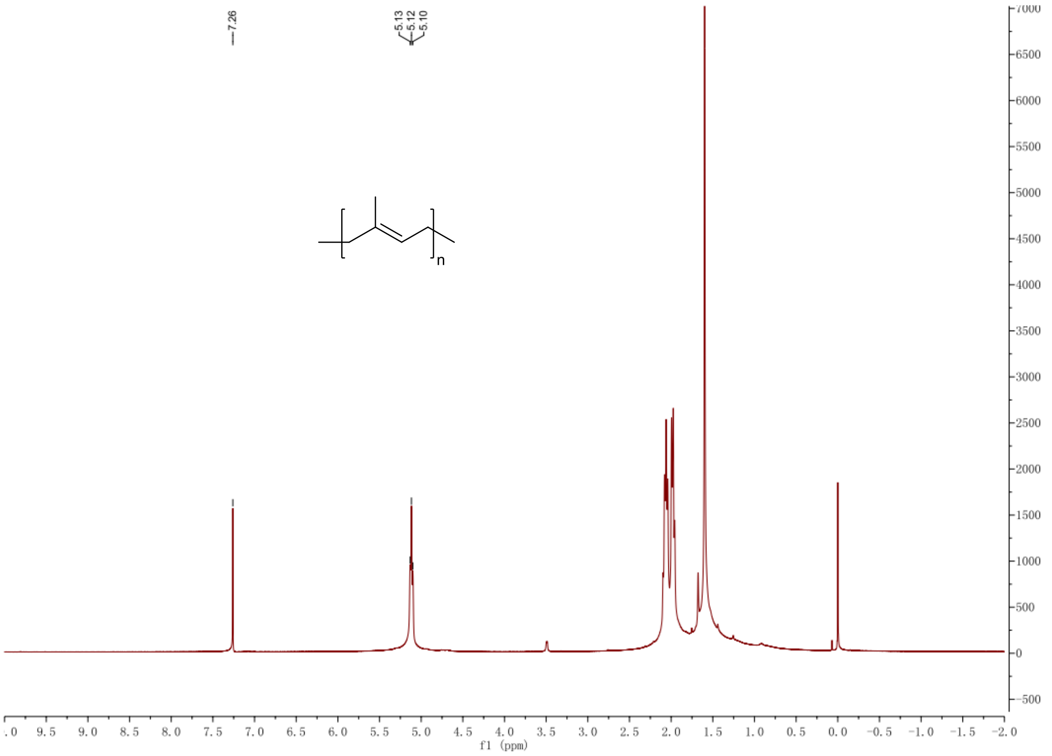

^{1}H NMR spectrum (400 MHz, $CDCl_3$, 25 ^{o}C)

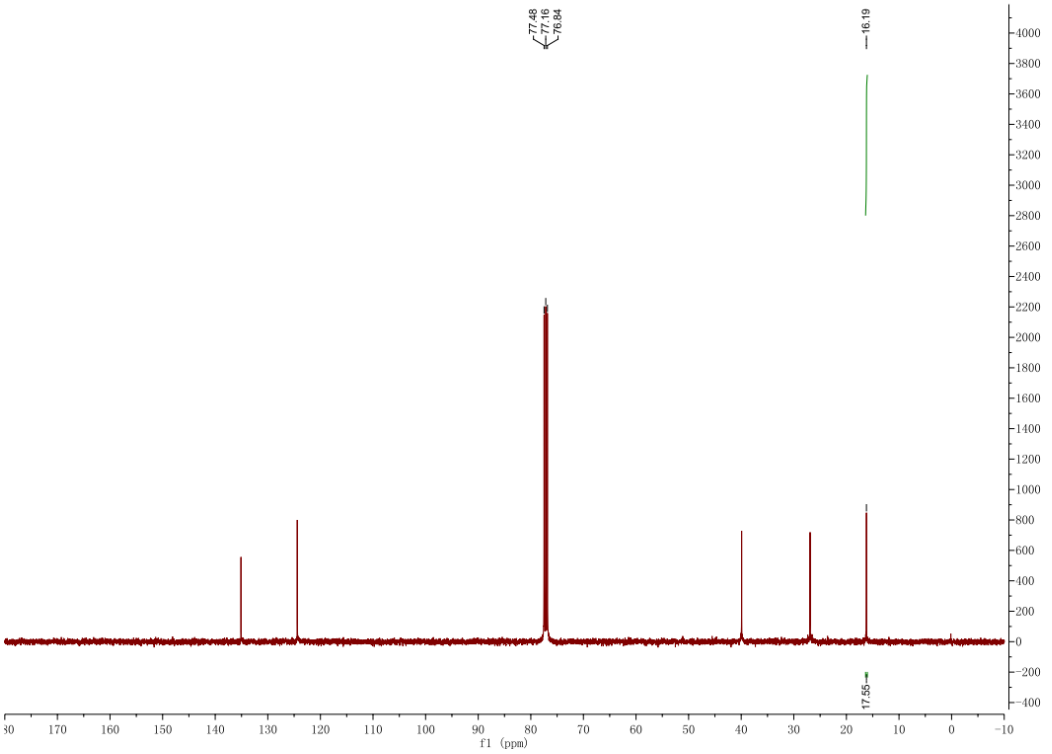

^{13}C NMR spectrum (100 MHz, $CDCl_3$, 25 ^{o}C)

Table 1, entry 4 (>99% *trans*-1,4)

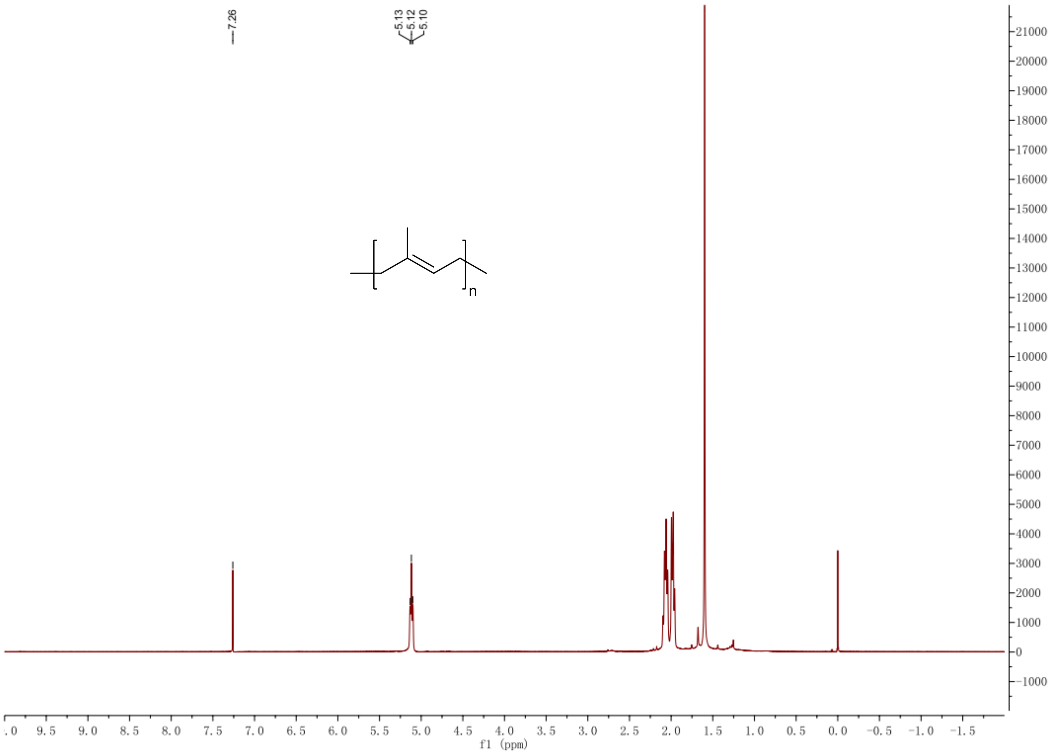

^{1}H NMR spectrum (400 MHz, $CDCl_3$, 25 °C)

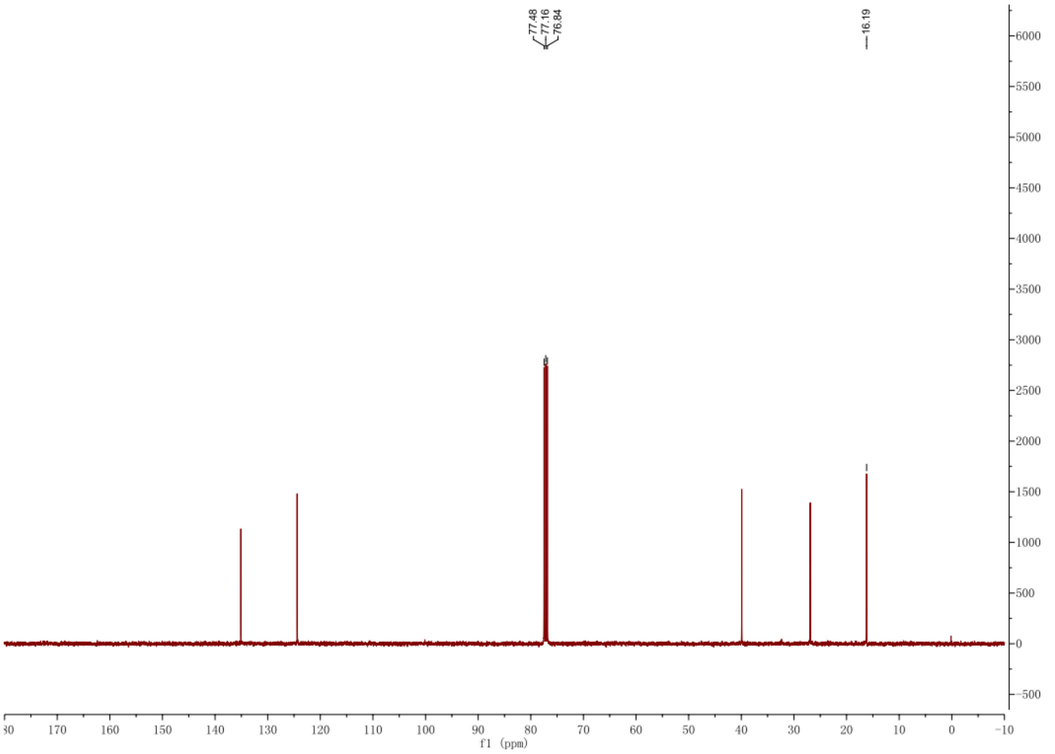

^{13}C NMR spectrum (100 MHz, $CDCl_3$, 25 °C)

Table 1, entry 5 (>99% *trans*-1,4)

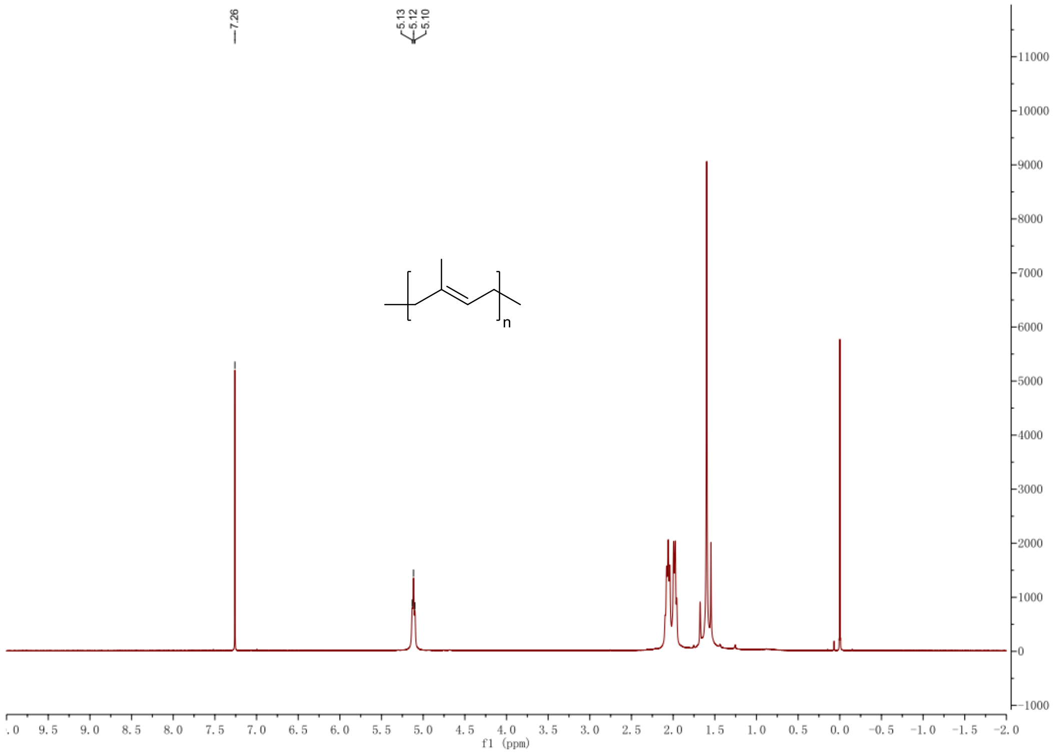

^{1}H NMR spectrum (400 MHz, $CDCl_3$, 25 °C)

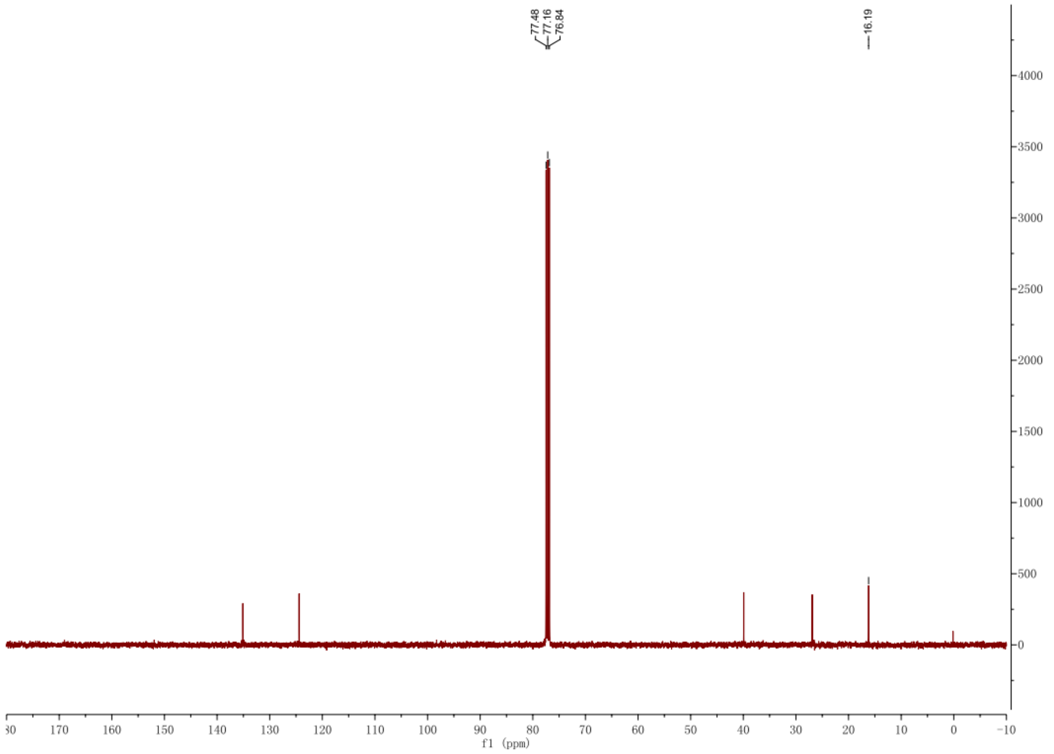

^{13}C NMR spectrum (100 MHz, $CDCl_3$, 25 °C)

Table 1, entry 6 (>99% *trans*-1,4)

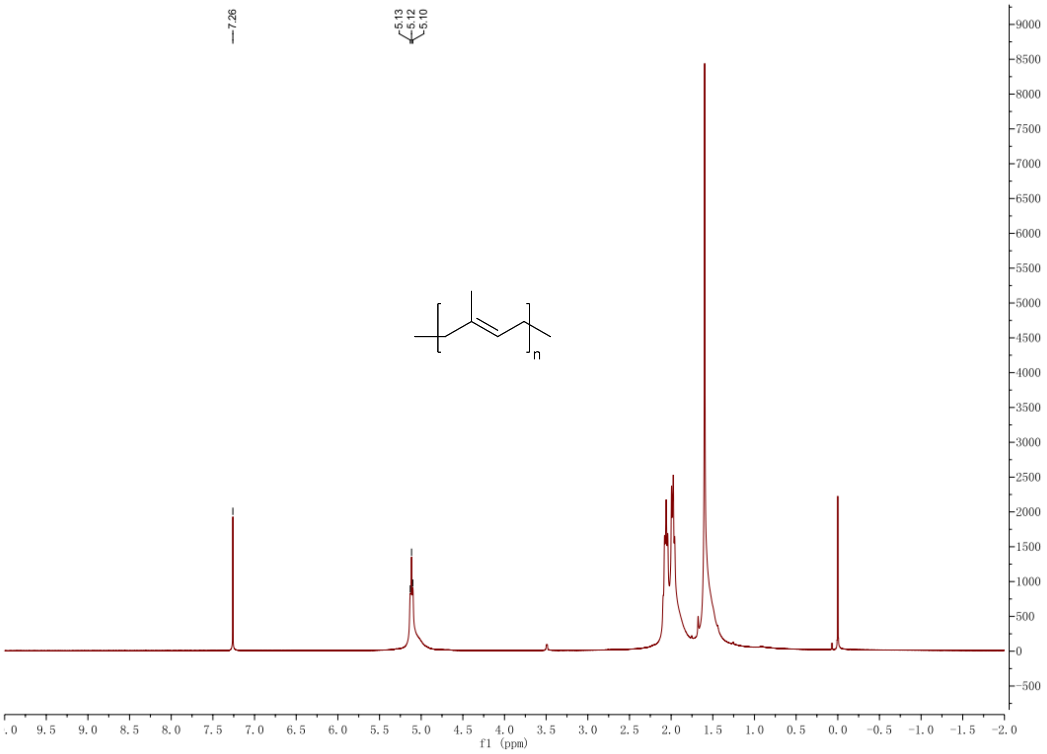

^{1}H NMR spectrum (400 MHz, $CDCl_3$, 25 ^{o}C)

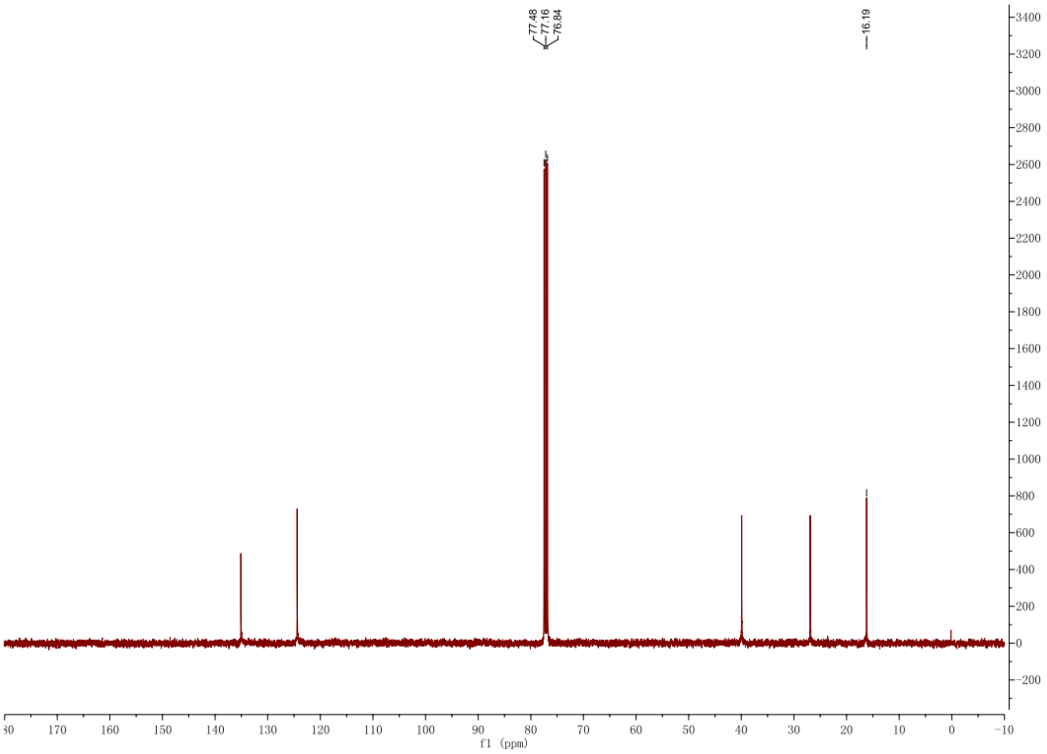

^{13}C NMR spectrum (100 MHz, $CDCl_3$, 25 ^{o}C)

Table 1, entry 7 (>99% *trans*-1,4)

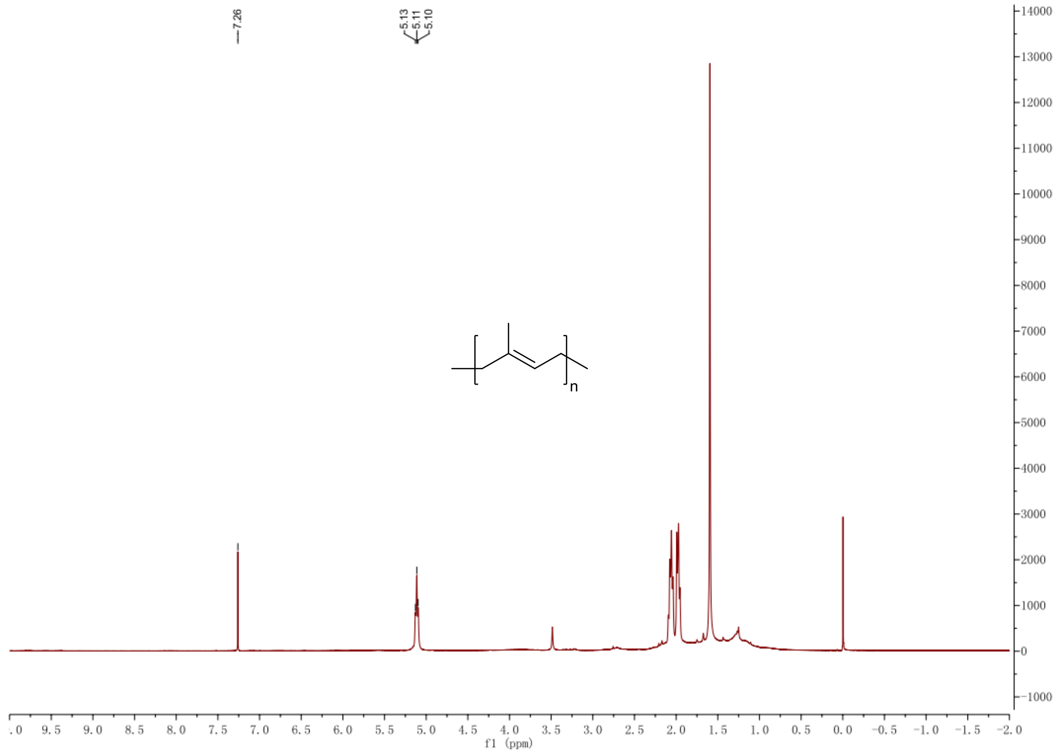

^{1}H NMR spectrum (400 MHz, $CDCl_3$, 25 °C)

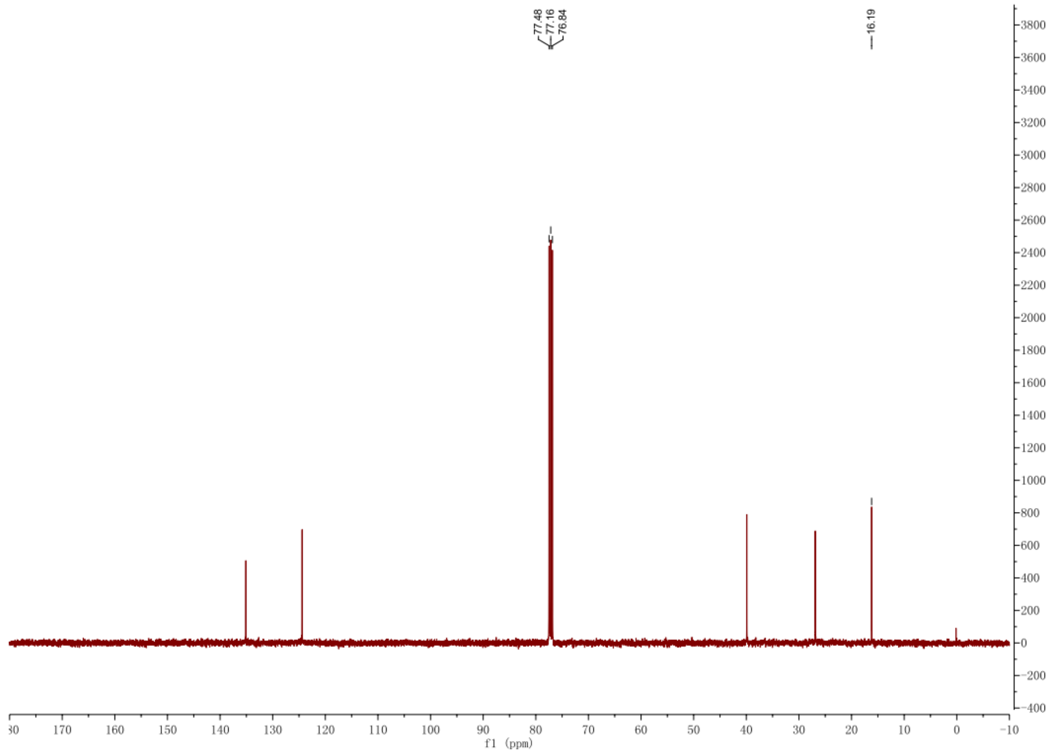

^{13}C NMR spectrum (100 MHz, $CDCl_3$, 25 °C

Table 1, entry 8 (>99% *trans*-1,4)

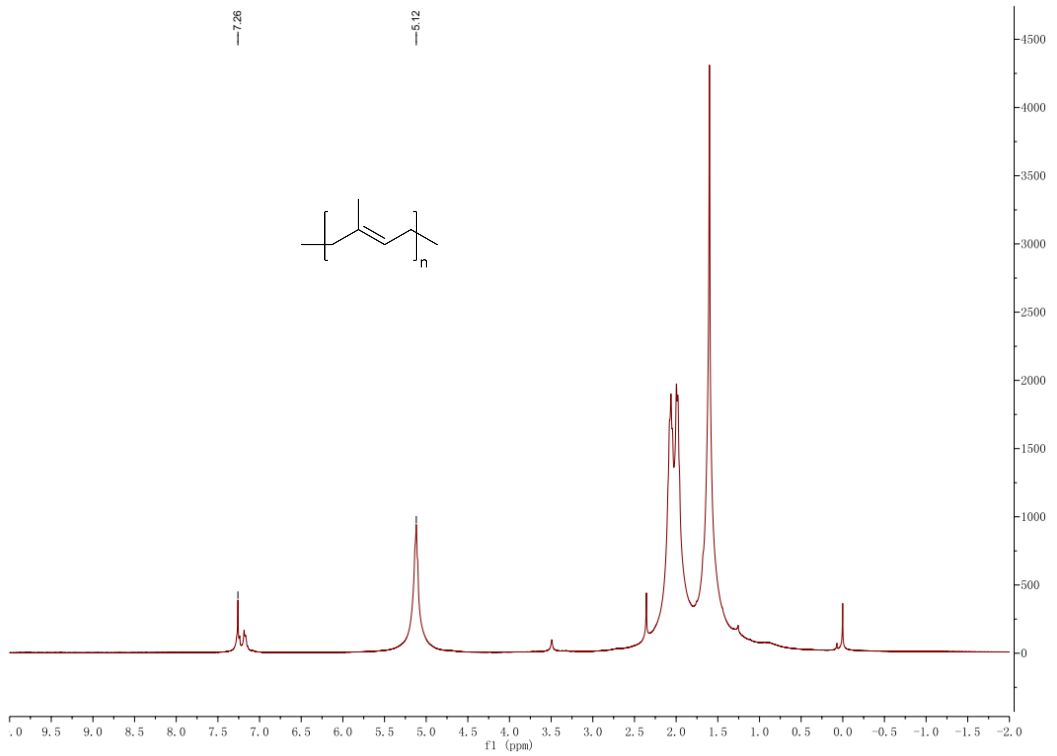

^{1}H NMR spectrum (400 MHz, $CDCl_3$, 25 ^{o}C)

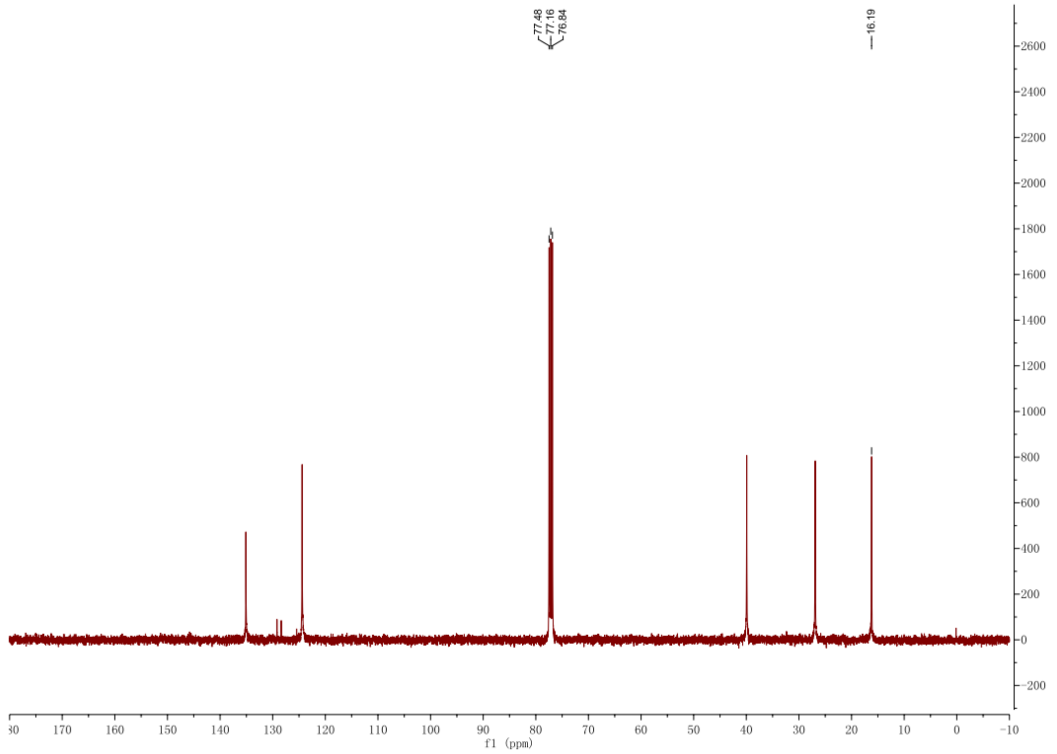

^{13}C NMR spectrum (100 MHz, $CDCl_3$, 25 ^{o}C)

Table 1, entry 9 (>99% *trans*-1,4)

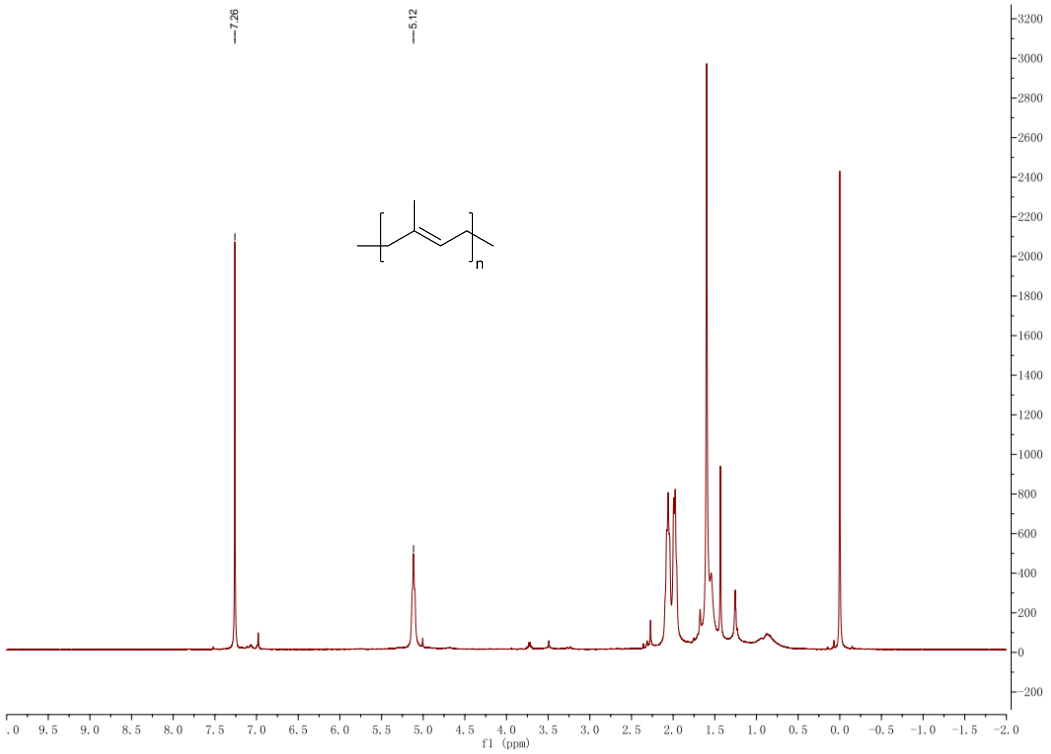

^{1}H NMR spectrum (400 MHz, $CDCl_3$, 25 °C)

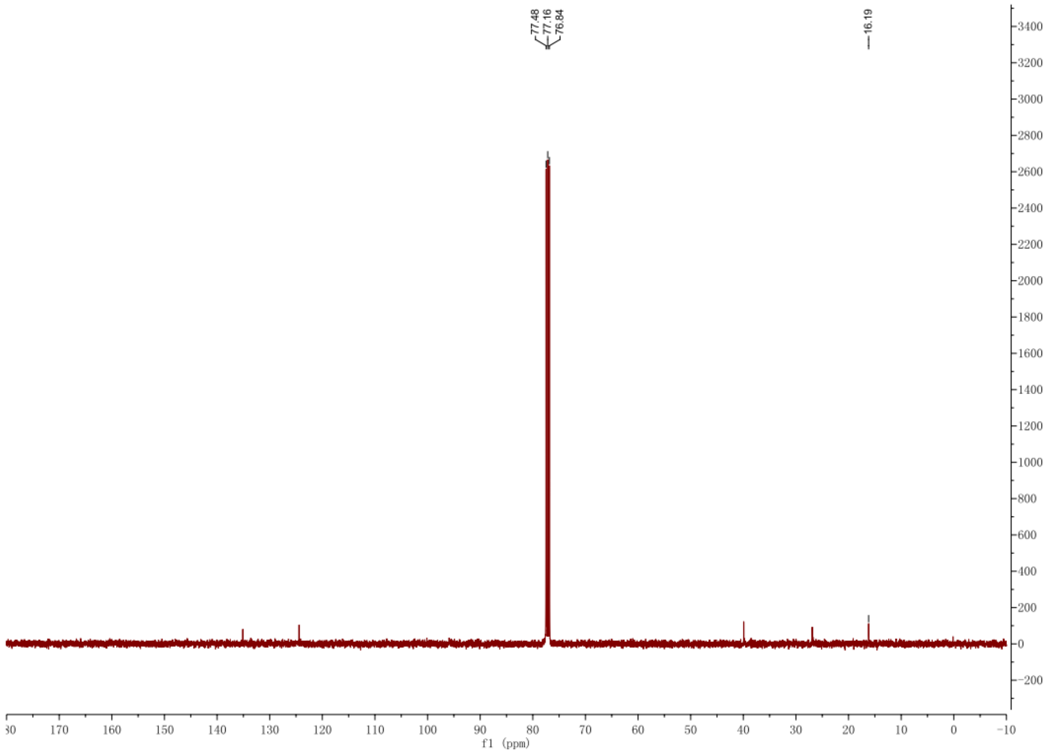

^{13}C NMR spectrum (100 MHz, $CDCl_3$, 25 °C)

Table 1, entry 10 (>99% *trans*-1,4)

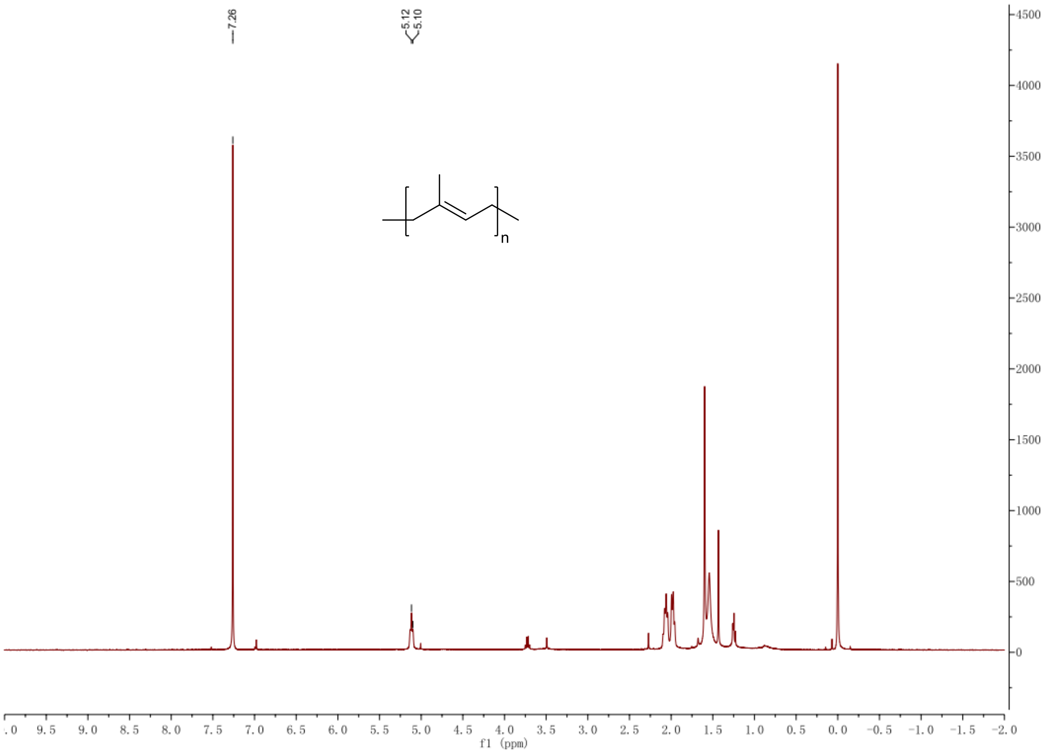

^{1}H NMR spectrum (400 MHz, $CDCl_3$, 25 ^{o}C)

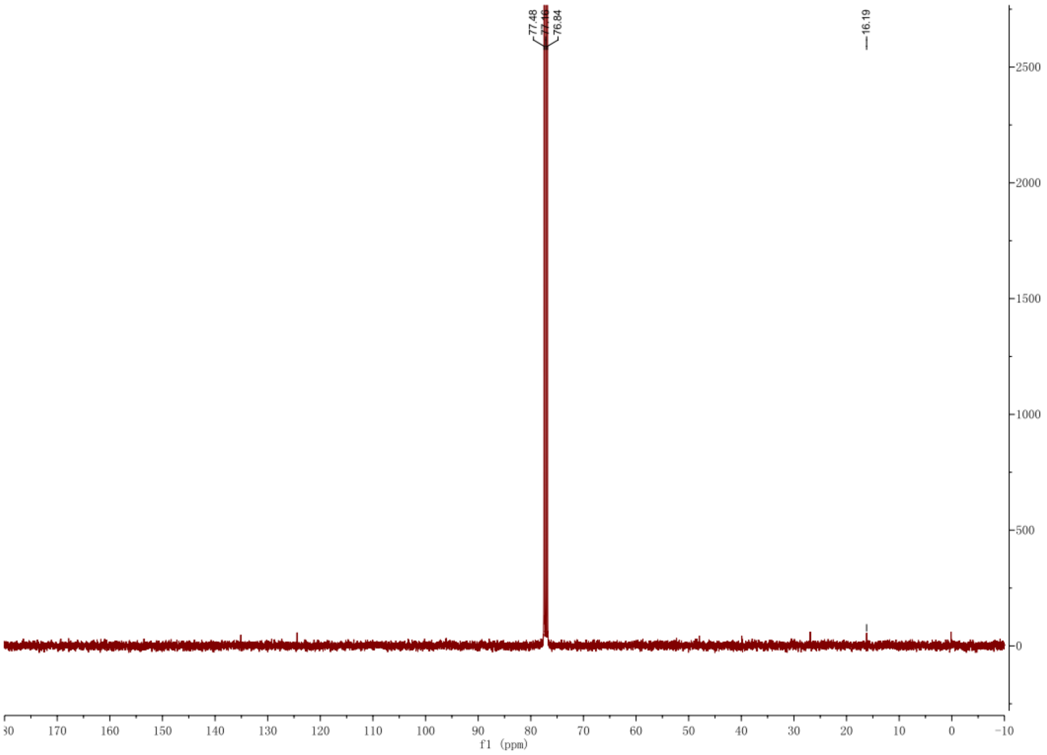

^{13}C NMR spectrum (100 MHz, $CDCl_3$, 25 ^{o}C)

Table 2, entry 1 (22% *trans*-1,4; 54% *cis*-1,4; 24% 3,4)

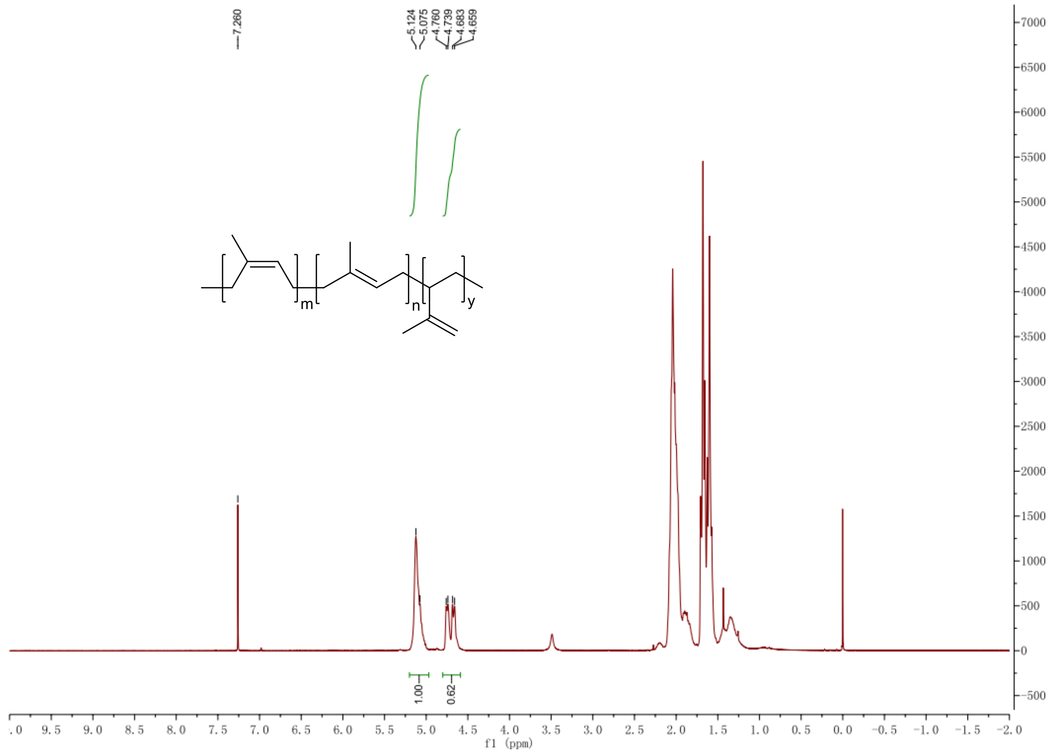

^{1}H NMR spectrum (400 MHz, $CDCl_3$, 25 ^{o}C)

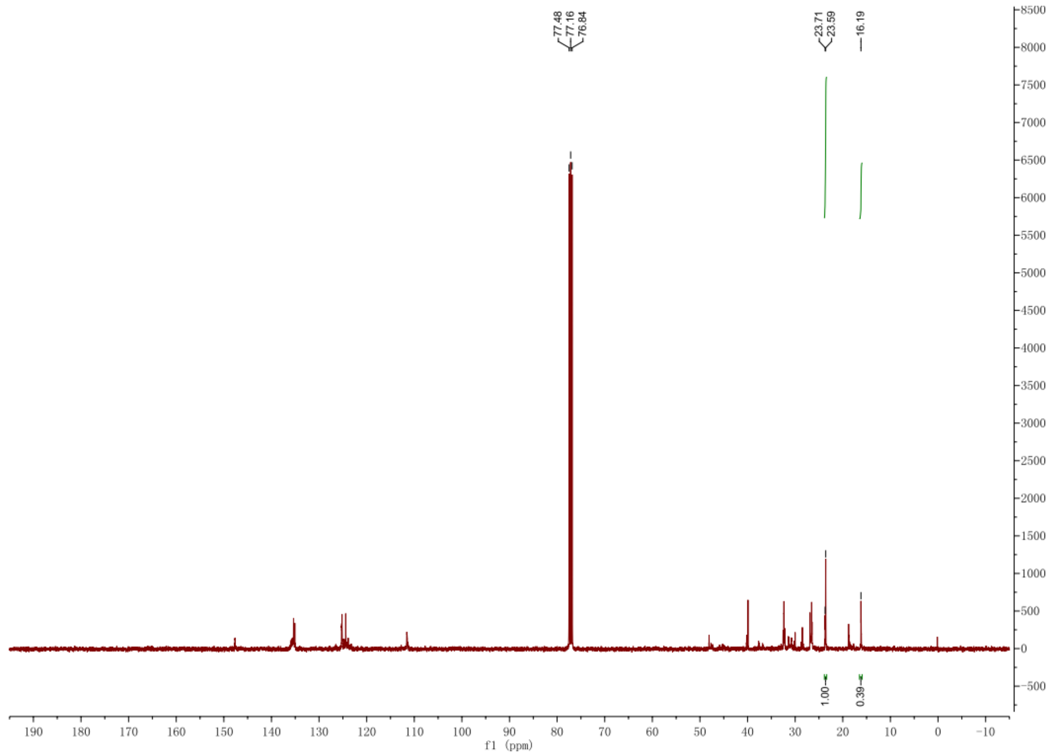

^{13}C NMR spectrum (100 MHz, $CDCl_3$, 25 ^{o}C)

Table 2, entry 2 (36% *trans*-1,4; 47% *cis*-1,4; 17% 3,4)

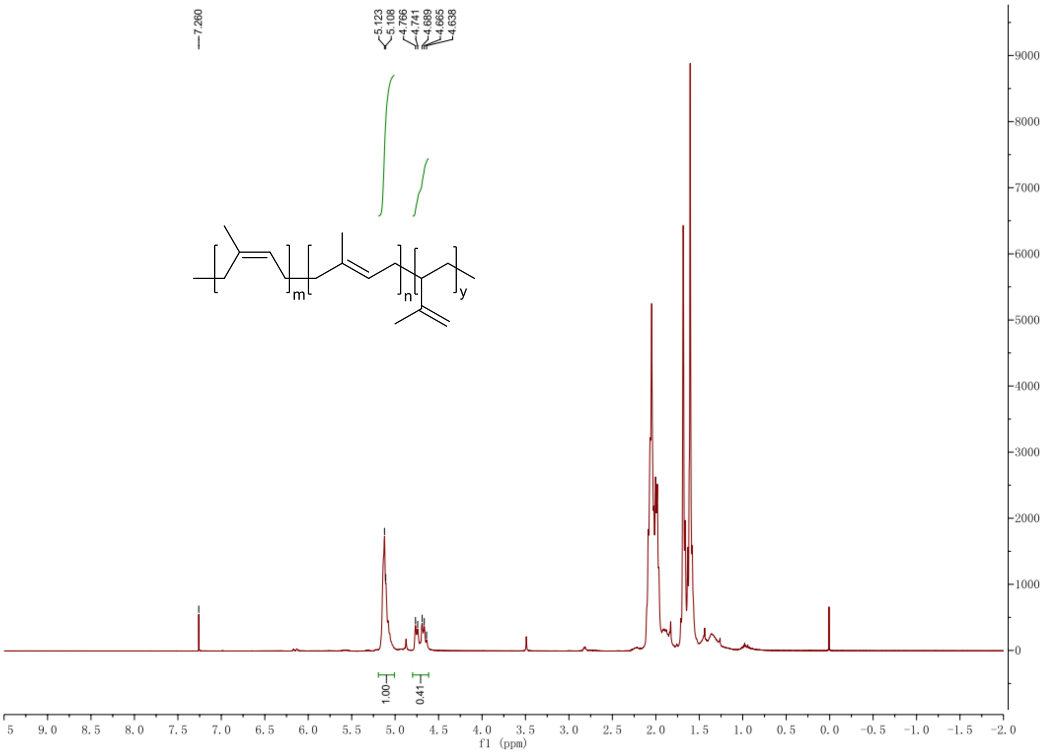

^{1}H NMR spectrum (400 MHz, $CDCl_3$, 25 ^{o}C)

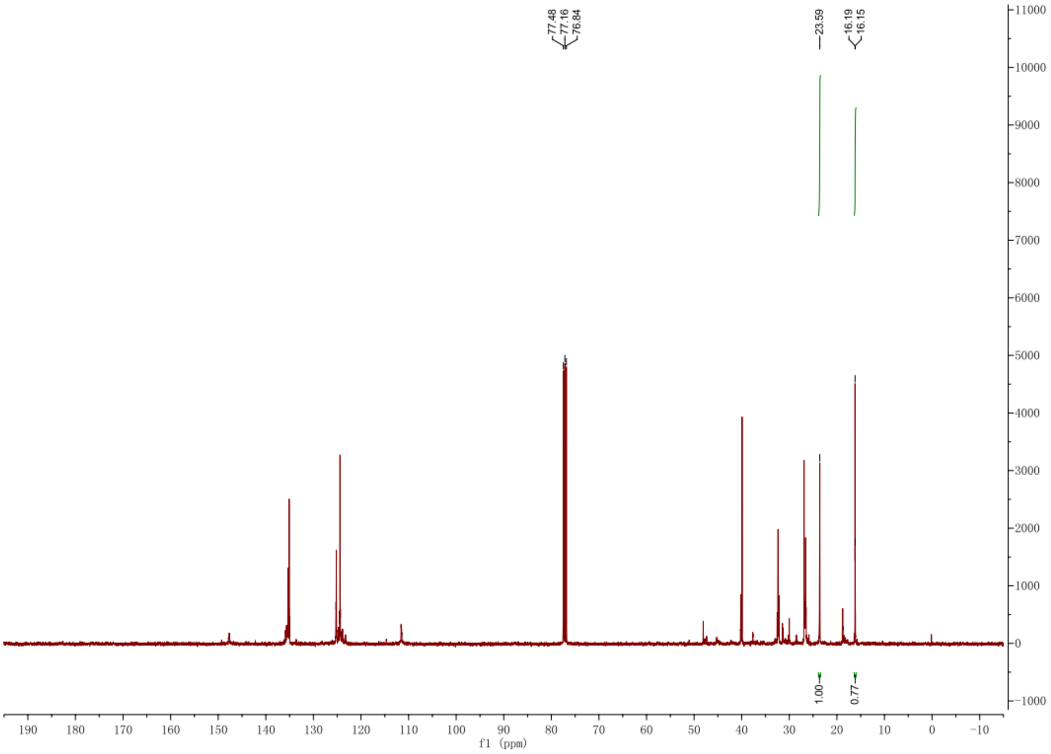

^{13}C NMR spectrum (100 MHz, $CDCl_3$, 25 ^{o}C)

Table 2, entry 3 (75% *cis*-1,4; 25% 3,4)

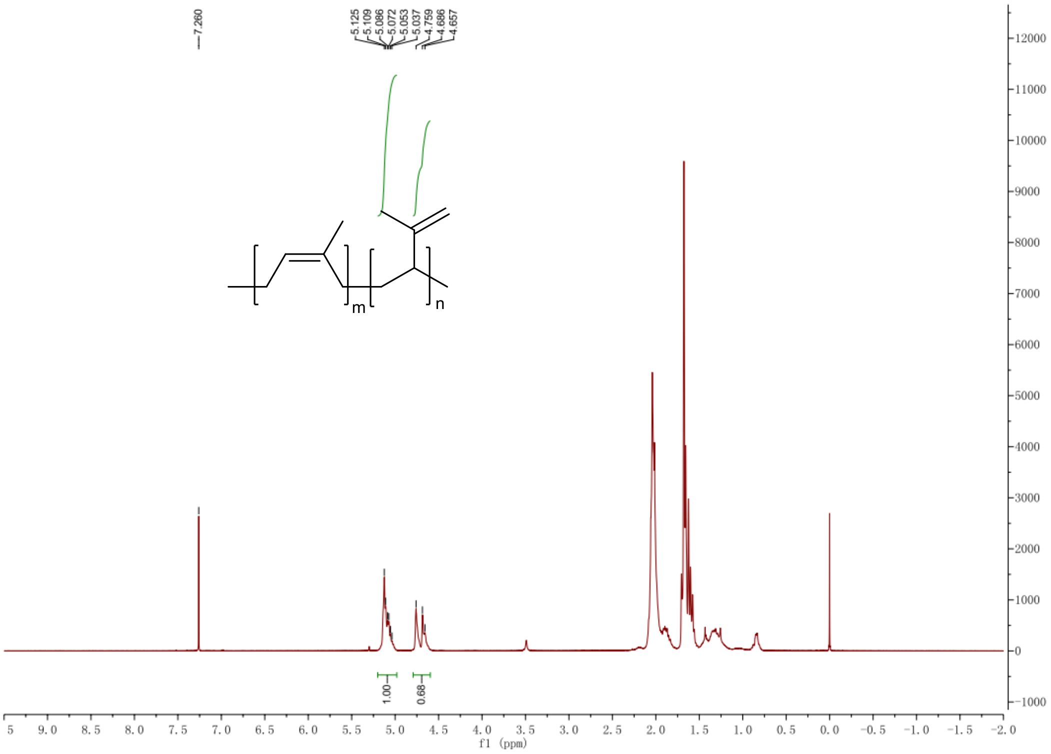

^{1}H NMR spectrum (400 MHz, $CDCl_3$, 25 ^{o}C)

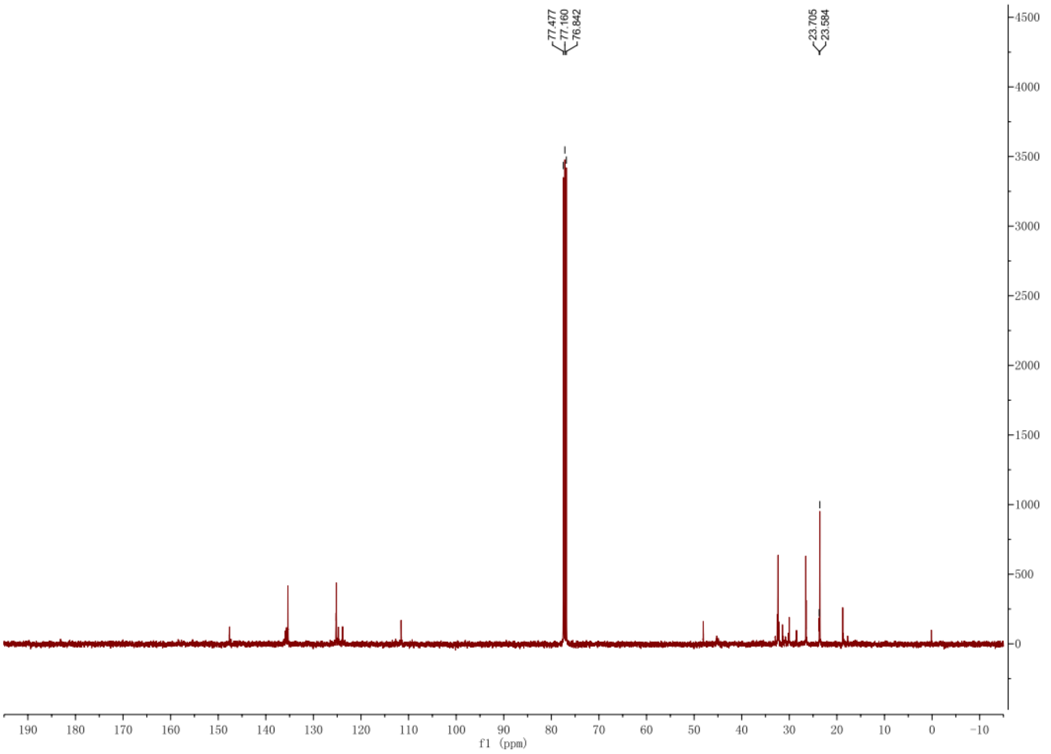

^{13}C NMR spectrum (100 MHz, $CDCl_3$, 25 ^{o}C)

Table 2, entry 4 (9% *trans*-1,4; 67% *cis*-1,4; 24% 3,4)

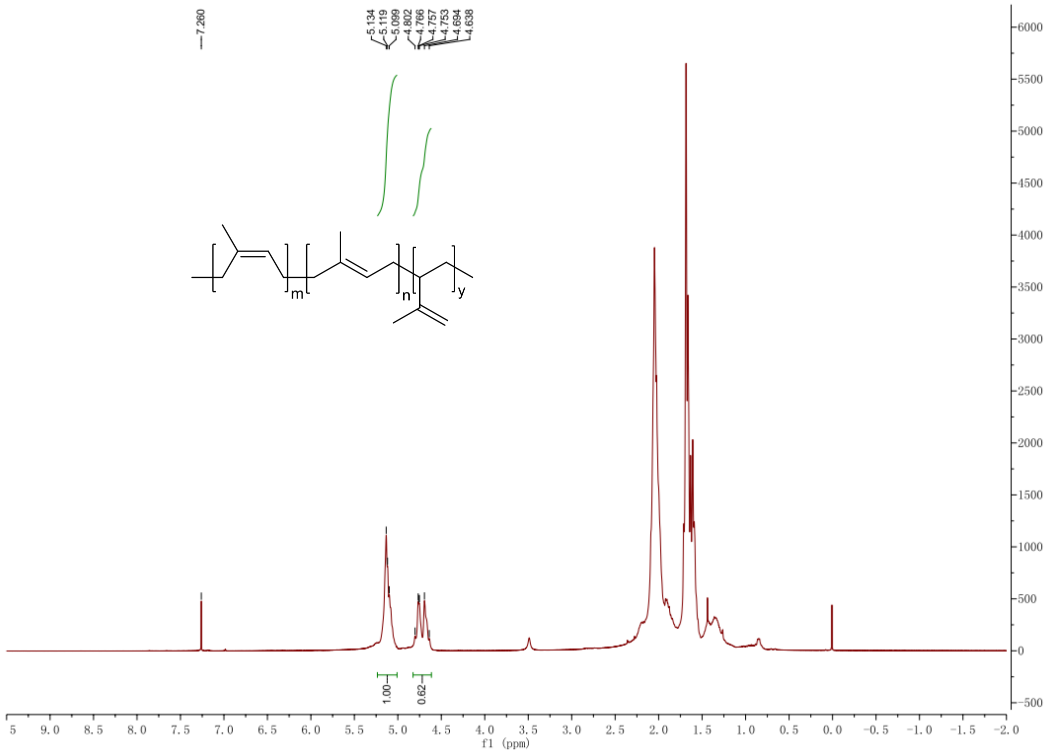

^{1}H NMR spectrum (400 MHz, $CDCl_3$, 25 ^{o}C)

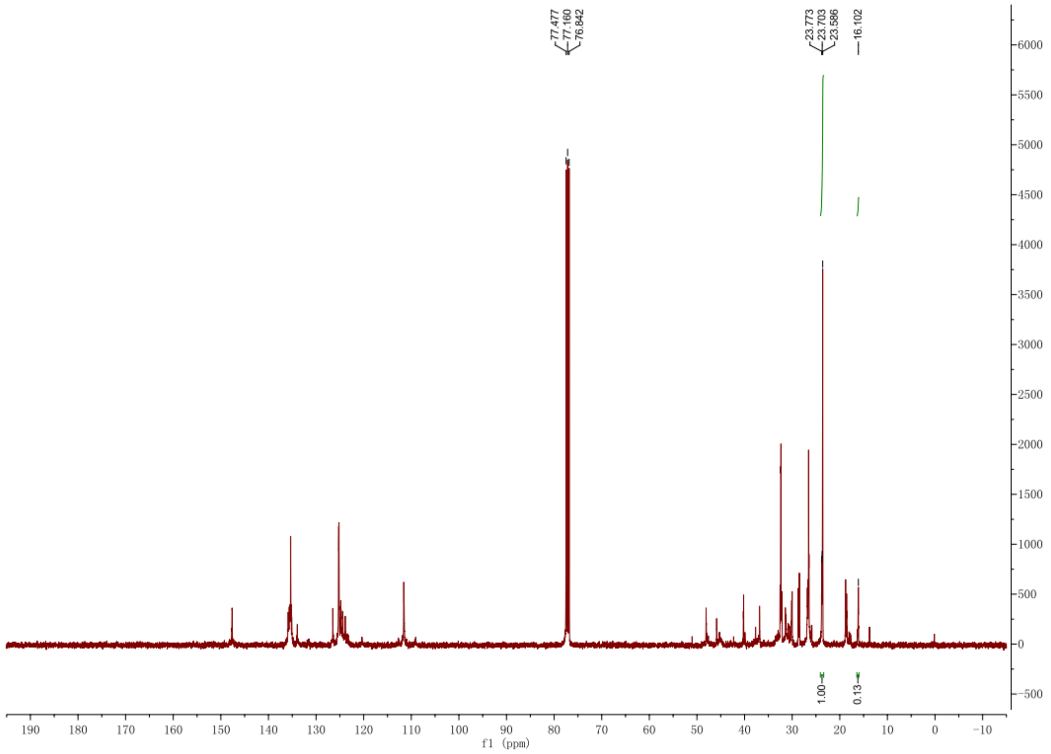

^{13}C NMR spectrum (100 MHz, $CDCl_3$, 25 ^{o}C)

Table 3, entry 1 (2% *trans*-1,4; 73% *cis*-1,4; 25% 3,4)

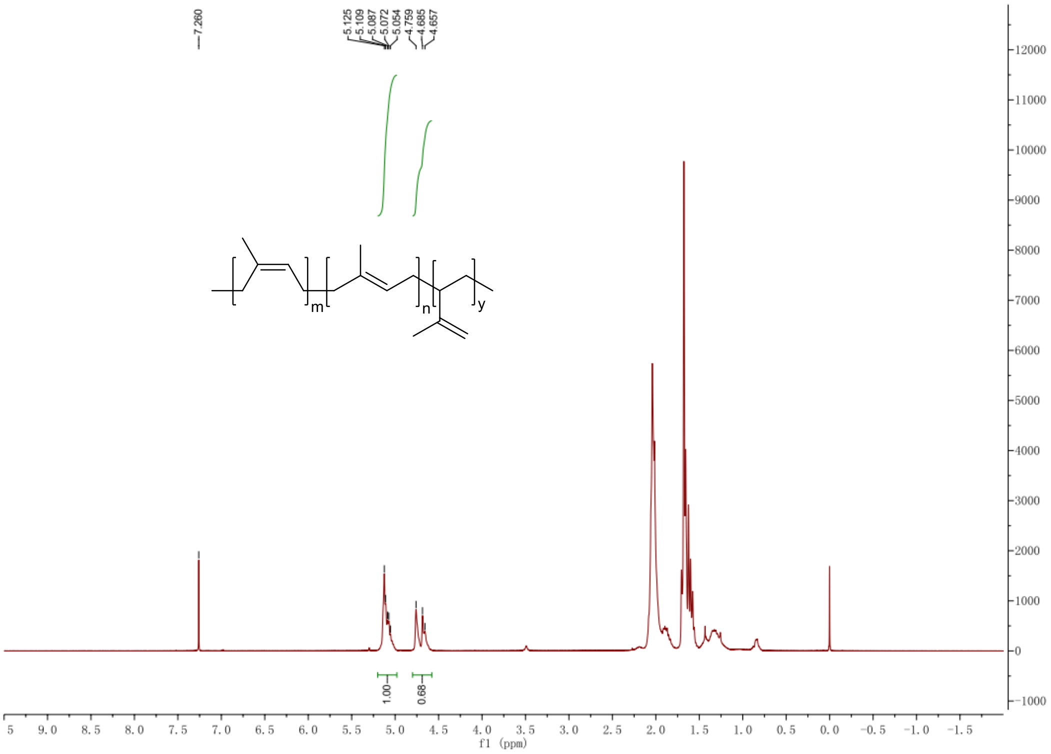

^{1}H NMR spectrum (400 MHz, $CDCl_3$, 25 ^{o}C)

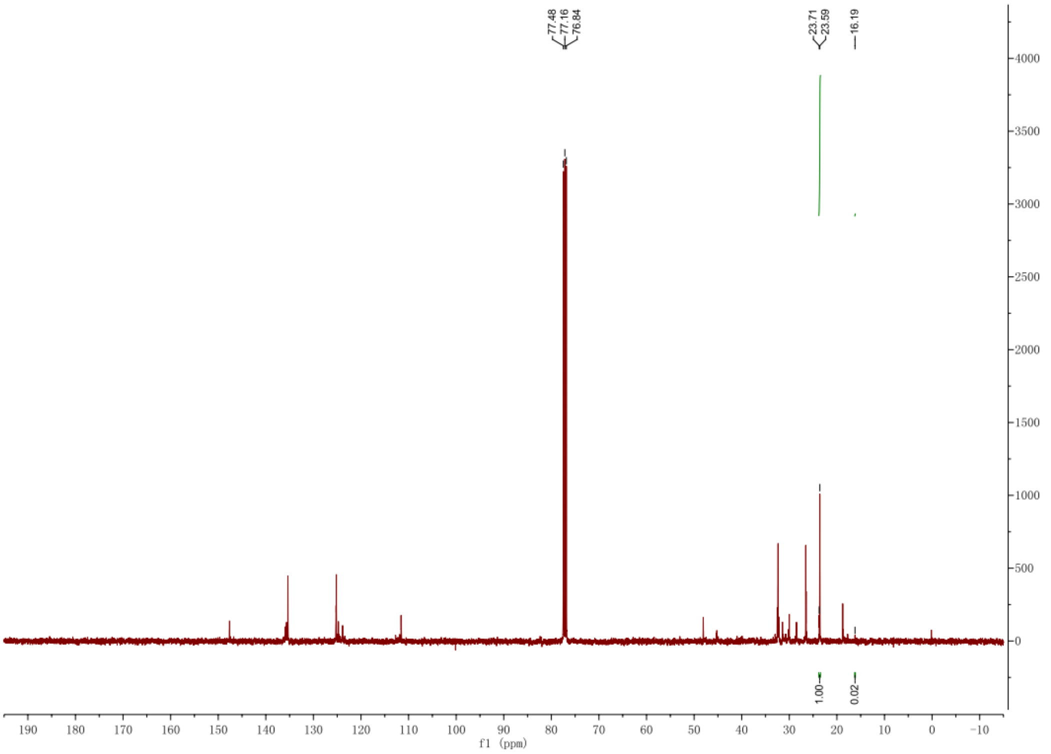

^{13}C NMR spectrum (100 MHz, $CDCl_3$, 25 ^{o}C)

Table 3, entry 2 (14% *trans*-1,4; 65% *cis*-1,4; 21% 3,4)

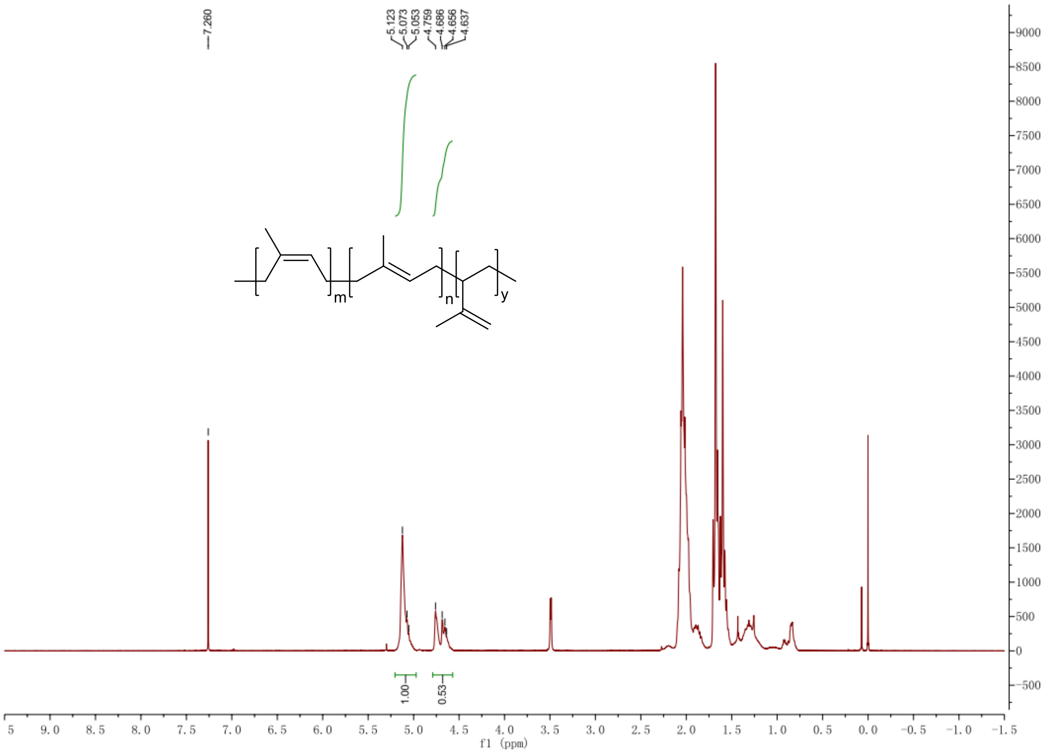

^{1}H NMR spectrum (400 MHz, $CDCl_3$, 25 °C)

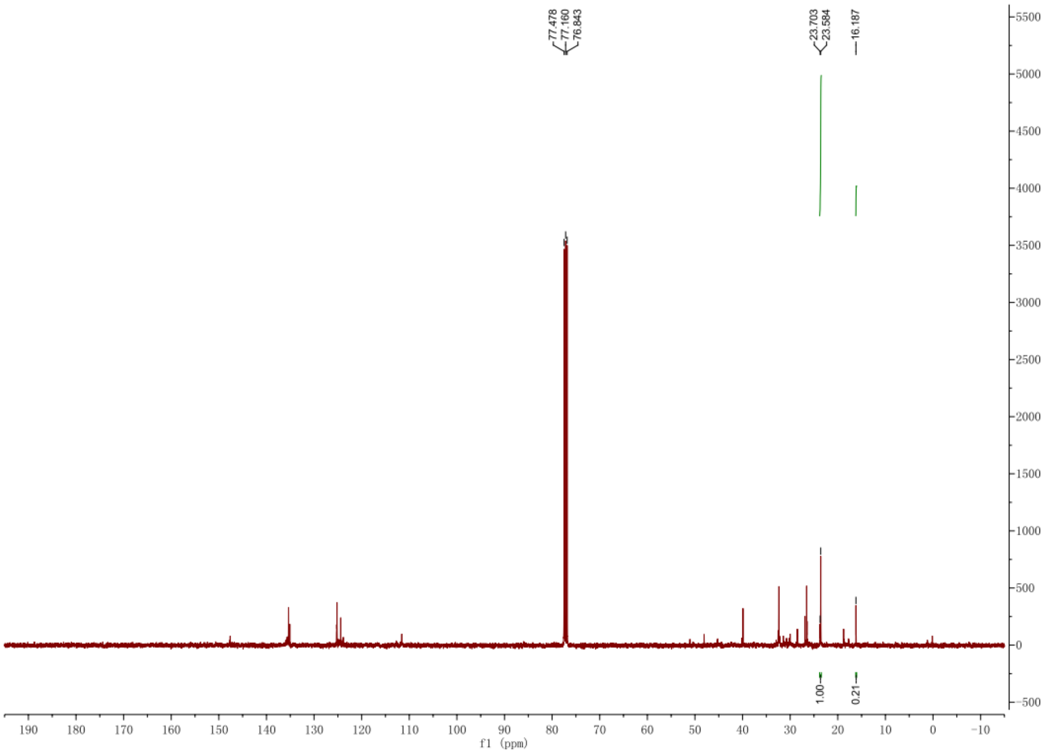

^{13}C NMR spectrum (100 MHz, $CDCl_3$, 25 °C)

Table 3, entry 3 (75% *cis*-1,4; 25% 3,4)

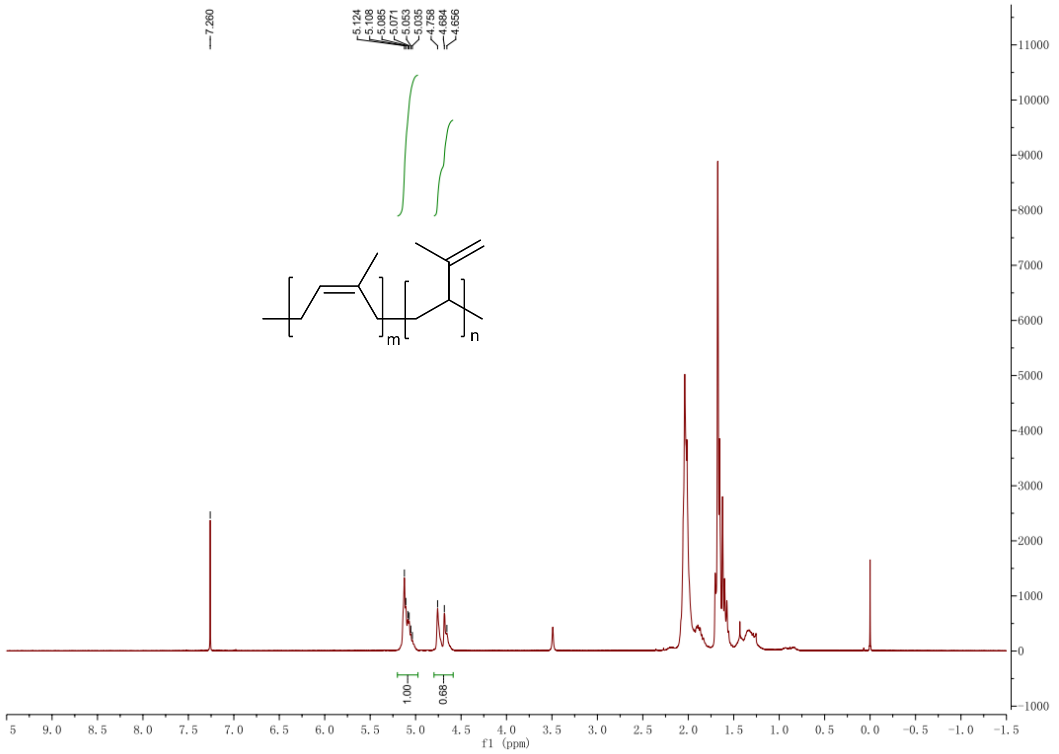

^1H NMR spectrum (400 MHz, $CDCl_3$, 25 °C)

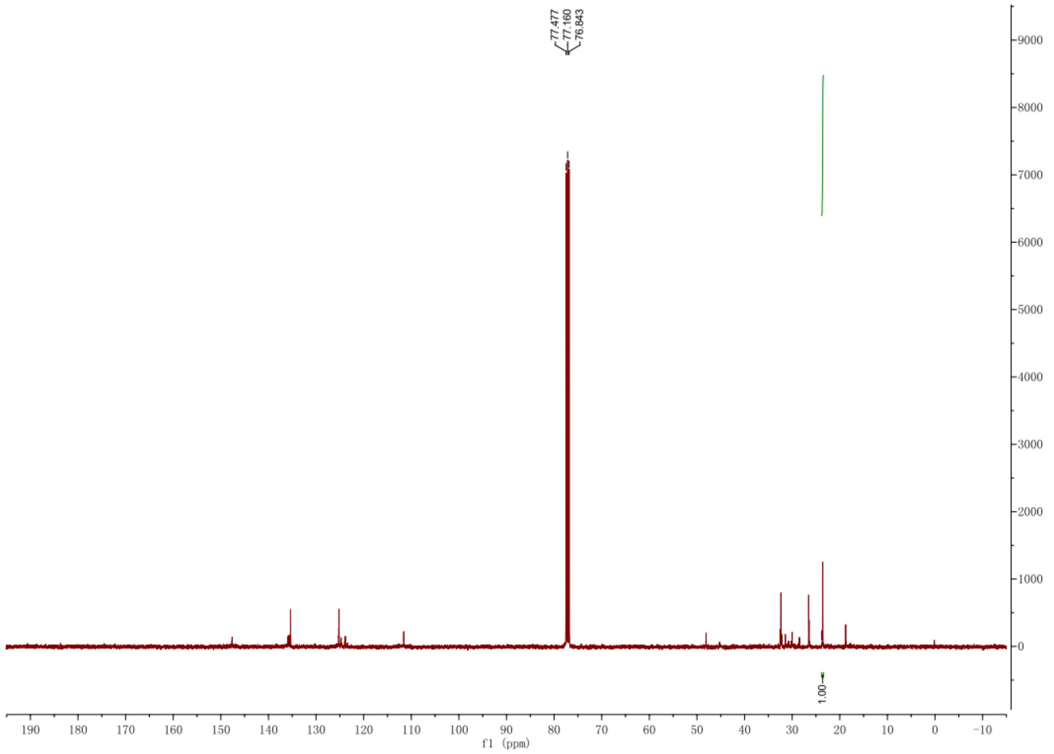

^{13}C NMR spectrum (100 MHz, $CDCl_3$, 25 °C)

Table 3, entry 4 (75% *cis*-1,4; 25% 3,4)

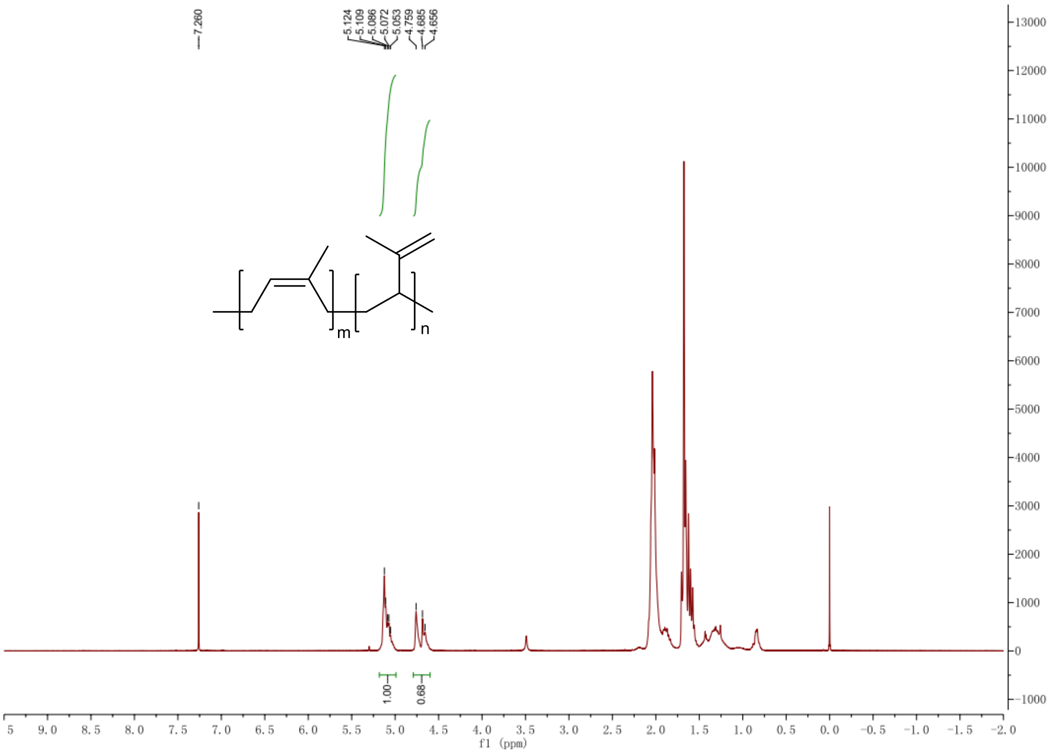

^{1}H NMR spectrum (400 MHz, $CDCl_3$, 25 ^{o}C)

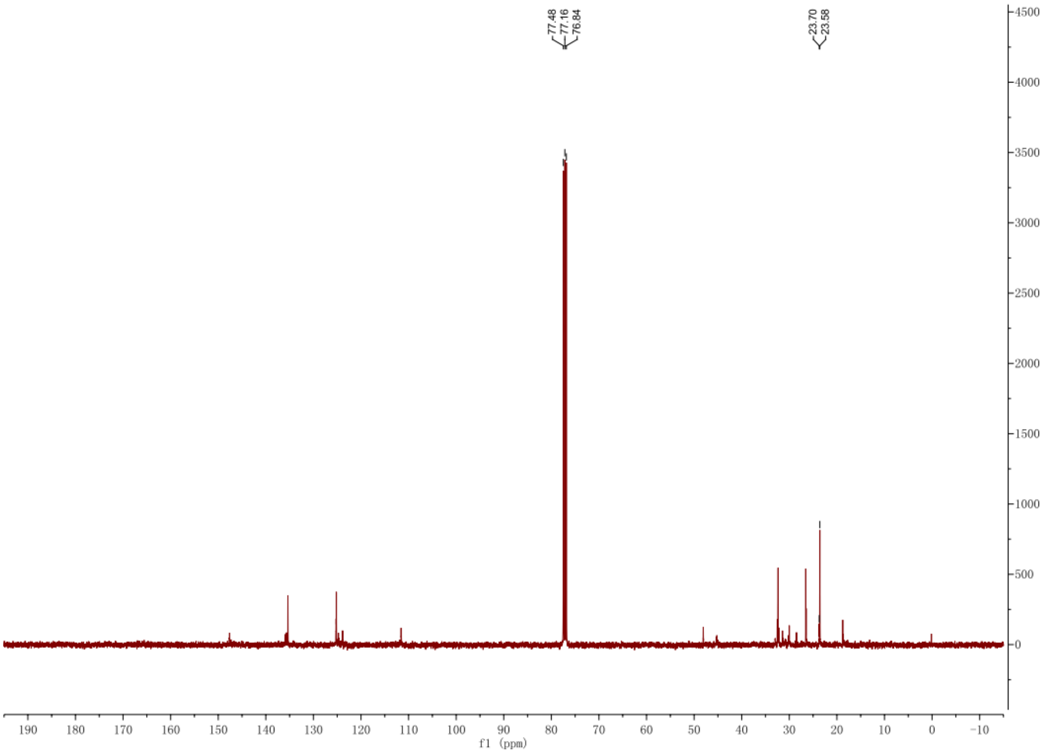

^{13}C NMR spectrum (100 MHz, $CDCl_3$, 25 ^{o}C)

Table 3, entry 5 (85% *trans*-1,4; 7% *cis*-1,4; 8% 3,4)

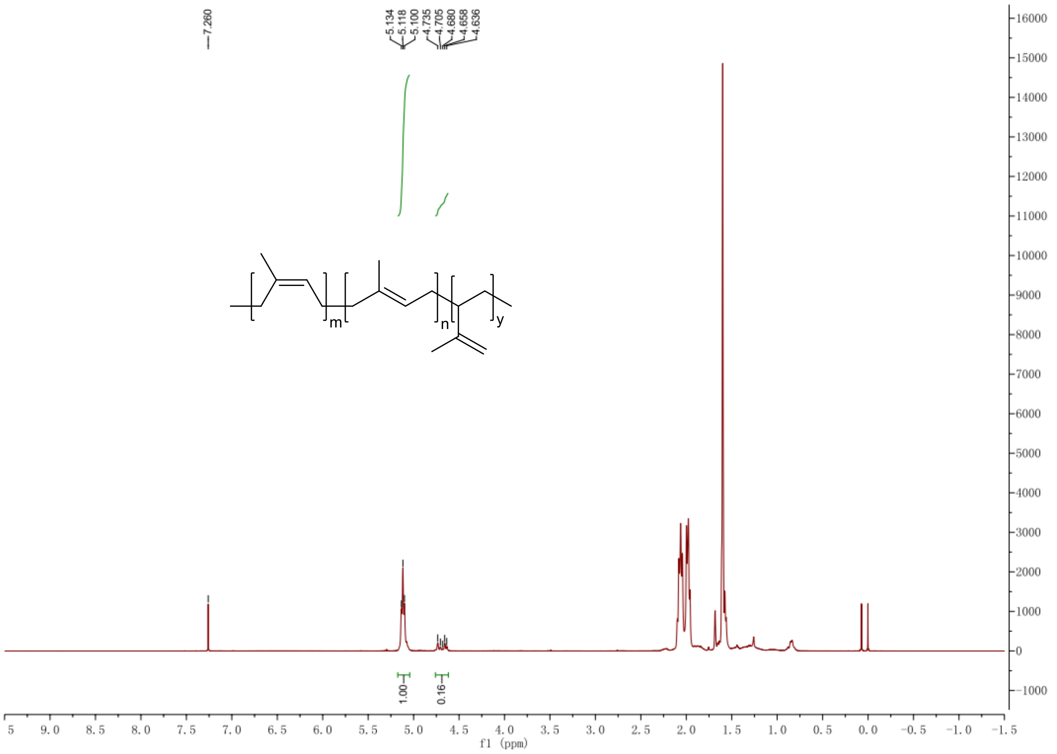

^{1}H NMR spectrum (400 MHz, $CDCl_3$, 25 °C)

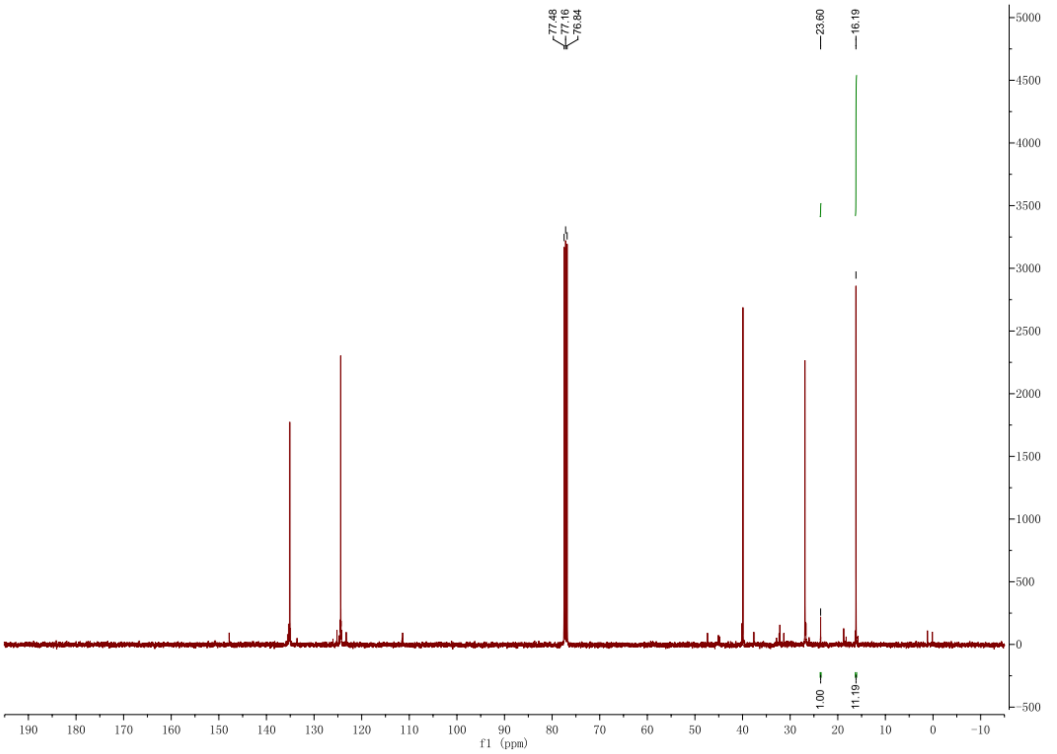

^{13}C NMR spectrum (100 MHz, $CDCl_3$, 25 °C)

Table 3, entry 6 (75% *cis*-1,4; 25% 3,4)

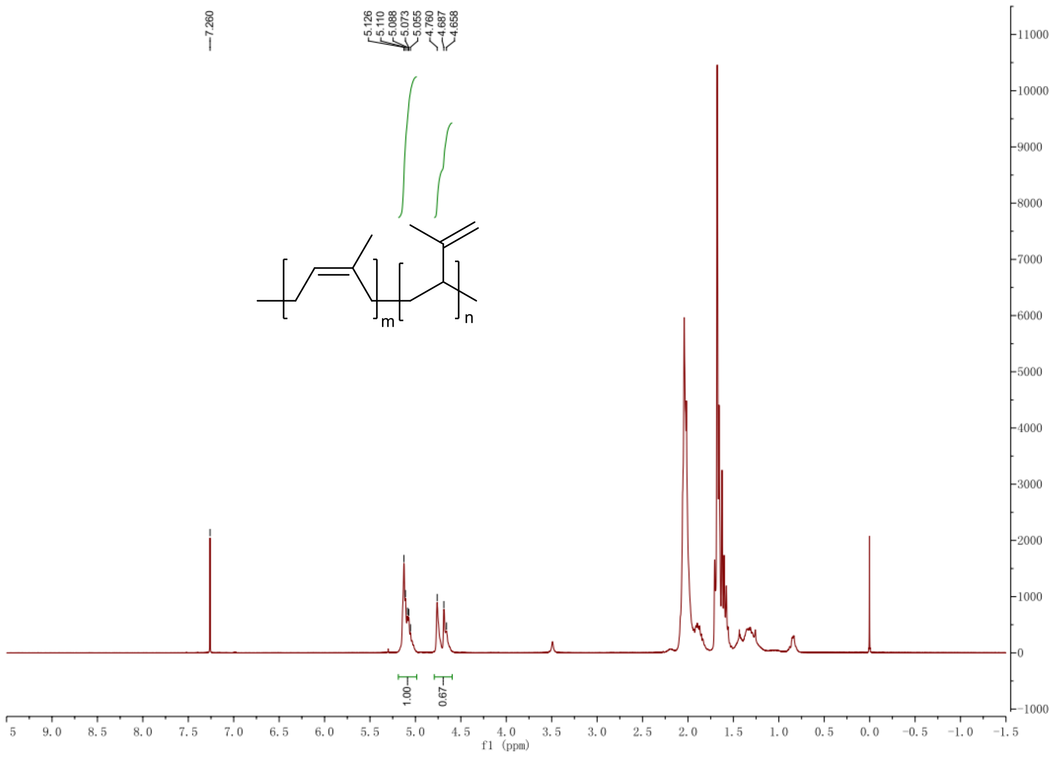

^{1}H NMR spectrum (400 MHz, $CDCl_3$, 25 ^{o}C)

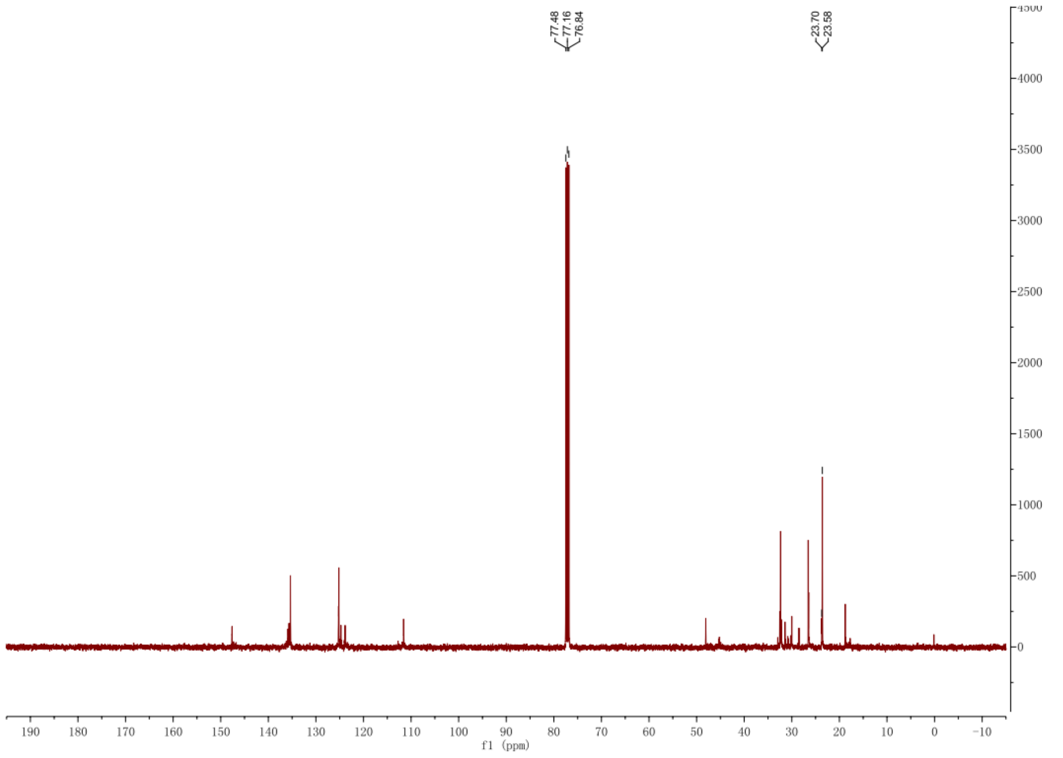

^{13}C NMR spectrum (100 MHz, $CDCl_3$, 25 ^{o}C)

Table 3, entry 7 (75% *cis*-1,4; 25% 3,4)

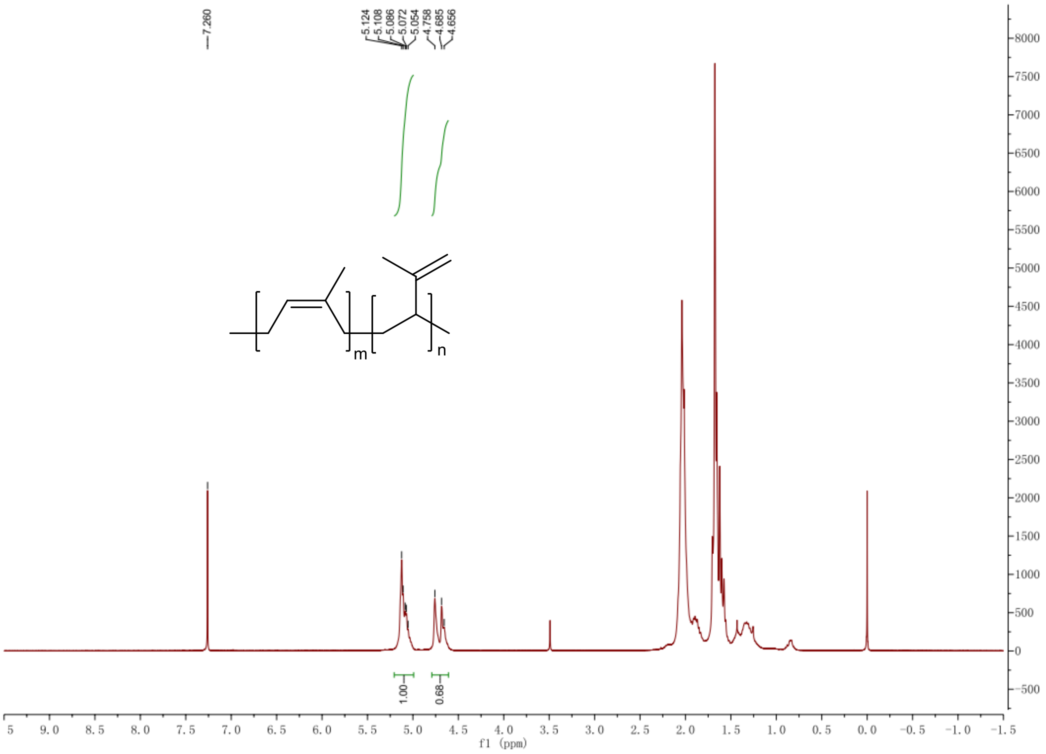

^{1}H NMR spectrum (400 MHz, $CDCl_3$, 25 ^{o}C)

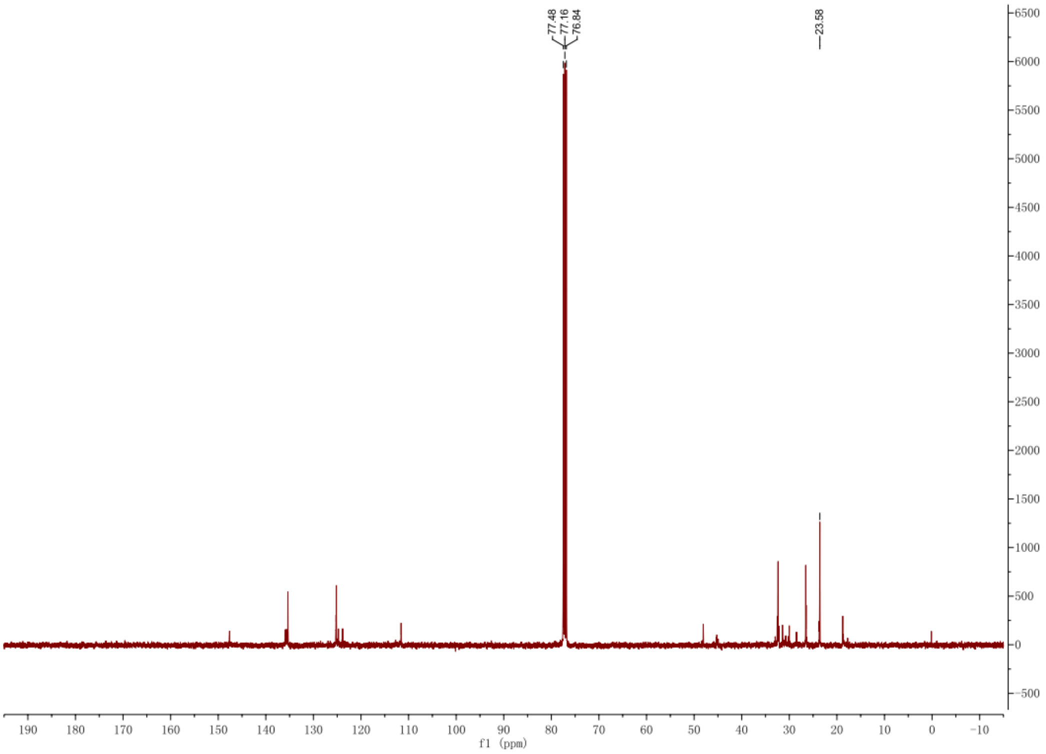

^{13}C NMR spectrum (100 MHz, $CDCl_3$, 25 ^{o}C)

Table S1, entry 1

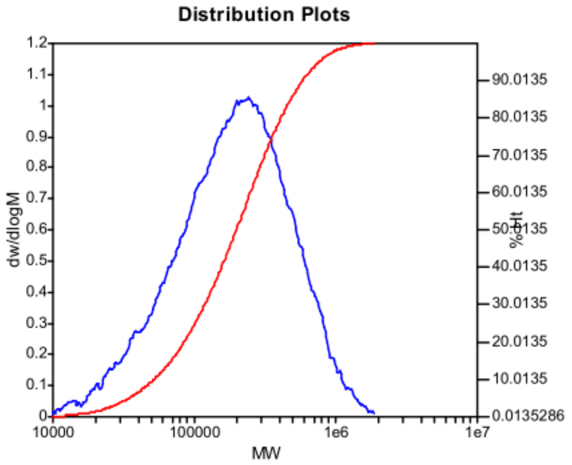

MW Averages

Peak No	Mp	Mn	Mw	Mz	Mz+1	Mv	PD
1	244505	115051	267603	484513	721578	238381	2.32595

Table S1, entry 2

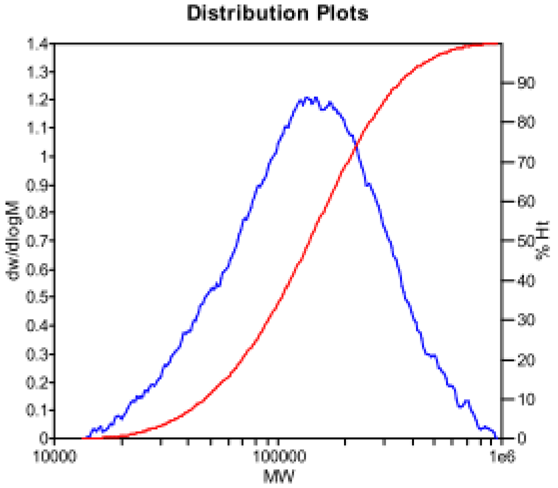

MW Averages

Peak No	Mp	Mn	Mw	Mz	Mz+1	Mv	PD
1	135023	97868	173388	275931	388258	161468	1.77165

Table S1, entry 3

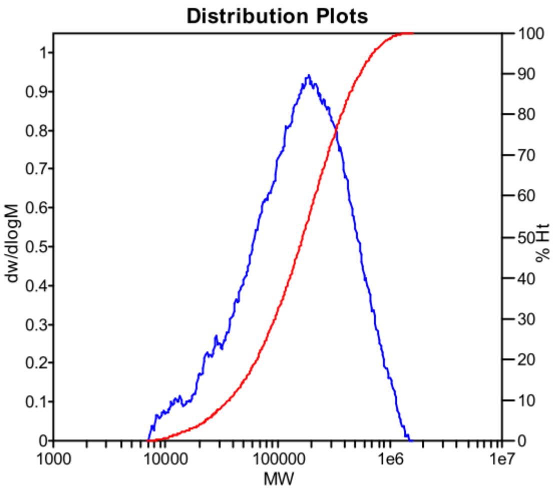

MW Averages

Peak No	Mp	Mn	Mw	Mz	Mz+1	Mv	PD
1	189538	84258	235722	443835	641372	206828	2.79762

Table S1, entry 5

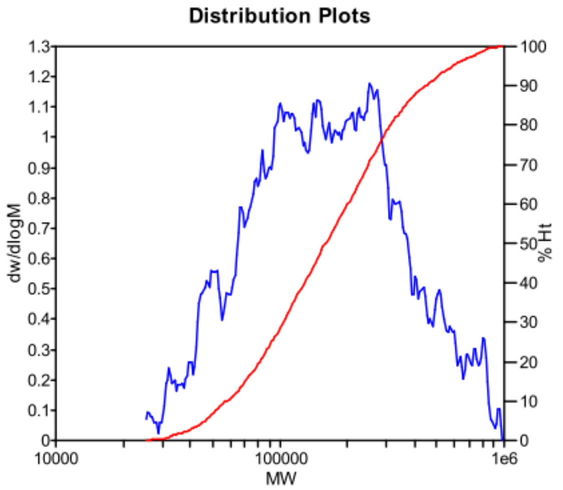

MW Averages

Peak No	Mp	Mn	Mw	Mz	Mz+1	Mv	PD
1	253165	120351	211906	348540	489912	196359	1.76073

[illegible]

Table 1, entry 1

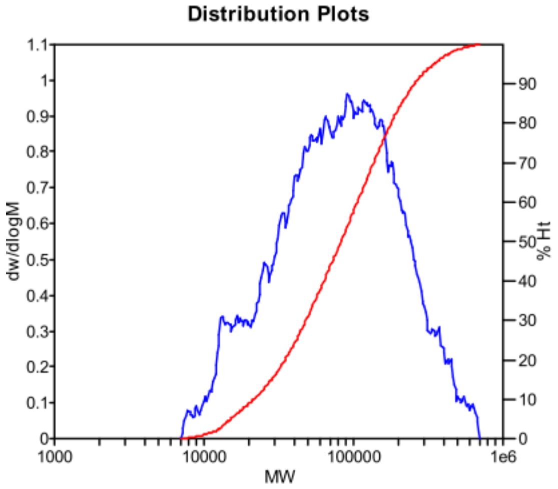

MW Averages

Peak No	Mp	Mn	Mw	Mz	Mz+1	Mv	PD
1	91342	50559	116064	216302	319915	102726	2.29562

Table 1, entry 2

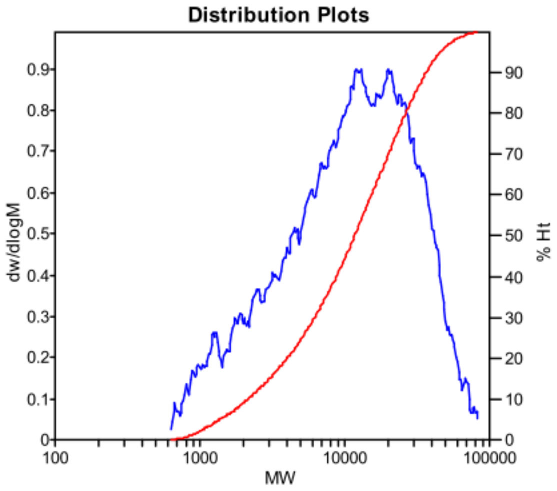

MW Averages

Peak No	Mp	Mn	Mw	Mz	Mz+1	Mv	PD
1	12917	5540	16037	29159	40112	14077	2.89477

Table 1, entry 3

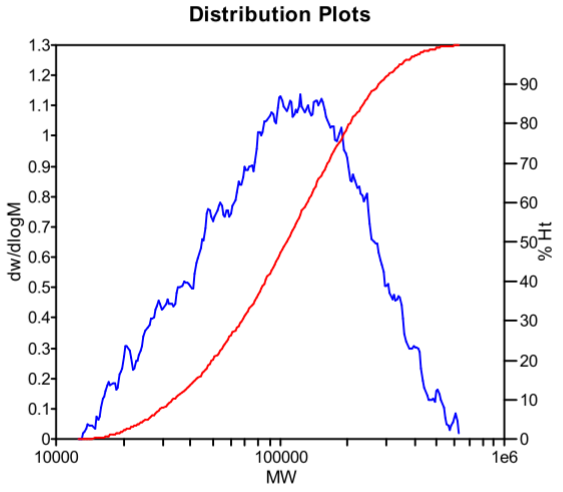

MW Averages

Peak No	Mp	Mn	Mw	Mz	Mz+1	Mv	PD
1	123988	73449	133985	211699	287301	122665	1.82419

Table 1, entry 4

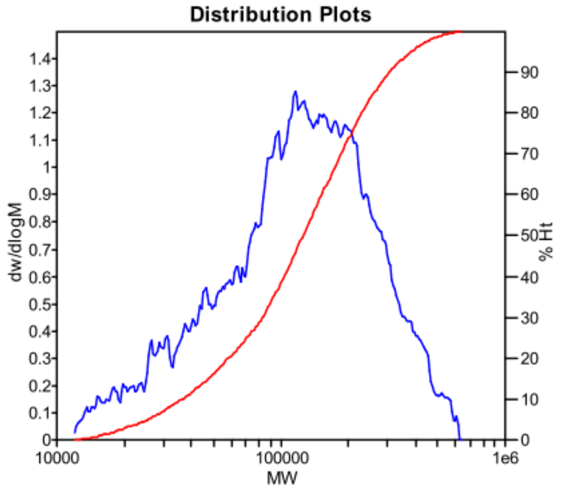

MW Averages

Peak No	Mp	Mn	Mw	Mz	Mz+1	Mv	PD
1	116345	80345	151344	231040	304473	141191	1.88368

Table 1, entry 5

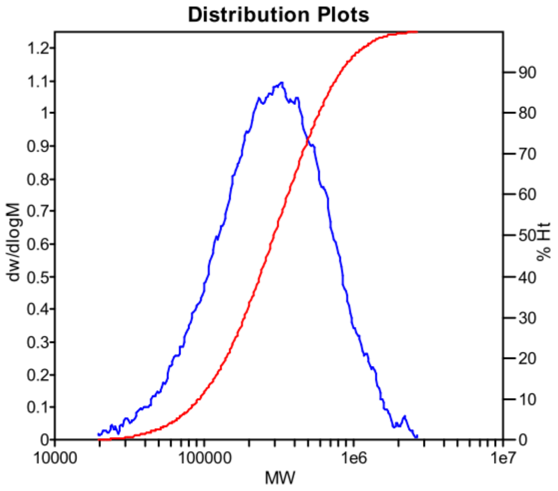

MW Averages

Peak No	Mp	Mn	Mw	Mz	Mz+1	Mv	PD
1	331892	193166	390739	682633	1027367	352328	2.02281

Table 1, entry 6

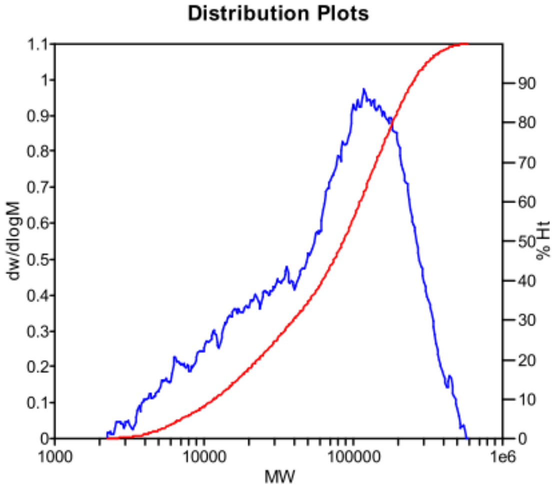

MW Averages

Peak No	Mp	Mn	Mw	Mz	Mz+1	Mv	PD
1	118286	30899	109828	197179	263812	98270	3.55442

Table 1, entry 7

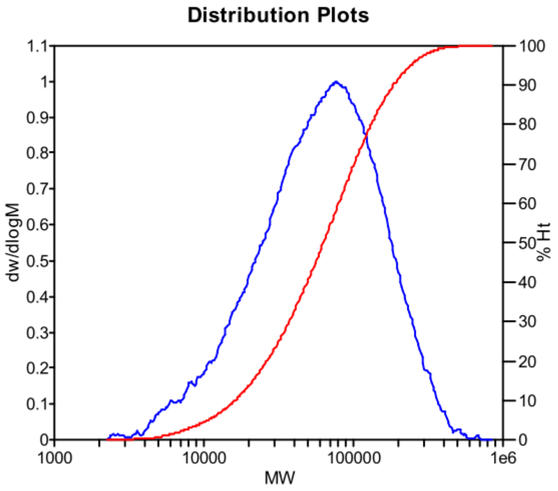

MW Averages

Peak No	Mp	Mn	Mw	Mz	Mz+1	Mv	PD
1	75676	35503	85305	154679	230920	77435	2.40275

Table 1, entry 8

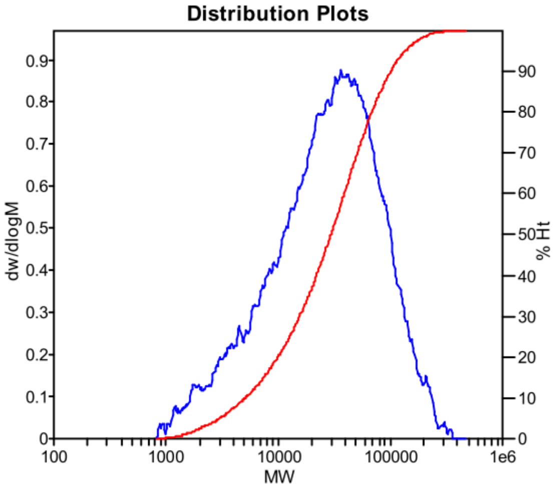

MW Averages

Peak No	Mp	Mn	Mw	Mz	Mz+1	Mv	PD
1	36521	12314	43535	90459	139785	37364	3.53541

Table 1, entry 9

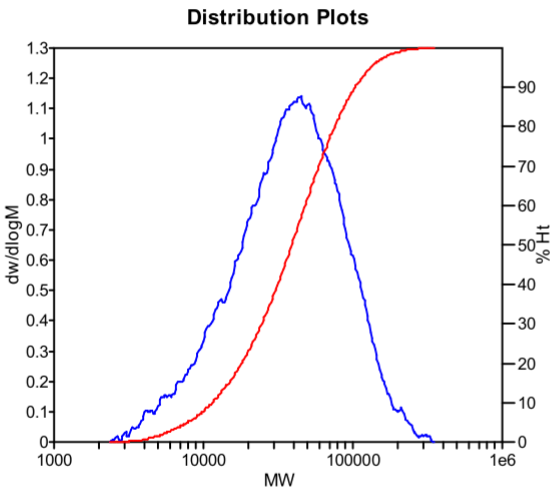

MW Averages

Peak No	Mp	Mn	Mw	Mz	Mz+1	Mv	PD
1	44966	24605	50030	84297	123180	45315	2.03333

Table 1, entry 10

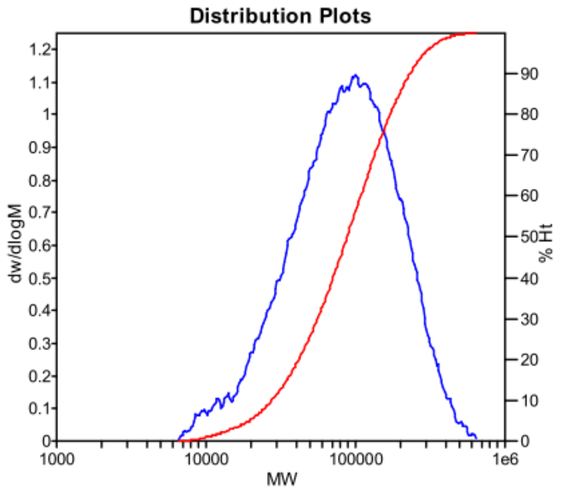

MW Averages

Peak No	Mp	Mn	Mw	Mz	Mz+1	Mv	PD
1	100384	57441	111660	181223	253266	101738	1.94391

Table 2, entry 1

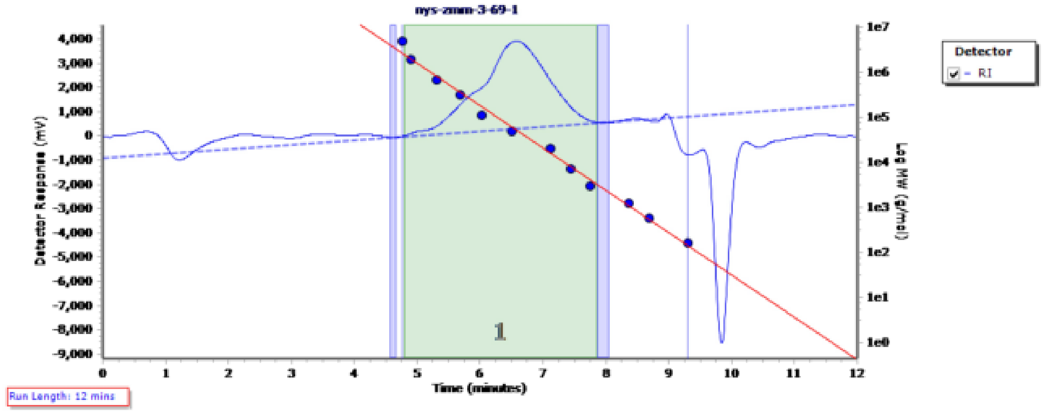

Molecular Weight Averages

Peak	Mp	Mn	Mw	Mz	Mz+1	Mv	PD
Peak 1	53151	34673	129035	499303	1072634	426915	3.721

Table 2, entry 2

Chromatogram

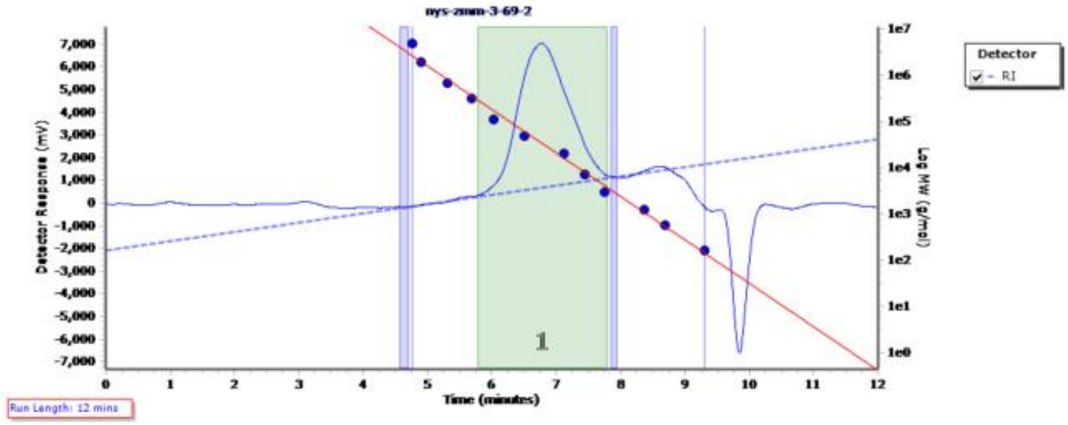

Molecular Weight Averages

Peak	Mp	Mn	Mw	Mz	Mz+1	Mv	PD
Peak 1	35082	23199	40536	65548	97020	61465	1.747

Table 2, entry 3

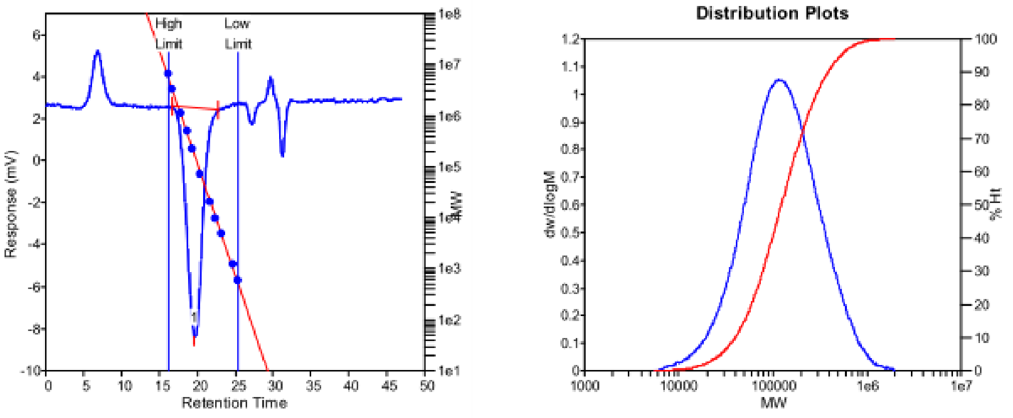

MW Averages

Peak No	Mp	Mn	Mw	Mz	Mz+1	Mv	PD
1	116151	81864	178514	355936	609478	161249	2.18062

Table 2, entry 4

Chromatogram

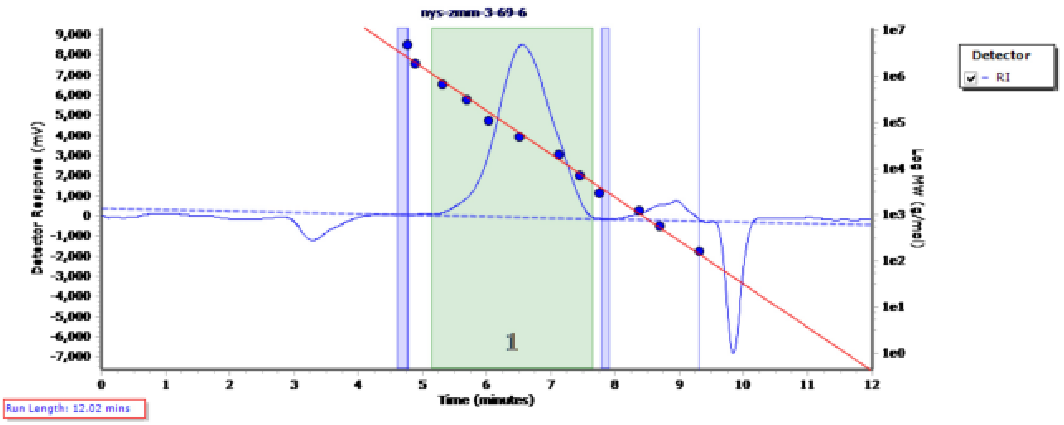

Molecular Weight Averages

Peak	Mp	Mn	Mw	Mz	Mz+1	Mv	PD
Peak 1	55107	35699	78428	184866	373946	163458	2.197

Table 3, entry 1

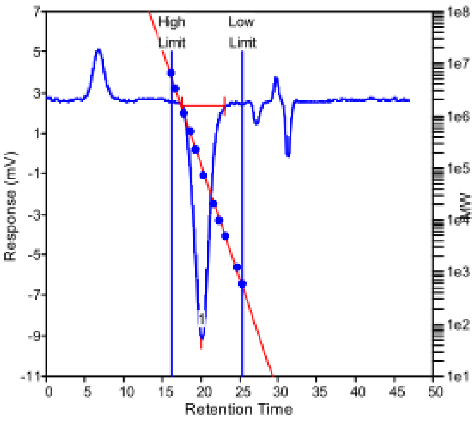

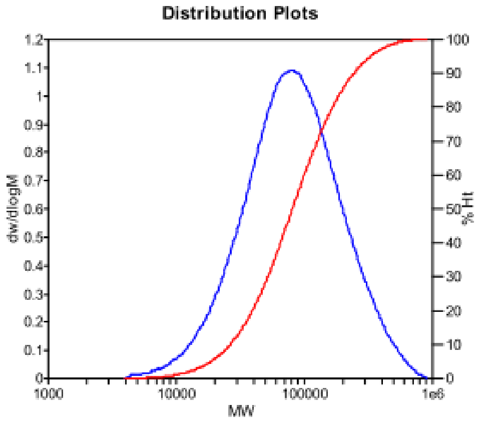

MW Averages

Peak No	Mp	Mn	Mw	Mz	Mz+1	Mv	PD
1	79848	54049	112886	209615	327339	102794	2.08859

Table 3, entry 2

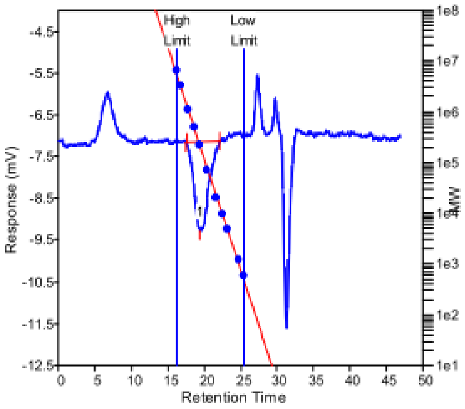

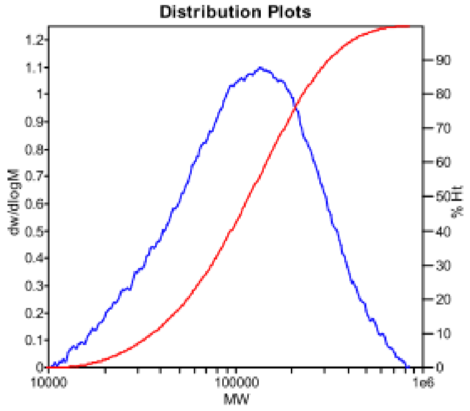

MW Averages

Peak No	Mp	Mn	Mw	Mz	Mz+1	Mv	PD
1	134496	78425	150673	246369	344126	139339	1.92124

Table 3, entry 3

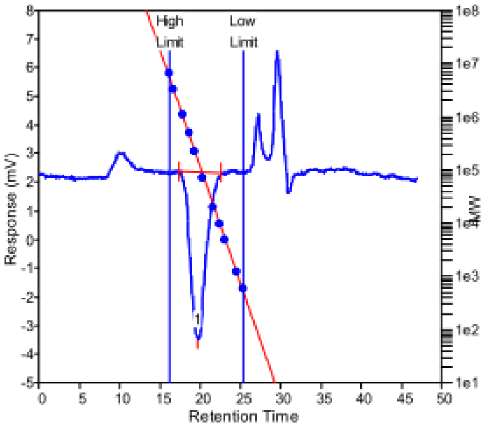

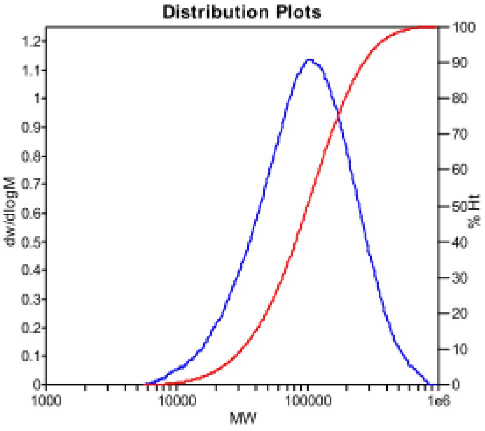

MW Averages

Peak No	Mp	Mn	Mw	Mz	Mz+1	Mv	PD
1	108822	65997	129852	219337	321792	119749	1.96754

Table 3, entry 4

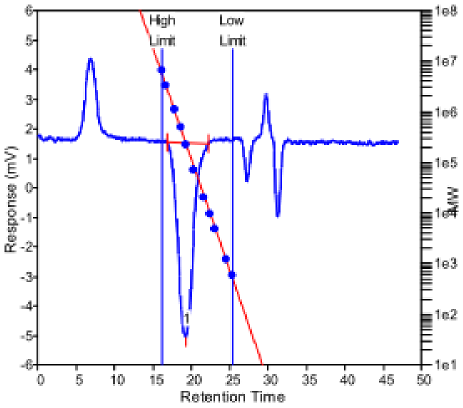

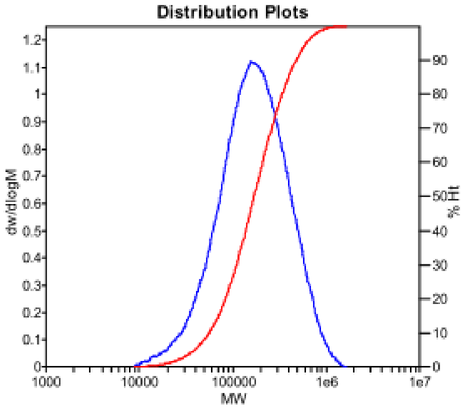

MW Averages

Peak No	Mp	Mn	Mw	Mz	Mz+1	Mv	PD
1	158298	112951	224833	382769	559415	207040	1.99054

Table 3, entry 5

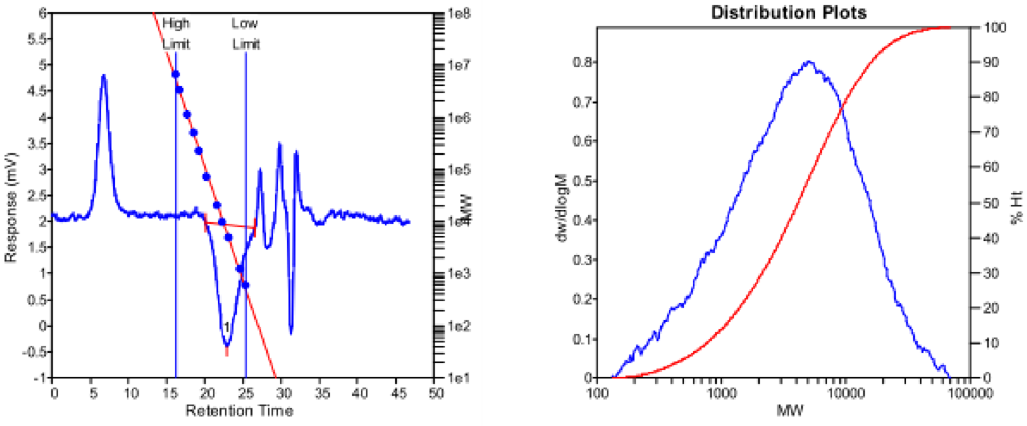

MW Averages

Peak No	Mp	Mn	Mw	Mz	Mz+1	Mv	PD
1	5085	1877	6763	15950	26680	5858	3.60309

Table 3, entry 6

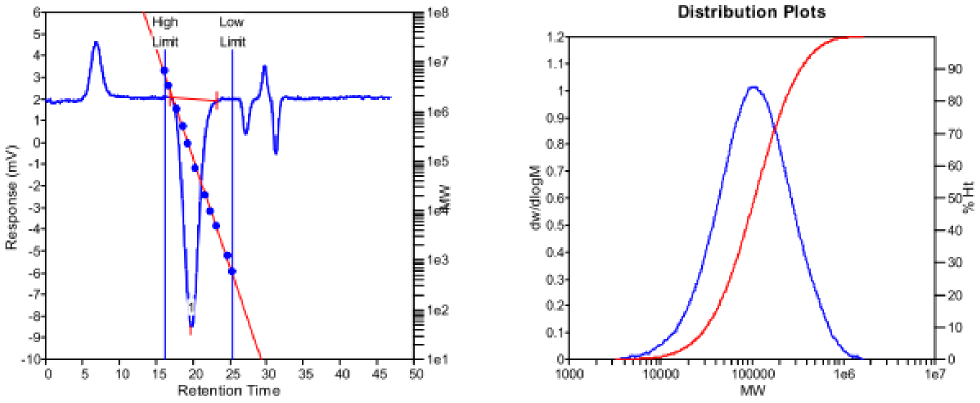

MW Averages

Peak No	Mp	Mn	Mw	Mz	Mz+1	Mv	PD
1	105333	66151	154804	309000	511155	139343	2.34016

Table 3, entry 7

Chromatogram

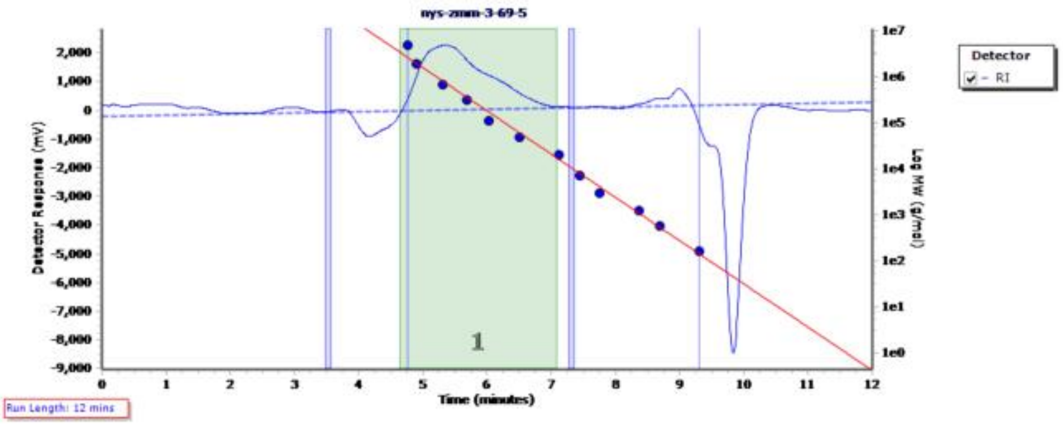

Molecular Weight Averages

Peak	Mp	Mn	Mw	Mz	Mz+1	Mv	PD
Peak 1	756321	238770	703372	1223624	1601720	1161782	2.946
